# Omicron spike function and neutralizing activity elicited by a comprehensive panel of vaccines

**DOI:** 10.1126/science.abq0203

**Published:** 2022-07-19

**Authors:** John E. Bowen, Amin Addetia, Ha V. Dang, Cameron Stewart, Jack T. Brown, William K. Sharkey, Kaitlin R. Sprouse, Alexandra C. Walls, Ignacio G. Mazzitelli, Jennifer K. Logue, Nicholas M. Franko, Nadine Czudnochowski, Abigail E. Powell, Exequiel Dellota, Kumail Ahmed, Asefa Shariq Ansari, Elisabetta Cameroni, Andrea Gori, Alessandra Bandera, Christine M. Posavad, Jennifer M. Dan, Zeli Zhang, Daniela Weiskopf, Alessandro Sette, Shane Crotty, Najeeha Talat Iqbal, Davide Corti, Jorge Geffner, Gyorgy Snell, Renata Grifantini, Helen Y. Chu, David Veesler

**Affiliations:** ^1^ Department of Biochemistry, University of Washington, Seattle, WA 98195, USA.; ^2^ Vir Biotechnology, San Francisco, CA 94158, USA.; ^3^ Howard Hughes Medical Institute, University of Washington, Seattle, WA 98195, USA.; ^4^ Instituto de Investigaciones Biomédicas en Retrovirus y SIDA (INBIRS), Facultad de Medicina, Buenos Aires C1121ABG, Argentina.; ^5^ Division of Allergy and Infectious Diseases, University of Washington, Seattle, WA 98195, USA.; ^6^ Departments of Paediatrics and Child Health and Biological and Biomedical Sciences, Aga Khan University, Karachi 74800, Pakistan.; ^7^ Humabs Biomed SA, a subsidiary of Vir Biotechnology, 6500 Bellinzona, Switzerland.; ^8^ Infectious Diseases Unit, Foundation IRCCS Ca’ Granda Ospedale Maggiore Policlinico, Milan, Italy.; ^9^ Department of Pathophysiology and Transplantation, University of Milan, Milan, Italy.; ^10^ Centre for Multidisciplinary Research in Health Science (MACH), University of Milan, Milan, Italy.; ^11^ Vaccine and Infectious Disease Division, Fred Hutchinson Cancer Research Center, Seattle, WA 98109, USA.; ^12^ Center for Infectious Disease and Vaccine Research, La Jolla Institute for Immunology, La Jolla, CA 92037, USA.; ^13^ Department of Medicine, Division of Infectious Diseases and Global Public Health, University of California, San Diego, La Jolla, CA 92037, USA.; ^14^ INGM, Istituto Nazionale Genetica Molecolare “Romeo ed Enrica Invernizzi,” Milan, Italy.

## Abstract

The severe acute respiratory syndrome coronavirus 2 (SARS-CoV-2) Omicron variant of concern comprises several sublineages, with BA.2 and BA.2.12.1 having replaced the previously dominant BA.1 and with BA.4 and BA.5 increasing in prevalence worldwide. We show that the large number of Omicron sublineage spike mutations leads to enhanced angiotensin-converting enzyme 2 (ACE2) binding, reduced fusogenicity, and severe dampening of plasma neutralizing activity elicited by infection or seven clinical vaccines relative to the ancestral virus. Administration of a homologous or heterologous booster based on the Wuhan-Hu-1 spike sequence markedly increased neutralizing antibody titers and breadth against BA.1, BA.2, BA.2.12.1, BA.4, and BA.5 across all vaccines evaluated. Our data suggest that although Omicron sublineages evade polyclonal neutralizing antibody responses elicited by primary vaccine series, vaccine boosters may provide sufficient protection against Omicron-induced severe disease.

## Boosting a weakened response

The Omicron strain of severe acute respiratory syndrome coronavirus 2 (SARS-CoV-2) was first detected in 2021 and is now dominant across the globe. Bowen *et al*. show that the Omicron sublineages BA.1, BA.2, BA.2.12, and BA.4/5 have increased affinity for the ACE2 receptor on human cells, but show decreased cell-cell fusion. They used pseudovirus to measure neutralizing activity against Omicron sublineages in plasma from individuals who were previously infected or vaccinated with one of seven vaccines, all based on the Wuhan-Hu-1 variant. For both vaccinees and convalescents, there was severe dampening of neutralizing activity. Boosting with either the same vaccine used initially or one of the other Wuhan-Hu-1–based vaccines increased neutralization across all vaccines evaluated. —VV

## Teaser

The increasingly prevalent Omicron BA.4/BA.5 sublineages exhibit enhanced receptor binding and escape from neutralizing antibodies.

**Fig. 1.**
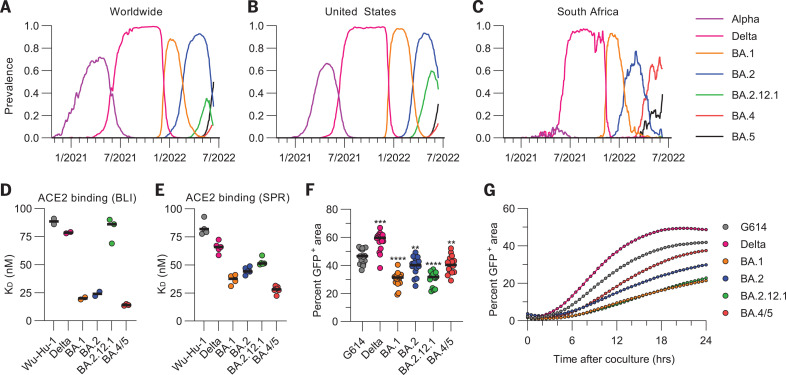
Omicron sublineage RBDs bind ACE2 with enhanced affinity but exhibit impaired S-mediated fusogenicity relative to the ancestral virus. (**A** to **C**) Prevalence of the different variants of concern measured globally (A), in the United States (B), or in South Africa (C). Alpha comprises B.1.1.7 and all Q sublineages; Delta comprises B.1.617.2 and all AY sublineages; and BA.1, BA.2, BA.4, and BA.5 comprise their respective sublineages (including BA.2.12.1 for BA.2). Prevalence calculations rely on shared GISAID (Global Initiative on Sharing Avian Influenza Data) sequences and may be biased by sampling. (**D** and **E**) Equilibrium dissociation constants (K_D_) of binding of the monomeric ACE2 ectodomain to immobilized biotinylated Wuhan-Hu-1, Delta, BA.1, BA.2, BA2.12.1, and BA.4/5 RBDs assessed by BLI (D) or SPR (E). Data presented are the results of at least two independent biological replicates for BLI and for SPR (except for the BA.1 RBD SPR data, which come from four technical replicates). (**F**) Quantification of cell-cell fusion after 24 hours mediated by Wuhan-Hu-1/G614, Delta, BA.1, BA.2, BA.2.12.1, and BA.4/5 S glycoproteins expressed as the fraction of the total area with GFP fluorescence assessed using a split GFP system. Data are from 16 fields of view from a single experiment and are representative of results obtained from two independent biological replicates. Comparisons between fusion mediated by the Wuhan-Hu-1/G614 S and other S variants were completed using the Wilcoxon rank sum test. **P < 0.01; ***P < 0.001; ****P < 0.0001. (**G**) Kinetics of cell-cell fusion mediated by Wuhan-Hu-1/G614, Delta, BA.1, BA.2, BA.2.12.1, and BA.4/5 S glycoproteins expressed as the fraction of the total area with GFP fluorescence assessed using a split GFP system.

The ongoing COVID-19 pandemic has led to the emergence of severe acute respiratory syndrome coronavirus 2 (SARS-CoV-2) variants with increased transmissibility, viral fitness, and immune evasion (*1*–*10*). The most recently named variant of concern, Omicron, is characterized by the greatest known genetic divergence from the ancestral virus (Wuhan-Hu-1) and consists of several sublineages, including BA.1, BA.2, BA.3, BA.4, and BA.5. BA.1 was first detected in late 2021 and rapidly replaced Delta to become the globally dominant SARS-CoV-2 strain (*3*, *9*, *11*), aided by its high transmissibility and escape from neutralizing antibodies (*6*, *12*–*18*). In early March of 2022, BA.2 became the most prevalent SARS-CoV-2 variant globally (*19*, *20*) (Fig. 1A), and the proportion of BA.2.12.1 in sequenced viruses peaked at >30% worldwide and >60% in the United States by late May of 2022 (Fig. 1B). However, BA.4 and BA.5, which share the same spike (S) glycoprotein sequence, are expected to reach global dominance owing to their increasing prevalence and successful replacement of BA.2 in South Africa (*21*) (Fig. 1C).

The receptor-binding domain (RBD) of the SARS-CoV-2 S glycoprotein interacts with the receptor angiotensin-converting enzyme 2 (ACE2) (*22*–*26*), promoting S conformational changes that lead to membrane fusion and viral entry (*27*–*29*). S is the main target of neutralizing antibodies, which have been shown to be a correlate of protection against SARS-CoV-2 (*30*–*38*), with RBD-targeting antibodies accounting for most neutralizing activity against vaccine-matched virus (*36*, *38*) and nearly all cross-variant neutralizing activity (*39*). SARS-CoV-2 vaccines are based on the S glycoprotein [sometimes the RBD only (*30*, *40*, *41*)] or (inactivated) virus, and they utilize a variety of delivery technologies. Lipid-encapsulated prefusion-stabilized S-encoding mRNA vaccines include Moderna mRNA-1273 and Pfizer-BioNTech BNT162b2. Viral-vectored vaccines encoding for the SARS-CoV-2 S sequence include Janssen Ad26.COV2.S (human adenovirus 26), AstraZeneca AZD1222 (chimpanzee adenovirus), and Gamaleya National Center of Epidemiology and Microbiology Sputnik V (human adenovirus 26 and 5 for prime and boost, respectively). Novavax NVX-CoV2373 is a prefusion-stabilized S protein subunit vaccine formulated with a saponin-based matrix M adjuvant, whereas Sinopharm BBIBP-CorV comprises inactivated virions. The primary vaccine series consisted of two doses for all of these vaccines except for Ad26.COV2.S, which was administered as a single dose.

We first aimed to understand how the different S mutations in the Omicron variant sublineages affect host receptor engagement and membrane fusion. Whereas the Delta RBD recognized human ACE2 with a comparable affinity to that of the Wuhan-Hu-1 RBD [1.1-fold enhancement by biolayer interferometry (BLI) (*3*) and 1.5-fold enhancement by surface plasmon resonance (SPR)], the ACE2 binding affinity was greater for the BA.1 RBD (4.4-fold by BLI and 2.6-fold by SPR) (*4*, *12*, *15*) and for the BA.2 RBD (3.7-fold by BLI and 2.3-fold by SPR) (Fig. 1, D and E; figs. S1 and S2; and tables S1 and S2). The BA.2.12.1 RBD—which differs from the BA.2 RBD only by the L452Q (substitution of leucine for glutamine at position 452) mutation—had an ACE2 binding affinity similar to that of the Wuhan-Hu-1 RBD (1.1-fold and 1.7-fold enhancements determined by BLI and SPR, respectively). The ACE2 binding affinity of the BA.4/BA.5 (BA.4/5) RBD was the greatest among the RBDs evaluated in this work, with 6.1-fold and 4.2-fold increases relative to Wuhan-Hu-1, as determined by BLI and SPR, respectively (Fig. 1, D and E; figs. S1 and S2; and tables S1 and S2).

We next compared the kinetics and magnitude of cell-cell fusion mediated by the Wuhan-Hu-1/G614, Delta, BA.1, BA.2, BA.2.12.2, and BA.4/5 S glycoproteins using a split green fluorescent protein (GFP) system (*42*) with VeroE6/TMPRSS2 (VeroE6 cells stably expressing TMPRSS2) target cells (expressing GFP β strands 1 to 10) and BHK-21 effector cells (expressing GFP β strand 11) and transiently transfected with S. We observed slower and markedly reduced overall fusogenicity for all tested Omicron sublineage S glycoproteins compared with Wuhan-Hu-1/G614 S and even more so relative to Delta S (*15*, *19*) (Fig. 1, F to H; fig. S3; and movies S1 to S6), despite the higher apparent BA.4/5 S cell surface expression compared with other S trimers (fig. S4).

**Fig. 2.**
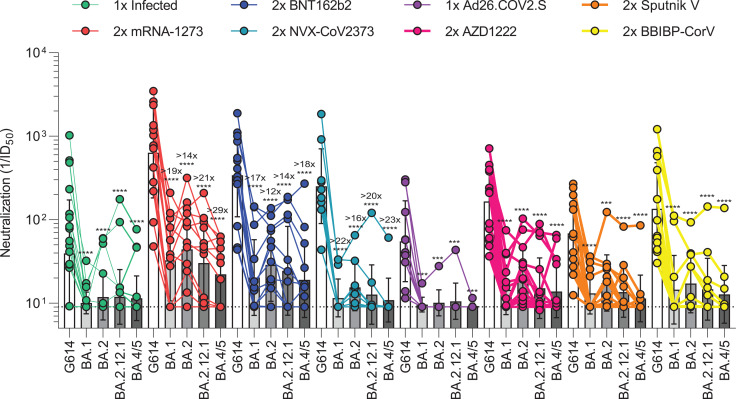
SARS-CoV-2 Omicron sublineages evade human plasma neutralizing antibodies elicited by infection or primary vaccine series. Plasma neutralizing antibody titers elicited by primary COVID-19 vaccination series determined using SARS-CoV-2 S VSV pseudotypes using VeroE6/TMPRSS2 as target cells. One-time (1×) infected samples (*n* = 24) were obtained 26 to 78 days (mean, 41) after symptom onset, two-dose (2×) mRNA-1273 samples (*n* = 14) were obtained 6 to 50 days (mean, 13) after second dose, 2× BNT162b2 samples (*n* = 14) were obtained 6 to 33 days (mean, 14) after second dose, 2× NVX-CoV2373 samples (*n* = 10) were obtained 17 to 168 days (mean, 82) after second dose, one-dose (1×) Ad26.COV2.S samples (*n* = 10) were obtained 9 to 142 days (mean, 79) after first dose, 2× AZD1222 samples (*n* = 16) were obtained ~30 days after second dose, 2× Sputnik V samples (*n* = 12) were obtained 60 to 90 days after second dose, and BBIBP-CorV samples (*n* = 12) were obtained 9 to 104 days (mean, 69) after second dose. Individual points are representative geometric mean titers from two independent experiments consisting of two replicates each. Bars represent geometric means, and error bars represent geometric standard deviations for each group. Statistical significance between groups of paired data was determined by Wilcoxon rank sum test. ***P < 0.001; ****P < 0.0001. Patient demographics are shown in table S4. Normalized curves and fits are shown in fig. S5. G614 indicates Wuhan-Hu-1/G614. ID_50_, median inhibitory dose.

We next assessed the plasma neutralizing activity elicited in humans by each of the seven vaccines or SARS-CoV-2 infection and evaluated the immune evasion associated with the constellation of S mutations present in the BA.1, BA.2, BA.2.12.1, and BA.4/5 Omicron sublineages (table S3). We measured entry of vesicular stomatitis virus (VSV) pseudotyped with the SARS-CoV-2 Wuhan-Hu-1 S harboring the D614G, BA.1, BA.2, BA.2.12.1, or BA.4/5 mutations into VeroE6/TMPRSS2 target cells (*43*) in the presence of vaccinee or convalescent plasma (table S4). Plasma was obtained from individuals previously infected with a Washington-1–like SARS-CoV-2 strain based on time of infection. These samples were obtained early in the pandemic, so individuals had not been vaccinated. We determined a plasma neutralizing geometric mean titer (GMT) of 39 against Wuhan-Hu-1/G614 VSV S pseudovirus, and only 5 of 24 individuals had detectable, albeit very weak, neutralizing activity against any of the four tested Omicron sublineages (Fig. 2 and fig. S5A). Plasma from subjects that received two doses of Moderna mRNA-1273 ~4 weeks apart had Wuhan-Hu-1/G614, BA.1, BA.2, BA.2.12.1, and BA.4/5 S VSV neutralizing GMTs of 633, 33, 44, 30, and 22, respectively, whereas plasma from subjects that received two doses of Pfizer BNT162b2 ~3 weeks apart had neutralizing GMTs of 340, 20, 29, 24, and 19, respectively (Fig. 2 and fig. S5, B and C). In total, 19 of 28, 21 of 28, 19 of 28, and 16 of 28 mRNA-vaccinated subjects retained neutralizing activity against BA.1, BA.2, BA.2.12.1, and BA.4/5 S VSV, respectively. The combined Moderna and Pfizer cohorts experienced ≥18-fold, ≥13-fold, ≥17-fold, and ≥23-fold GMT reductions against BA.1, BA.2, BA.2.12.1, and BA.4/5 S VSV, respectively. A similar trend was observed for plasma from individuals that received two doses of Novavax NVX-CoV2373 (*44*) in a double-blinded manner; however, these plasma samples were not obtained at peak titers owing to the design of the clinical trial (figs. S6 and S7). From this group, we determined a neutralizing GMT of 252 against Wuhan-Hu-1/G614 S VSV with only 2 of 10 individuals having detectable neutralizing activity against BA.1 (GMT: 12, ≥22-fold drop), 7 of 10 against BA.2 (GMT: 15, ≥16-fold drop), 4 of 10 against BA.2.12.1 (GMT: 13, ≥20-fold drop), and 1 of 10 against BA.4/5 (GMT: 11, ≥23-fold drop) (Fig. 2 and fig. S5D). Plasma from individuals vaccinated with Janssen Ad26.COV2.S were obtained 9 to 142 days (mean, 79) after their single dose—a time frame expected to capture peak neutralizing titers (*45*). This resulted in a Wuhan-Hu-1/G614 S VSV GMT of 55, and only 1 of 12 subjects had detectable plasma neutralizing activity against any of the Omicron sublineages (Fig. 2 and fig. S5E). Two doses of AZD1222 4 weeks apart induced Wuhan-Hu-1/G614, BA.1, BA.2, BA.2.12.1, and BA.4/5 S VSV neutralizing GMTs of 165, 14, 19, 15, and 14, respectively, with 13 of 16 and 4 of 16 individuals having detectable neutralizing activity against any or all tested subvariants, respectively (Fig. 2 and fig. S5F). Sputnik V vaccinee plasma after two doses had Wuhan-Hu-1/G614, BA.1, BA.2, BA.2.12.1, and BA.4/5 S VSV GMTs of 69, 13, 17, 14, and 11, respectively (Fig. 2 and fig. S5G). Detectable neutralizing activity against any or all Omicron sublineages was observed for 7 of 13 and 2 of 13 individuals, respectively. Finally, plasma from subjects vaccinated with two doses of Sinopharm BBIBP-CorV had a neutralizing GMT against G614 S VSV of 135, with 3 of 12 samples retaining detectable neutralizing activity against BA.1 (GMT: 14), 7 of 12 against BA.2 (GMT: 17), 5 of 12 against BA.2.12.1 (GMT: 15), and 4 of 12 against BA.4/5 (GMT: 11) (Fig. 2 and fig. S5H). Overall, these data underscore the magnitude of evasion of polyclonal plasma neutralizing antibody responses for Omicron sublineages in humans after primary vaccine series or infection [resulting from the accumulation of S mutations (*12*)], with a subtle but consistently more marked effect for BA.1 and even more so for BA.4/5 compared with BA.2 and BA.2.12.1.

**Fig. 3.**
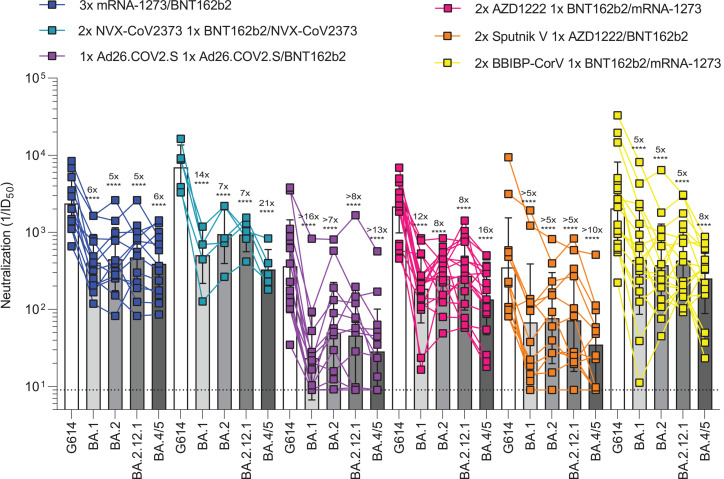
Administration of a booster dose rescues neutralization potency against Omicron sublineages for all vaccines. Plasma neutralizing antibody titers elicited by COVID-19 vaccine boosters determined using SARS-CoV-2 S VSV pseudotypes and VeroE6/TMPRSS2 as target cells. Three-dose (3×) mRNA-1273 or BNT162b2 samples (*n* = 13) were donated 13 to 97 days (mean, 30) after third dose; 2× NVX-CoV2373 plus 1× BNT162b2 or NVX-CoV2373 samples (*n* = 5) were donated 14 to 20 days (mean, 15) after third dose; 1× Ad26.COV2.S plus 1× Ad26.COV2.S or BNT162b2 samples (*n* = 14) were donated 12 to 16 days (mean, 14) after second dose; 2× AZD1222 plus 1× BNT162b2 or mRNA-1273 samples (*n* = 18) were donated 30 to 123 days (mean, 87) after third dose; 2× Sputnik V plus 1× AZD1222 or BNT162b2 samples (*n* = 14) were donated 45 to 60 days after third dose; and 2× BBIBP-CorV plus 1× BNT162b2 or mRNA-1273 samples (*n* = 18) were donated 29 to 89 days (mean, 50) after third dose. Individual points are representative geometric mean titers from two to four independent experiments consisting of two replicates each. Bars represent geometric means, and error bars represent geometric standard deviations for each group. Statistical significance between groups of paired data was determined by Wilcoxon rank sum test. **P < 0.01; ***P < 0.001; ****P < 0.0001. Patient demographics are shown in table S4. Normalized curves and fits are shown in fig. S8.

The emergence of the SARS-CoV-2 Delta and subsequently Omicron variants of concern led to an increasing number of reinfections and vaccine breakthrough cases (*5*, *46*–*48*). Public health policies were therefore updated worldwide to recommend administration of an additional vaccine dose (booster) several months after the primary vaccine series, which has been shown to increase the breadth and potency of neutralizing antibodies (*5*, *12*, *17*, *49*). We thus assessed and compared the benefits provided by homologous or heterologous vaccine boosters on vaccinee plasma neutralizing activity against Wuhan-Hu-1/G614, BA.1, BA.2, BA.2.12.1, and BA.4/5 S VSV pseudotypes. Plasma samples of subjects that received three mRNA vaccine doses had neutralizing GMTs of 2371, 406, 448, 472, and 392 against Wuhan-Hu-1/G614, BA.1, BA.2, BA.2.12.1, and BA.4/5 S VSV, respectively (Fig. 3 and fig. S8A). The five- to sixfold potency losses against these variants are marked improvements over the >13- to >23-fold reductions observed after two vaccine doses, underscoring an increase in overall neutralizing antibody potency and breadth (*5*, *12*). Individuals vaccinated with two doses of NVX-CoV2373 followed by a booster of mRNA-1273 (1 of 5 individuals) or NVX-CoV2373 (4 of 5 individuals) had neutralizing GMTs of 6978 for Wuhan-Hu-1/G614, 505 for BA.1 (14-fold reduction), 948 for BA.2 (sevenfold reduction), 935 for BA.2.12.1 (sevenfold reduction), and 330 for BA.4/5 (21-fold reduction) (Fig. 3 and fig. S8B). Plasma from individuals who received one dose of Ad26.COV2.S followed by either a homologous Ad26.COV2.S (12 of 14 individuals) or a heterologous BNT162b2 booster (2 of 14 individuals) ~4 months later had neutralizing GMTs of 363, 23, 50, 46, and 29 against Wuhan-Hu-1/G614, BA.1, BA.2, BA.2.12.1, and BA.4/5 S VSV, respectively, corresponding to dampening ranging between ≥7- and ≥16-fold with 9 of 14 individuals maintaining neutralizing activity against all sublineages (Fig. 3 and fig. S8C). We also investigated individuals that received two doses of AZD1222 4 weeks apart followed by a BNT162b2 (17 of 18 individuals) or mRNA-1273 (1 of 18 individuals) booster ~6 months later. This cohort had respective neutralizing GMTs of 2167, 186, 269, 273, and 135 against Wuhan-Hu-1/G614, BA.1, BA.2, BA.2.12.1, and BA.4/5 S VSV, corresponding to 8- to 16-fold potency reductions (Fig. 3 and fig. S8D). Individuals vaccinated with two doses of Sputnik V and boosted with AZD1222 (11 of 12 individuals) or BNT162b2 (1 of 12 individuals) ~9 months later had neutralizing GMTs of 351, 68, 77, 72, and 35 for Wuhan-Hu-1/G614, BA.1, BA.2, BA.2.12.1, and BA.4/5, respectively, amounting to 5- to 10-fold reductions of potency (Fig. 3 and fig. S8E). BBIBP-CorV vaccinees boosted with either BNT162b2 (14 of 18 individuals) or mRNA-1273 (4 of 18 individuals) had GMTs of 2047 for G614, 439 for BA.1 (fivefold reduction), 375 for BA.2 (fivefold reduction), 430 for BA.2.12.1 (fivefold reduction), and 252 for BA.4/5 (eightfold reduction) (Fig. 3 and fig. S8F). The marked improvement in plasma neutralizing activity for subjects that received a booster dose over those that did not highlights the importance of vaccine boosters for eliciting potent neutralizing antibody responses against Omicron sublineages.

To assess the effect of target cell lines on apparent Omicron immune escape, we compared the aforementioned VeroE6/TMPRSS2 cells (*43*) with a stable ACE2-overexpressing HEK293T cell line (HEK293T/ACE2) (*50*) to determine plasma neutralizing activity for a cohort of mRNA-vaccinated individuals. After primary vaccine series, only three subjects had detectable neutralizing activity against any of the tested Omicron sublineage VSV pseudotypes when using HEK293T/ACE2 target cells. By contrast, all but one subject had detectable, albeit very weak, neutralizing activity against Omicron VSV pseudotypes using VeroE6/TMPRSS2 target cells, resulting in >17-fold, >14-fold, >20-fold, and >22-fold reductions against BA.1, BA.2, BA.2.12.1, and BA.4/5, respectively (figs. S9A and S10A). After a booster dose, we observed respective 7-fold, 7-fold, 11-fold, and 13-fold reductions of neutralizing activity against BA.1, BA.2, BA.2.12.1, and BA.4/5 VSV pseudotypes using HEK293T/ACE2 target cells, as compared with respective sevenfold, sixfold, fivefold, and eightfold reductions when using VeroE6/TMPRSS2 target cells (figs. S9B and S10B). This indicates that the target cell lines used in neutralization assays may affect the observed plasma neutralizing escape of SARS-CoV-2 variants, which may be further compounded on the basis of preferential entry routes (*15*, *23*, *51*).

We report that the BA.1, BA.2, BA.2.12.1, and BA.4/5 Omicron sublineages, which account for >99% of all infections worldwide over the first half of 2022, have increased ACE2 binding affinity, have decreased fusogenicity, and markedly evade neutralizing antibody responses relative to the Wuhan-Hu-1 and Delta strains (*3*). Collectively, these data suggest that enhanced receptor engagement and immune evasion are key changes that may have promoted the rapid spread of these Omicron sublineages and could contribute to the current rise in prevalence of BA.4 and BA.5.

The development of life-saving vaccines is regarded as one of humanity’s greatest medical and scientific achievements, which is exemplified by COVID-19 vaccines (*52*–*54*). Primary COVID-19 vaccine regimens or infection-elicited plasma neutralizing activity was severely dampened by Omicron sublineages BA.1, BA.2, BA.2.12.1, and BA.4/5. However, administration of a booster dose increased neutralizing antibody titers and breadth against all Omicron sublineages to appreciable levels regardless of the vaccine evaluated, concurring with findings for BA.1 (*5*, *12*, *15*, *17*, *49*, *55*, *56*). These results are consistent with previous studies demonstrating that a third vaccine dose results in the recall and expansion of preexisting SARS-CoV-2 S-specific memory B cells, as well as de novo induction, leading to production of neutralizing antibodies with enhanced potency and breadth against variants (*57*, *58*). Vaccinees receiving two doses of Ad26.COV2.S (4 months apart) had lesser Omicron immune escape than that in other two-dose vaccine recipients (3 to 4 weeks between doses) but greater than that observed in three-dose vaccinees. These findings suggest that the time interval between immunizations may affect the breadth and potency of vaccine-elicited plasma neutralizing activity and that a third dose may be beneficial for this cohort as well (*59*–*62*). Moreover, the induction by several currently available vaccines of robust cross-reactive cellular immunity against SARS-CoV-2 Omicron is likely playing a key role in the retained protection observed against severe disease (*63*, *64*).

As SARS-CoV-2 progressively becomes endemic in the human population, vaccination strategies will need to be carefully considered and optimized to provide long-lasting immunity. So far, elicitation of high titers of variant-neutralizing antibodies and protection against severe disease can be accomplished by dosing with the Wuhan-Hu-1 S antigen, as shown in animal models and studies of vaccine efficacy in humans (*5*, *48*, *65*, *66*). In fact, an Omicron BA.1 (or other variant) S boost does not offer mice or nonhuman primates significantly more BA.1 protection than a Wuhan-Hu-1 S boost (*67*–*71*), and Omicron primary infections elicit neutralizing antibody and memory responses of narrow breadth (*72*–*74*). However, continued SARS-CoV-2 evolution will accentuate the antigenic divergence from the ancestral strain, and it is unknown whether vaccines based on Wuhan-Hu-1 S alone will provide satisfactory protection, either as boosters in vaccinated or infected individuals or as an initial vaccine in naïve individuals (mainly children). The recent evaluation of intranasal vaccine administration could also be important to not only prevent severe disease but also curtail viral infection and transmission through induction of mucosal immunity (*75*–*78*). For these reasons, it is important to monitor new variants, assess the effectiveness of currently available vaccines, and continue to test and implement new vaccination strategies that may provide stronger, longer-lasting, or broader protection against SARS-CoV-2 and the entire sarbecovirus subgenus (*40*, *79*, *80*).

## Supplementary Material

20220719-1

## Supplementary Materials

### This PDF file includes:

Materials and Methods Figs. S1 to S12 Tables S1 to S4 References (*81*–*83*)

### Other Supplementary Material for this manuscript includes the following:

Movies S1 to S6

MDAR Reproducibility Checklist

## Notes

View/request a protocol for this paper from *Bio-protocol*.
