# Supplementary material for: Omicron spike function and neutralizing activity elicited by a comprehensive panel of vaccines
Source: Science. 2022 Jul 19;377(6608):890–4. doi: 10.1126/science.abq0203 (PMC9348749; doi:10.1126/science.abq0203)
Supplement: 20220719-1 [file science.abq0203.v1.pdf]

Cite as: J. E. Bowen *et al*., *Science*
10.1126/science.abq0203 (2022).

Omicron spike function and neutralizing activity elicited by a comprehensive panel of vaccines

John E. Bowen[1]†, Amin Addetia[1]†, Ha V. Dang[2], Cameron Stewart[1], Jack T. Brown[1], William K. Sharkey[1], Kaitlin R. Sprouse[1], Alexandra C. Walls[1,3], Ignacio G. Mazzitelli[4], Jennifer K. Logue[5], Nicholas M. Franko[5], Nadine Czudnochowski[2], Abigail E. Powell[2], Exequiel Dellota Jr.[2], Kumail Ahmed[6], Asefa Shariq Ansari[6], Elisabetta Cameroni[7], Andrea Gori[8,9,10], Alessandra Bandera[8,9,10], Christine M. Posavad[11], Jennifer M. Dan[12,13], Zeli Zhang[12], Daniela Weiskopf[12], Alessandro Sette[12,13], Shane Crotty[12,13], Najeeha Talat Iqbal[6], Davide Corti[7], Jorge Geffner[4], Gyorgy Snell[2], Renata Grifantini[14], Helen Y. Chu[5], David Veesler[1,3]*

[1]Department of Biochemistry, University of Washington, Seattle, WA 98195, USA. [2]Vir Biotechnology, San Francisco, CA 94158, USA. [3]Howard Hughes Medical Institute, University of Washington, Seattle, WA 98195, USA. [4]Instituto de Investigaciones Biomédicas en Retrovirus y SIDA (INBIRS), Facultad de Medicina, Buenos Aires C1121ABG, Argentina. [5]Division of Allergy and Infectious Diseases, University of Washington, Seattle, WA 98195, USA. [6]Departments of Paediatrics and Child Health and Biological and Biomedical Sciences, Aga Khan University, Karachi 74800, Pakistan. [7]Humabs Biomed SA, a subsidiary of Vir Biotechnology, 6500 Bellinzona, Switzerland. [8]Infectious Diseases Unit, Foundation IRCCS Ca' Granda Ospedale Maggiore Policlinico, Milan, Italy. [9]Department of Pathophysiology and Transplantation, University of Milan, Milan, Italy. [10]Centre for Multidisciplinary Research in Health Science (MACH), University of Milan, Milan, Italy. [11]Vaccine and Infectious Disease Division, Fred Hutchinson Cancer Research Center, Seattle, WA 98109, USA. [12]Center for Infectious Disease and Vaccine Research, La Jolla Institute for Immunology, La Jolla, CA 92037, USA. [13]Department of Medicine, Division of Infectious Diseases and Global Public Health, University of California, San Diego, La Jolla, CA 92037, USA. [14]INGM, Istituto Nazionale Genetica Molecolare "Romeo ed Enrica Invernizzi," Milan, Italy.

†These authors contributed equally to this work.

***Corresponding author. Email: dveesler@uw.edu**

The SARS-CoV-2 Omicron variant of concern comprises several sublineages with BA.2 and BA.2.12.1 having replaced the previously dominant BA.1, and BA.4 and BA.5 increasing in prevalence worldwide. We show that the large number of Omicron sublineage spike mutations lead to enhanced ACE2 binding, reduced fusogenicity, and severe dampening of plasma neutralizing activity elicited by infection or seven clinical vaccines relative to the ancestral virus. Administration of a homologous or heterologous booster based on the Wuhan-Hu-1 spike sequence markedly increased neutralizing antibody titers and breadth against BA.1, BA.2, BA.2.12.1, and BA.4/5 across all vaccines evaluated. Our data suggest that although Omicron sublineages evade polyclonal neutralizing antibody responses elicited by primary vaccine series, vaccine boosters may provide sufficient protection against Omicron-induced severe disease.

The ongoing COVID-19 pandemic has led to the emergence of SARS-CoV-2 variants with increased transmissibility, viral fitness, and immune evasion (*1*–*10*). The most recently named variant of concern, Omicron, is characterized by the greatest known genetic divergence from the ancestral virus (Wuhan-Hu-1) and consists of several sublineages including BA.1, BA.2, BA.3, BA.4, and BA.5. BA.1 was first detected in late 2021 and rapidly replaced Delta to become the globally dominant SARS-CoV-2 strain (*3*, *9*, *11*), aided by its high transmissibility and escape from neutralizing antibodies (*6*, *12*–*18*). In early March 2022, BA.2 became the most prevalent SARS-CoV-2 variant globally (*19*, *20*) (Fig. 1A) and the proportion of BA.2.12.1 in sequenced viruses peaked at over 30% worldwide and over 60% in the United States by late May 2022 (Fig. 1B). However, BA.4 and BA.5, which share the same spike (S) glycoprotein sequence, are expected to reach global dominance due to their increasing prevalence and successful replacement of BA.2 in South Africa (*21*) (Fig. 1C).

The receptor-binding domain (RBD) of the SARS-CoV-2 S glycoprotein interacts with the receptor ACE2 (*22*–*26*), promoting S conformational changes that lead to membrane fusion and viral entry (*27*–*29*). S is the main target of neutralizing antibodies, which have been shown to be a correlate of protection against SARS-CoV-2 (*30*–*38*), with RBD-targeting antibodies accounting for most neutralizing activity against vaccine-matched virus (*36*, *38*) and nearly all cross-variant neutralizing activity (*39*). SARS-CoV-2 vaccines are based on the S glycoprotein (sometimes the RBD only (*30*, *40*, *41*)) or (inactivated) virus and utilize a variety of delivery technologies. Lipid-encapsulated prefusion-stabilized S-encoding mRNA vaccines include Moderna mRNA-1273 and Pfizer/BioNTech BNT162b2. Viral-vectored vaccines encoding for the SARS-CoV-2 S sequence include Janssen Ad26.COV2.S (human adenovirus 26), AstraZeneca AZD1222 (chimpanzee adenovirus), and Gamaleya National Center of Epidemiology and Microbiology Sputnik V (human

adenovirus 26/5 for prime/boost). Novavax NVX-CoV2373 is a prefusion-stabilized S protein-subunit vaccine formulated with a saponin-based matrix M adjuvant whereas Sinopharm BBIBP-CorV comprises inactivated virions. The primary vaccine series consisted of two doses for all of these vaccines except for Ad26.COV2.S which was administered as a single dose.

We first aimed to understand how the different S mutations in the Omicron variant sublineages impact host receptor engagement and membrane fusion. Whereas the Delta RBD recognized human ACE2 with a comparable affinity to that of the Wuhan-Hu-1 RBD (1.1-fold enhancement by biolayer interferometry (BLI) (*3*) and 1.5-fold enhancement by surface plasmon resonance (SPR)), the ACE2-binding affinity was greater for the BA.1 RBD (4.4-fold by BLI and 2.6-fold by SPR) (*4*, *12*, *15*) and for the BA.2 RBD (3.7-fold by BLI, 2.3-fold by SPR) (Fig. 1, D and E; figs. S1 and S2; and tables S1 and S2). The BA.2.12.1 RBD, which differs from the BA.2 RBD only by the L452Q mutation, had an ACE2-binding affinity similar to the Wuhan-Hu-1 RBD (1.1-fold and 1.7-fold enhancements determined by BLI and SPR, respectively). The ACE2-binding affinity of the BA.4/5 RBD was the greatest among the RBDs evaluated here with 6.1-fold and 4.2-fold increases relative to Wuhan-Hu-1, as determined by BLI and SPR, respectively (Fig. 1, D and E; figs. S1 and S2; and tables S1 and S2).

We next compared the kinetics and magnitude of cell-cell fusion mediated by the Wuhan-Hu-1/G614, Delta, BA.1, BA.2, BA.2.12.2, and BA.4/5 S glycoproteins using a split GFP system (*42*) with VeroE6/TMPRSS2 target cells (expressing GFP β strands 1-10) and BHK-21 effector cells transiently transfected with S (expressing GFP β strand 11). We observed slower and markedly reduced overall fusogenicity for all tested Omicron sublineage S glycoproteins compared to Wuhan-Hu-1/G614 S and even more so relative to Delta S (*15*, *19*) (Fig. 1, F to H; fig. S3; and movies S1 to S6), despite the higher apparent BA.4/5 S cell surface expression compared to other S trimers (fig. S4).

We next assessed the plasma neutralizing activity elicited in humans by each of the seven vaccines or SARS-CoV-2 infection and evaluated the immune evasion associated with the constellation of S mutations present in the BA.1, BA.2, BA.2.12.1, and BA.4/5 Omicron sublineages (table S3). We measured entry of vesicular stomatitis virus (VSV) pseudotyped with the SARS-CoV-2 Wuhan-Hu-1 S harboring the D614G, BA.1, BA.2, BA.2.12.1, or BA.4/5 mutations into VeroE6 cells stably expressing TMPRSS2 (VeroE6/TMPRSS2) (*43*) in the presence of vaccinee or convalescent plasma (table S4). Plasma was obtained from individuals previously infected with a Washington-1-like SARS-CoV-2 strain based on time of infection. These samples were obtained early in the pandemic, so individuals had not been vaccinated. We determined a plasma neutralizing geometric mean titer (GMT) of 39 against Wuhan-Hu-1/G614 VSV S pseudovirus, and only 5/24 individuals had detectable, albeit very weak, neutralizing activity against any of the four tested Omicron sublineages (Fig. 2 and fig. S5A). Plasma from subjects that received two doses of Moderna mRNA-1273 approximately four weeks apart had Wuhan-Hu-1/G614, BA.1, BA.2, BA.2.12.1, and BA.4/5 S VSV neutralizing GMTs of 633, 33, 44, 30, and 22, respectively, whereas plasma from subjects that received two doses of Pfizer BNT162b2 approximately three weeks apart had neutralizing GMTs of 340, 20, 29, 24, and 19, respectively (Fig. 2 and fig. S5, B and C). 19/28, 21/28, 19/28, and 16/28 mRNA-vaccinated subjects retained neutralizing activity against BA.1, BA.2, BA.2.12.1, and BA.4/5 S VSV, respectively. The combined Moderna and Pfizer cohorts experienced a ≥18-fold, ≥13-fold, ≥17-fold and ≥23-fold GMT reductions against BA.1, BA.2, BA.2.12.1, and BA.4/5 S VSV, respectively. A similar trend was observed for plasma from individuals that received two doses of Novavax NVX-CoV2373 (*44*) in a double-blinded manner, although we note that these plasma samples were not obtained at peak titers due to the design of the clinical trial (figs. S6 and S7). From this group, we determined a neutralizing GMT of 252 against Wuhan-Hu-1/G614 S VSV with only 2/10 individuals having detectable neutralizing activity against BA.1 (GMT: 12, ≥22-fold drop), 7/10 against BA.2 (GMT: 15, ≥16-fold drop), 4/10 against BA.2.12.1 (GMT: 13, ≥20-fold drop), and 1/10 against BA.4/5 (GMT: 11, ≥23-fold drop) (Fig. 2 and fig. S5D). Plasma from individuals vaccinated with Janssen Ad26.COV2.S were obtained 9-142 days (mean 79) after their single dose, a timeframe expected to capture peak neutralizing titers (*45*). This resulted in a Wuhan-Hu-1/G614 S VSV GMT of 55 and only 1/12 subjects had detectable plasma neutralizing activity against any of the Omicron sublineages (Fig. 2 and fig. S5E). Two doses of AZD1222 four weeks apart induced Wuhan-Hu-1/G614, BA.1 BA.2, BA.2.12.1, and BA.4/5 S VSV neutralizing GMTs of 165, 14, 19, 15, and 14, with 13/16 and 4/16 individuals having detectable neutralizing activity against any or all tested subvariants, respectively (Fig. 2 and fig. S5F). Sputnik V vaccinee plasma after two doses had Wuhan-Hu-1/G614, BA.1, BA.2, BA.2.12.1, and BA.4/5 S VSV GMTs of 69, 13, 17, 14, and 11, respectively (Fig. 2 and fig. S5G). Detectable neutralizing activity against any or all Omicron sublineages was observed for 7/13 and 2/13 individuals, respectively. Lastly, plasma from subjects vaccinated with two doses of Sinopharm BBIBP-CorV had a neutralizing GMT against G614 S VSV of 135 with 3/12 samples retaining detectable neutralizing activity against BA.1 (GMT:14), 7/12 against BA.2 (GMT:17), 5/12 against BA.2.12.1 (GMT: 15), and 4/12 against BA.4/5 (GMT: 11) (Fig. 2 and fig. S5H). Overall, these data underscore the magnitude of evasion of polyclonal plasma neutralizing antibody responses for Omicron sublineages in humans after primary vaccine series or infection (resulting from the

accumulation of S mutations (*12*)), with a subtle but consistently more marked effect for BA.1 and even more so for BA.4/5 compared to BA.2 and BA.2.12.1.

The emergence of the SARS-CoV-2 Delta and subsequently Omicron variants of concern led to an increasing number of reinfections and vaccine breakthrough cases (*5*, *46*–*48*). Public health policies were therefore updated worldwide to recommend administration of an additional vaccine dose (booster) several months after the primary vaccine series, which has been shown to increase the breadth and potency of neutralizing antibodies (*5*, *12*, *17*, *49*). We thus assessed and compared the benefits provided by homologous or heterologous vaccine boosters on vaccinee plasma neutralizing activity against Wuhan-Hu-1/G614, BA.1, BA.2, BA.2.12.1, and BA.4/5 S VSV pseudotypes. Plasma samples of subjects that received three mRNA vaccine doses had neutralizing GMTs of 2371, 406, 448, 472, and 392 against Wuhan-Hu-1/G614, BA.1, BA.2, BA.2.12.1, and BA.4/5 S VSV, respectively (Fig. 3 and fig. S8A). The 5 to 6-fold potency losses against these variants are marked improvements over the >13 to >23-fold reductions observed after two vaccine doses, underscoring an increase in overall neutralizing antibody potency and breadth (*5*, *12*). Individuals vaccinated with two doses of NVX-CoV2373 followed by a booster of BNT162b2, mRNA-1273 or Ad26.COV2.S had neutralizing GMTs of 9298 for Wuhan-Hu-1/G614, 771 for BA.1 (12-fold reduction), 1156 for BA.2 (8-fold reduction), 1320 for BA.2.12.1 (7-fold reduction), and 575 for BA.4/5 (16-fold reduction) (Fig. 3 and fig. S8B). Plasma from individuals who received one dose of Ad26.COV2.S followed either by a homologous Ad26.COV2.S (12/14 individuals) or a heterologous BNT162b2 booster (2/14 individuals) approximately four months later had neutralizing GMTs of 363, 23, 50, 46, and 29 against Wuhan-Hu-1/G614, BA.1 BA.2, BA.2.12.1 and BA.4/5 S VSV, respectively, corresponding to dampening ranging between ≥7 to ≥16-fold with 9/14 individuals maintaining neutralizing activity against all sublineages (Fig. 3 and fig. S8C). We also investigated individuals that received two doses of AZD1222 four weeks apart followed by an mRNA booster approximately six months later. This cohort had respective neutralizing GMTs of 2167, 186, 269, 273, and 135 against Wuhan-Hu-1/G614, BA.1, BA.2, BA.2.12.1, and BA.4/5 S VSV, corresponding to 8 to 16-fold potency reductions (Fig. 3 and fig. S8D). Individuals vaccinated with two doses of Sputnik V and boosted with AZD1222 or BNT162b2 approximately nine months later had neutralizing GMTs of 509, 111, 123, 102, and 48 for Wuhan-Hu-1/G614, BA.1, BA.2, BA.2.12.1, and BA.4/5, respectively, amounting to 4 to 11-fold reductions of potency (Fig. 3 and fig. S8E). BBIBP-CorV vaccinees boosted with either BNT162b2 or mRNA-1273 had GMTs of 2047 for G614, 439 for BA.1 (5-fold reduction), 375 for BA.2 (5-fold reduction), 430 for BA.2.12.1 (5-fold reduction), and 252 for BA.4/5 (8-fold reduction) (Fig. 3 and fig. S8F). The marked improvement in plasma neutralizing activity for subjects that received a booster dose over those that did not highlights the importance of vaccine boosters for eliciting potent neutralizing antibody responses against Omicron sublineages.

To assess the impact of target cell lines on apparent Omicron immune escape, we compared the aforementioned VeroE6/TMPRSS2 cells (*43*) to a stable ACE2-overexpressing HEK293T cell line (HEK293T/ACE2) (*50*) to determine plasma neutralizing activity for a cohort of mRNA-vaccinated individuals. After primary vaccine series, only three subjects had detectable neutralizing activity against any of the tested Omicron sublineage VSV pseudotypes when using HEK293T/ACE2 target cells. In contrast, all but one subject had detectable, albeit very weak, neutralizing activity against Omicron VSV pseudotypes using VeroE6/TMPRSS2 target cells, resulting in >17-fold, >14-fold, >20-fold, and >22-fold reductions against BA.1, BA.2, BA.2.12.1, and BA.4/5, respectively (figs. S9A and S10A). Following a booster dose, we observed respective 7-fold, 7-fold, 11-fold, and 13-fold reductions of neutralizing activity against BA.1, BA.2, BA.2.12.1, and BA.4/5 VSV pseudotypes using HEK293T/ACE2 target cells, as compared to respective 7-fold, 6-fold, 5-fold, and 8-fold reductions when using VeroE6/TMPRSS2 target cells (figs. S9B and S10B). This indicates that the target cell lines used in neutralization assays may impact the observed plasma neutralizing escape of SARS-CoV-2 variants, which may be further compounded based on preferential entry routes (*15*, *23*, *51*).

Here we report that the BA.1, BA.2, BA.2.12.1, and BA.4/5 Omicron sublineages, which account for over 99% of all infections worldwide over the first half of 2022, have increased ACE2 binding affinity, decreased fusogenicity and markedly evade neutralizing antibody responses relative to the Wuhan-Hu-1 and Delta strains (*3*). Collectively, these data suggest that enhanced receptor engagement and immune evasion are key changes that may have promoted the rapid spread of these Omicron sublineages and could contribute to the current rise in prevalence of BA.4 and BA.5.

The development of life-saving vaccines is regarded as one of humanity's greatest medical and scientific achievements, which is exemplified by COVID-19 vaccines (*52*–*54*). Primary COVID-19 vaccine regimens or infection-elicited plasma neutralizing activity was severely dampened by Omicron sublineages BA.1, BA.2, BA.2.12.1, and BA.4/5. However, administration of a booster dose increased neutralizing antibody titers and breadth against all Omicron sublineages to appreciable levels regardless of the vaccine evaluated, concurring with findings for BA.1 (*5*, *12*, *15*, *17*, *49*, *55*, *56*). These results are consistent with previous studies demonstrating that a third vaccine dose results in the recall and expansion of pre-existing SARS-CoV-2 S-specific memory B cells, as well as de novo induction, leading to production of neutralizing

antibodies with enhanced potency and breadth against variants (*57*, *58*). Vaccinees receiving two doses of Ad26.COV2.S (four months apart) had lesser Omicron immune escape than other two dose vaccine recipients (three to four weeks between doses) but greater than three dose vaccinees. These findings suggest that the time interval between immunizations may affect the breadth and potency of vaccine-elicited plasma neutralizing activity, and that a third dose may be beneficial for this cohort as well (*59*–*62*). Moreover, the induction by several currently available vaccines of robust cross-reactive cellular immunity against SARS-CoV-2 Omicron is likely playing a key role in the retained protection observed against severe disease (*63*, *64*).

As SARS-CoV-2 progressively becomes endemic in the human population, vaccination strategies will need to be carefully considered and optimized to provide long-lasting immunity. So far, elicitation of high titers of variant-neutralizing antibodies and protection against severe disease can be accomplished by dosing with the Wuhan-Hu-1 S antigen, as shown in animal models and studies of vaccine efficacy in humans (*5*, *48*, *65*, *66*). In fact, an Omicron BA.1 (or other variant) S boost does not offer mice or non-human primates significantly more BA.1 protection than a Wuhan-Hu-1 S boost (*67*–*71*), and Omicron primary infections elicit neutralizing antibody and memory responses of narrow breadth (*72*–*74*). However, continued SARS-CoV-2 evolution will accentuate the antigenic divergence from the ancestral strain and it is unknown if vaccines based on Wuhan-Hu-1 S alone will provide satisfactory protection, either as boosters in vaccinated or infected individuals or as an initial vaccine in naive individuals (mainly children). The recent evaluation of intranasal vaccine administration could also be important to not only prevent severe disease but also curtail viral infection and transmission through induction of mucosal immunity (*75*–*78*). For these reasons, it is important to monitor new variants, assess the effectiveness of currently available vaccines, and continue to test and implement new vaccination strategies that may provide stronger, longer lasting, or broader protection against SARS-CoV-2 and the entire sarbecovirus subgenus (*40*, *79*, *80*).

REFERENCES AND NOTES

1. M. McCallum, J. Bassi, A. De Marco, A. Chen, A. C. Walls, J. Di Iulio, M. A. Tortorici, M.-J. Navarro, C. Silacci-Fregni, C. Saliba, K. R. Sprouse, M. Agostini, D. Pinto, K. Culap, S. Bianchi, S. Jaconi, E. Cameroni, J. E. Bowen, S. W. Tilles, M. S. Pizzuto, S. B. Guastalla, G. Bona, A. F. Pellanda, C. Garzoni, W. C. Van Voorhis, L. E. Rosen, G. Snell, A. Telenti, H. W. Virgin, L. Piccoli, D. Corti, D. Veesler, SARS-CoV-2 immune evasion by the B.1.427/B.1.429 variant of concern. *Science* **373**, 648–654 (2021). doi:10.1126/science.abi7994 Medline
2. E. B. Hodcroft, M. Zuber, S. Nadeau, T. G. Vaughan, K. H. D. Crawford, C. L. Althaus, M. L. Reichmuth, J. E. Bowen, A. C. Walls, D. Corti, J. D. Bloom, D. Veesler, D. Mateo, A. Hernando, I. Comas, F. González-Candelas, SeqCOVID-SPAIN consortium, T. Stadler, R. A. Neher, Spread of a SARS-CoV-2 variant through Europe in the summer of 2020. *Nature* **595**, 707–712 (2021). doi:10.1038/s41586-021-03677-y Medline
3. M. McCallum, A. C. Walls, K. R. Sprouse, J. E. Bowen, L. E. Rosen, H. V. Dang, A. De Marco, N. Franko, S. W. Tilles, J. Logue, M. C. Miranda, M. Ahlrichs, L. Carter, G. Snell, M. S. Pizzuto, H. Y. Chu, W. C. Van Voorhis, D. Corti, D. Veesler, Molecular basis of immune evasion by the Delta and Kappa SARS-CoV-2 variants. *Science* **374**, 1621–1626 (2021). doi:10.1126/science.abl8506 Medline
4. M. McCallum, N. Czudnochowski, L. E. Rosen, S. K. Zepeda, J. E. Bowen, A. C. Walls, K. Hauser, A. Joshi, C. Stewart, J. R. Dillen, A. E. Powell, T. I. Croll, J. Nix, H. W. Virgin, D. Corti, G. Snell, D. Veesler, Structural basis of SARS-CoV-2 Omicron immune evasion and receptor engagement. *Science* **375**, 864–868 (2022). doi:10.1126/science.abn8652 Medline
5. A. C. Walls, K. R. Sprouse, J. E. Bowen, A. Joshi, N. Franko, M. J. Navarro, C. Stewart, E. Cameroni, M. McCallum, E. A. Goecker, E. J. Degli-Angeli, J. Logue, A. Greninger, D. Corti, H. Y. Chu, D. Veesler, SARS-CoV-2 breakthrough infections elicit potent, broad, and durable neutralizing antibody responses. *Cell* **185**, 872–880.e3 (2022). doi:10.1016/j.cell.2022.01.011 Medline
6. S. Cele, L. Jackson, D. S. Khoury, K. Khan, T. Moyo-Gwete, H. Tegally, J. E. San, D. Cromer, C. Scheepers, D. G. Amoako, F. Karim, M. Bernstein, G. Lustig, D. Archary, M. Smith, Y. Ganga, Z. Jule, K. Reedoy, S.-H. Hwa, J. Giandhari, J. M. Blackburn, B. I. Gosnell, S. S. Abdool Karim, W. Hanekom, NGS-SA, COMMIT-KZN Team, A. von Gottberg, J. N. Bhiman, R. J. Lessells, M.-Y. S. Moosa, M. P. Davenport, T. de Oliveira, P. L. Moore, A. Sigal, Omicron extensively but incompletely escapes Pfizer BNT162b2 neutralization. *Nature* **602**, 654–656 (2022). doi:10.1038/s41586-021-04387-1 Medline
7. R. Viana, S. Moyo, D. G. Amoako, H. Tegally, C. Scheepers, C. L. Althaus, U. J. Anyaneji, P. A. Bester, M. F. Boni, M. Chand, W. T. Choga, R. Colquhoun, M. Davids, K. Deforche, D. Doolabh, L. du Plessis, S. Engelbrecht, J. Everatt, J. Giandhari, M. Giovanetti, D. Hardie, V. Hill, N.-Y. Hsiao, A. Iranzadeh, A. Ismail, C. Joseph, R. Joseph, L. Koopile, S. L. Kosakovsky Pond, M. U. G. Kraemer, L. Kuate-Lere, O. Laguda-Akingba, O. Lesetedi-Mafoko, R. J. Lessells, S. Lockman, A. G. Lucaci, A. Maharaj, B. Mahlangu, T. Maponga, K. Mahlakwane, Z. Makatini, G. Marais, D. Maruapula, K. Masupu, M. Matshaba, S. Mayaphi, N. Mbhele, M. B. Mbulawa, A. Mendes, K. Mlisana, A. Mnguni, T. Mohale, M. Moir, K. Moruisi, M. Mosepele, G. Motsatsi, M. S. Motswaledi, T. Mphoyakgosi, N. Msomi, P. N. Mwangi, Y. Naidoo, N. Ntuli, M. Nyaga, L. Olubayo, S. Pillay, B. Radibe, Y. Ramphal, U. Ramphal, J. E. San, L. Scott, R. Shapiro, L. Singh, P. Smith-Lawrence, W. Stevens, A. Strydom, K. Subramoney, N. Tebeila, D. Tshiabuila, J. Tsui, S. van Wyk, S. Weaver, C. K. Wibmer, E. Wilkinson, N. Wolter, A. E. Zarebski, B. Zuze, D. Goedhals, W. Preiser, F. Treurnicht, M. Venter, C. Williamson, O. G. Pybus, J. Bhiman, A. Glass, D. P. Martin, A. Rambaut, S. Gaseitsiwe, A. von Gottberg, T. de Oliveira, Rapid epidemic expansion of the SARS-CoV-2 Omicron variant in southern Africa. *Nature* **603**, 679–686 (2022). doi:10.1038/s41586-022-04411-y Medline
8. H. Tegally, E. Wilkinson, M. Giovanetti, A. Iranzadeh, V. Fonseca, J. Giandhari, D. Doolabh, S. Pillay, E. J. San, N. Msomi, K. Mlisana, A. von Gottberg, S. Walaza, M. Allam, A. Ismail, T. Mohale, A. J. Glass, S. Engelbrecht, G. Van Zyl, W. Preiser, F. Petruccione, A. Sigal, D. Hardie, G. Marais, N. Hsiao, S. Korsman, M.-A. Davies, L. Tyers, I. Mudau, D. York, C. Maslo, D. Goedhals, S. Abrahams, O. Laguda-Akingba, A. Alisoltani-Dehkordi, A. Godzik, C. K. Wibmer, B. T. Sewell, J. Lourenço, L. C. J. Alcantara, S. L. Kosakovsky Pond, S. Weaver, D. Martin, R. J. Lessells, J. N. Bhiman, C. Williamson, T. de Oliveira, Detection of a SARS-CoV-2 variant of concern in South Africa. *Nature* **592**, 438–443 (2021). doi:10.1038/s41586-021-03402-9 Medline
9. P. Mlcochova, S. A. Kemp, M. S. Dhar, G. Papa, B. Meng, I. A. T. M. Ferreira, R. Datir, D. A. Collier, A. Albecka, S. Singh, R. Pandey, J. Brown, J. Zhou, N. Goonawardane, S. Mishra, C. Whittaker, T. Mellan, R. Marwal, M. Datta, S. Sengupta, K. Ponnusamy, V. S. Radhakrishnan, A. Abdullahi, O. Charles, P. Chattopadhyay, P. Devi, D. Caputo, T. Peacock, C. Wattal, N. Goel, A. Satwik, R. Vaishya, M. Agarwal, Indian SARS-CoV-2 Genomics Consortium (INSACOG), Genotype to Phenotype Japan (G2P-Japan) Consortium, CITIID-NIHR BioResource COVID-19 Collaboration, A. Mavousian, J. H. Lee, J. Bassi, C. Silacci-Fegni, C. Saliba, D. Pinto, T. Irie, I. Yoshida, W. L. Hamilton, K. Sato, S. Bhatt, S. Flaxman, L. C. James, D. Corti, L. Piccoli, W. S. Barclay, P. Rakshit, A. Agrawal, R. K. Gupta, SARS-CoV-2 B.1.617.2 Delta variant replication and immune evasion. *Nature* **599**, 114–119 (2021). doi:10.1038/s41586-021-03944-y Medline
10. D. A. Collier, A. De Marco, I. A. T. M. Ferreira, B. Meng, R. P. Datir, A. C. Walls, S. A. Kemp, J. Bassi, D. Pinto, C. Silacci-Fregni, S. Bianchi, M. A. Tortorici, J. Bowen, K. Culap, S. Jaconi, E. Cameroni, G. Snell, M. S. Pizzuto, A. F. Pellanda, C. Garzoni, A.

Riva, CITIID-NIHR BioResource COVID-19 Collaboration, A. Elmer, N. Kingston, B. Graves, L. E. McCoy, K. G. C. Smith, J. R. Bradley, N. Temperton, L. Ceron-Gutierrez, G. Barcenas-Morales, COVID-19 Genomics UK (COG-UK) Consortium, W. Harvey, H. W. Virgin, A. Lanzavecchia, L. Piccoli, R. Doffinger, M. Wills, D. Veesler, D. Corti, R. K. Gupta, Sensitivity of SARS-CoV-2 B.1.1.7 to mRNA vaccine-elicited antibodies. *Nature* **593**, 136–141 (2021). doi:10.1038/s41586-021-03412-7 Medline

11. A. Saito, T. Irie, R. Suzuki, T. Maemura, H. Nasser, K. Uriu, Y. Kosugi, K. Shirakawa, K. Sadamasu, I. Kimura, J. Ito, J. Wu, K. Iwatsuki-Horimoto, M. Ito, S. Yamayoshi, S. Loeber, M. Tsuda, L. Wang, S. Ozono, E. P. Butlertanaka, Y. L. Tanaka, R. Shimizu, K. Shimizu, K. Yoshimatsu, R. Kawabata, T. Sakaguchi, K. Tokunaga, I. Yoshida, H. Asakura, M. Nagashima, Y. Kazuma, R. Nomura, Y. Horisawa, K. Yoshimura, A. Takaori-Kondo, M. Imai, Genotype to Phenotype Japan (G2P-Japan) Consortium, S. Tanaka, S. Nakagawa, T. Ikeda, T. Fukuhara, Y. Kawaoka, K. Sato, Enhanced fusogenicity and pathogenicity of SARS-CoV-2 Delta P681R mutation. *Nature* **602**, 300–306 (2022). doi:10.1038/s41586-021-04266-9 Medline
12. E. Cameroni, J. E. Bowen, L. E. Rosen, C. Saliba, S. K. Zepeda, K. Culap, D. Pinto, L. A. VanBlargan, A. De Marco, J. di Iulio, F. Zatta, H. Kaiser, J. Noack, N. Farhat, N. Czudnochowski, C. Havenar-Daughton, K. R. Sprouse, J. R. Dillen, A. E. Powell, A. Chen, C. Maher, L. Yin, D. Sun, L. Soriaga, J. Bassi, C. Silacci-Fregni, C. Gustafsson, N. M. Franko, J. Logue, N. T. Iqbal, I. Mazzitelli, J. Geffner, R. Grifantini, H. Chu, A. Gori, A. Riva, O. Giannini, A. Ceschi, P. Ferrari, P. E. Cippà, A. Franzetti-Pellanda, C. Garzoni, P. J. Halfmann, Y. Kawaoka, C. Hebner, L. A. Purcell, L. Piccoli, M. S. Pizzuto, A. C. Walls, M. S. Diamond, A. Telenti, H. W. Virgin, A. Lanzavecchia, G. Snell, D. Veesler, D. Corti, Broadly neutralizing antibodies overcome SARS-CoV-2 Omicron antigenic shift. *Nature* **602**, 664–670 (2022). doi:10.1038/s41586-021-04386-2 Medline
13. F. Schmidt, F. Muecksch, Y. Weisblum, J. Da Silva, E. Bednarski, A. Cho, Z. Wang, C. Gaebler, M. Caskey, M. C. Nussenzweig, T. Hatziioannou, P. D. Bieniasz, Plasma Neutralization of the SARS-CoV-2 Omicron Variant. *N. Engl. J. Med.* **386**, 599–601 (2022). doi:10.1056/NEJMc2119641 Medline
14. A. Wilhelm, M. Widera, K. Grikscheit, T. Toptan, B. Schenk, C. Pallas, M. Metzler, N. Kohmer, S. Hoehl, F. A. Helfritz, T. Wolf, U. Goetsch, S. Ciesek, Reduced neutralization of SARS-CoV-2 Omicron variant by vaccine Sera and monoclonal antibodies. medRxiv 2021.12.07.21267432 [Preprint] (2021). https://doi.org/10.1101/2021.12.07.21267432.
15. B. Meng, A. Abdullahi, I. A. T. M. Ferreira, N. Goonawardane, A. Saito, I. Kimura, D. Yamasoba, P. P. Gerber, S. Fatihi, S. Rathore, S. K. Zepeda, G. Papa, S. A. Kemp, T. Ikeda, M. Toyoda, T. S. Tan, J. Kuramochi, S. Mitsunaga, T. Ueno, K. Shirakawa, A. Takaori-Kondo, T. Brevini, D. L. Mallery, O. J. Charles, CITIID-NIHR BioResource COVID-19 Collaboration, Genotype to Phenotype Japan (GP-Japan) Consortium, Ecuador-COVID19 Consortium, J. E. Bowen, A. Joshi, A. C. Walls, L. Jackson, D. Martin, K. G. C. Smith, J. Bradley, J. A. G. Briggs, J. Choi, E. Madissoon, K. B. Meyer, P. Mlcochova, L. Ceron-Gutierrez, R. Doffinger, S. A. Teichmann, A. J. Fisher, M. S. Pizzuto, A. de Marco, D. Corti, M. Hosmillo, J. H. Lee, L. C. James, L. Thukral, D. Veesler, A. Sigal, F. Sampaziotis, I. G. Goodfellow, N. J. Matheson, K. Sato, R. K. Gupta, Altered TMPRSS2 usage by SARS-CoV-2 Omicron impacts infectivity and fusogenicity. *Nature* **603**, 706–714 (2022). doi:10.1038/s41586-022-04474-x Medline
16. H. Allen, E. Tessier, C. Turner, C. Anderson, P. Blomquist, D. Simons, A. Løchen, C. I. Jarvis, N. Groves, F. Capelastegui, J. Flannagan, A. Zaidi, C. Chen, C. Rawlinson, G. J. Hughes, D. Chudasama, S. Nash, S. Thelwall, J. Lopez-Bernal, G. Dabrera, A. Charlett, M. Kall, T. Lamagni, Comparative transmission of SARS-CoV-2 Omicron (B.1.1.529) and Delta (B.1.617.2) variants and the impact of vaccination: National cohort study, England. medRxiv 2022.02.15.22271001 [Preprint] (2022). https://doi.org/10.1101/2022.02.15.22271001.
17. W. Dejnirattisai, J. Huo, D. Zhou, J. Zahradník, P. Supasa, C. Liu, H. M. E. Duyvesteyn, H. M. Ginn, A. J. Mentzer, A. Tuekprakhon, R. Nutalai, B. Wang, A. Dijokaite, S. Khan, O. Avinoam, M. Bahar, D. Skelly, S. Adele, S. A. Johnson, A. Amini, T. G. Ritter, C. Mason, C. Dold, D. Pan, S. Assadi, A. Bellass, N. Omo-Dare, D. Koeckerling, A. Flaxman, D. Jenkin, P. K. Aley, M. Voysey, S. A. Costa Clemens, F. G. Naveca, V. Nascimento, F. Nascimento, C. F. da Costa, P. C. Resende, A. Pauvolid-Correa, M. M. Siqueira, V. Baillie, N. Serafin, G. Kwatra, K. Da Silva, S. A. Madhi, M. C. Nunes, T. Malik, P. J. M. Openshaw, J. K. Baillie, M. G. Semple, A. R. Townsend, K.-Y. A. Huang, T. K. Tan, M. W. Carroll, P. Klenerman, E. Barnes, S. J. Dunachie, B. Constantinides, H. Webster, D. Crook, A. J. Pollard, T. Lambe, OPTIC Consortium, ISARIC4C Consortium, N. G. Paterson, M. A. Williams, D. R. Hall, E. E. Fry, J. Mongkolsapaya, J. Ren, G. Schreiber, D. I. Stuart, G. R. Screaton, SARS-CoV-2 Omicron-B.1.1.529 leads to widespread escape from neutralizing antibody responses. *Cell* **185**, 467–484.e15 (2022). doi:10.1016/j.cell.2021.12.046 Medline
18. T. N. Starr, A. J. Greaney, W. W. Hannon, A. N. Loes, K. Hauser, J. R. Dillen, E. Ferri, A. G. Farrell, B. Dadonaite, M. McCallum, K. A. Matreyek, D. Corti, D. Veesler, G. Snell, J. D. Bloom, Shifting mutational constraints in the SARS-CoV-2 receptor-binding domain during viral evolution. *Science* 10.1126/science.abo7896 (2022). doi:10.1126/science.abo7896 Medline
19. R. Suzuki, D. Yamasoba, I. Kimura, L. Wang, M. Kishimoto, J. Ito, Y. Morioka, N. Nao, H. Nasser, K. Uriu, Y. Kosugi, M. Tsuda, Y. Orba, M. Sasaki, R. Shimizu, R. Kawabata, K. Yoshimatsu, H. Asakura, M. Nagashima, K. Sadamasu, K. Yoshimura, Genotype to Phenotype Japan (G2P-Japan) Consortium, H. Sawa, T. Ikeda, T. Irie, K. Matsuno, S. Tanaka, T. Fukuhara, K. Sato, Attenuated fusogenicity and pathogenicity of SARS-CoV-2 Omicron variant. *Nature* **603**, 700–705 (2022). doi:10.1038/s41586-022-04462-1 Medline
20. D. Yamasoba, I. Kimura, H. Nasser, Y. Morioka, N. Nao, J. Ito, K. Uriu, M. Tsuda, J. Zahradnik, K. Shirakawa, R. Suzuki, M. Kishimoto, Y. Kosugi, K. Kobiyama, T. Hara, M. Toyoda, Y. L. Tanaka, E. P. Butlertanaka, R. Shimizu, H. Ito, L. Wang, Y. Oda, Y. Orba, M. Sasaki, K. Nagata, K. Yoshimatsu, H. Asakura, M. Nagashima, K. Sadamasu, K. Yoshimura, J. Kuramochi, M. Seki, R. Fujiki, A. Kaneda, T. Shimada, T.-a. Nakada, S. Sakao, T. Suzuki, T. Ueno, A. Takaori-Kondo, K. J. Ishii, G. Schreiber, Genotype to Phenotype Japan (G2P-Japan) Consortium, H. Sawa, A. Saito, T. Irie, S. Tanaka, K. Matsuno, T. Fukuhara, T. Ikeda, K. Sato, Virological characteristics of the SARS-CoV-2 Omicron BA.2 spike. *Cell* **185**, 2103–2115.e19 (2022). doi:10.1016/j.cell.2022.04.035 Medline
21. H. Tegally, M. Moir, J. Everatt, M. Giovanetti, C. Scheepers, E. Wilkinson, K. Subramoney, S. Moyo, D. G. Amoako, C. Baxter, C. L. Althaus, U. J. Anyaneji, D. Kekana, R. Viana, J. Giandhari, R. J. Lessells, T. Maponga, D. Maruapula, W. Choga, M. Matshaba, S. Mayaphi, N. Mbhele, M. B. Mbulawa, N. Msomi, NGS-SA consortium, Y. Naidoo, S. Pillay, T. J. Sanko, J. E. San, L. Scott, L. Singh, N. A. Magini, P. Smith-Lawrence, W. Stevens, G. Dor, D. Tshiabuila, N. Wolter, W. Preiser, F. K. Treurnicht, M. Venter, M. Davids, G. Chiloane, A. Mendes, C. McIntyre, A. O'Toole, C. Ruis, T. P. Peacock, C. Roemer, C. Williamson, O. G. Pybus, J. Bhiman, A. Glass, D. P. Martin, A. Rambaut, S. Gaseitsiwe, A. von Gottberg, T. de Oliveira, Continued emergence and evolution of Omicron in South Africa: New BA.4 and BA.5 lineages. medRxiv 2022.05.01.22274406 [Preprint] (2022). https://doi.org/10.1101/2022.05.01.22274406.
22. A. C. Walls, Y.-J. Park, M. A. Tortorici, A. Wall, A. T. McGuire, D. Veesler, Structure, Function, and Antigenicity of the SARS-CoV-2 Spike Glycoprotein. *Cell* **181**, 281–292.e6 (2020). doi:10.1016/j.cell.2020.02.058 Medline
23. M. Hoffmann, H. Kleine-Weber, S. Schroeder, N. Krüger, T. Herrler, S. Erichsen, T. S. Schiergens, G. Herrler, N.-H. Wu, A. Nitsche, M. A. Müller, C. Drosten, S. Pöhlmann, SARS-CoV-2 Cell Entry Depends on ACE2 and TMPRSS2 and Is Blocked by a Clinically Proven Protease Inhibitor. *Cell* **181**, 271–280.e8 (2020). doi:10.1016/j.cell.2020.02.052 Medline
24. P. Zhou, X.-L. Yang, X.-G. Wang, B. Hu, L. Zhang, W. Zhang, H.-R. Si, Y. Zhu, B. Li, C.-L. Huang, H.-D. Chen, J. Chen, Y. Luo, H. Guo, R.-D. Jiang, M.-Q. Liu, Y. Chen, X.-R. Shen, X. Wang, X.-S. Zheng, K. Zhao, Q.-J. Chen, F. Deng, L.-L. Liu, B. Yan, F.-X. Zhan, Y.-Y. Wang, G.-F. Xiao, Z.-L. Shi, A pneumonia outbreak associated with a new coronavirus of probable bat origin. *Nature* **579**, 270–273 (2020). doi:10.1038/s41586-020-2012-7 Medline
25. M. Letko, A. Marzi, V. Munster, Functional assessment of cell entry and receptor usage for SARS-CoV-2 and other lineage B betacoronaviruses. *Nat. Microbiol.* **5**, 562–569 (2020). doi:10.1038/s41564-020-0688-y Medline
26. D. Wrapp, N. Wang, K. S. Corbett, J. A. Goldsmith, C.-L. Hsieh, O. Abiona, B. S. Graham, J. S. McLellan, Cryo-EM structure of the 2019-nCoV spike in the prefusion conformation. *Science* **367**, 1260–1263 (2020). doi:10.1126/science.abb2507 Medline
27. A. C. Walls, M. A. Tortorici, J. Snijder, X. Xiong, B.-J. Bosch, F. A. Rey, D. Veesler, Tectonic conformational changes of a coronavirus spike glycoprotein promote membrane fusion. *Proc. Natl. Acad. Sci. U.S.A.* **114**, 11157–11162 (2017). doi:10.1073/pnas.1708727114 Medline

28. Y. Cai, J. Zhang, T. Xiao, H. Peng, S. M. Sterling, R. M. Walsh Jr., S. Rawson, S. Rits-Volloch, B. Chen, Distinct conformational states of SARS-CoV-2 spike protein. *Science* **369**, 1586–1592 (2020). doi:10.1126/science.abd4251 Medline
29. A. C. Walls, X. Xiong, Y. J. Park, M. A. Tortorici, J. Snijder, J. Quispe, E. Cameroni, R. Gopal, M. Dai, A. Lanzavecchia, M. Zambon, F. A. Rey, D. Corti, D. Veesler, Unexpected Receptor Functional Mimicry Elucidates Activation of Coronavirus Fusion. *Cell* **176**, 1026–1039.e15 (2019). doi:10.1016/j.cell.2018.12.028 Medline
30. P. S. Arunachalam, A. C. Walls, N. Golden, C. Atyeo, S. Fischinger, C. Li, P. Aye, M. J. Navarro, L. Lai, V. V. Edara, K. Röltgen, K. Rogers, L. Shirreff, D. E. Ferrell, S. Wrenn, D. Pettie, J. C. Kraft, M. C. Miranda, E. Kepl, C. Sydeman, N. Brunette, M. Murphy, B. Fiala, L. Carter, A. G. White, M. Trisal, C.-L. Hsieh, K. Russell-Lodrigue, C. Monjure, J. Dufour, S. Spencer, L. Doyle-Meyers, R. P. Bohm, N. J. Maness, C. Roy, J. A. Plante, K. S. Plante, A. Zhu, M. J. Gorman, S. Shin, X. Shen, J. Fontenot, S. Gupta, D. T. O'Hagan, R. Van Der Most, R. Rappuoli, R. L. Coffman, D. Novack, J. S. McLellan, S. Subramaniam, D. Montefiori, S. D. Boyd, J. L. Flynn, G. Alter, F. Villinger, H. Kleanthous, J. Rappaport, M. S. Suthar, N. P. King, D. Veesler, B. Pulendran, Adjuvanting a subunit COVID-19 vaccine to induce protective immunity. *Nature* **594**, 253–258 (2021). doi:10.1038/s41586-021-03530-2 Medline
31. K. McMahan, J. Yu, N. B. Mercado, C. Loos, L. H. Tostanoski, A. Chandrashekar, J. Liu, L. Peter, C. Atyeo, A. Zhu, E. A. Bondzie, G. Dagotto, M. S. Gebre, C. Jacob-Dolan, Z. Li, F. Nampanya, S. Patel, L. Pessaint, A. Van Ry, K. Blade, J. Yalley-Ogunro, M. Cabus, R. Brown, A. Cook, E. Teow, H. Andersen, M. G. Lewis, D. A. Lauffenburger, G. Alter, D. H. Barouch, Correlates of protection against SARS-CoV-2 in rhesus macaques. *Nature* **590**, 630–634 (2021). doi:10.1038/s41586-020-03041-6 Medline
32. D. S. Khoury, D. Cromer, A. Reynaldi, T. E. Schlub, A. K. Wheatley, J. A. Juno, K. Subbarao, S. J. Kent, J. A. Triccas, M. P. Davenport, Neutralizing antibody levels are highly predictive of immune protection from symptomatic SARS-CoV-2 infection. *Nat. Med.* **27**, 1205–1211 (2021). doi:10.1038/s41591-021-01377-8 Medline
33. K. S. Corbett, M. C. Nason, B. Flach, M. Gagne, S. O'Connell, T. S. Johnston, S. N. Shah, V. V. Edara, K. Floyd, L. Lai, C. McDanal, J. R. Francica, B. Flynn, K. Wu, A. Choi, M. Koch, O. M. Abiona, A. P. Werner, J. I. Moliva, S. F. Andrew, M. M. Donaldson, J. Fintzi, D. R. Flebbe, E. Lamb, A. T. Noe, S. T. Nurmukhambetova, S. J. Provost, A. Cook, A. Dodson, A. Faudree, J. Greenhouse, S. Kar, L. Pessaint, M. Porto, K. Steingrebe, D. Valentin, S. Zouantcha, K. W. Bock, M. Minai, B. M. Nagata, R. van de Wetering, S. Boyoglu-Barnum, K. Leung, W. Shi, E. S. Yang, Y. Zhang, J. M. Todd, L. Wang, G. S. Alvarado, H. Andersen, K. E. Foulds, D. K. Edwards, J. R. Mascola, I. N. Moore, M. G. Lewis, A. Carfi, D. Montefiori, M. S. Suthar, A. McDermott, M. Roederer, N. J. Sullivan, D. C. Douek, B. S. Graham, R. A. Seder, Immune correlates of protection by mRNA-1273 vaccine against SARS-CoV-2 in nonhuman primates. *Science* **373**, eabj0299 (2021). doi:10.1126/science.abj0299 Medline
34. P. B. Gilbert, D. C. Montefiori, A. B. McDermott, Y. Fong, D. Benkeser, W. Deng, H. Zhou, C. R. Houchens, K. Martins, L. Jayashankar, F. Castellino, B. Flach, B. C. Lin, S. O'Connell, C. McDanal, A. Eaton, M. Sarzotti-Kelsoe, Y. Lu, C. Yu, B. Borate, L. W. P. van der Laan, N. S. Hejazi, C. Huynh, J. Miller, H. M. El Sahly, L. R. Baden, M. Baron, L. De La Cruz, C. Gay, S. Kalams, C. F. Kelley, M. P. Andrasik, J. G. Kublin, L. Corey, K. M. Neuzil, L. N. Carpp, R. Pajon, D. Follmann, R. O. Donis, R. A. Koup, Immune Assays Team, Moderna, Inc. Team, Coronavirus Vaccine Prevention Network (CoVPN)/Coronavirus Efficacy (COVE) Team, United States Government (USG)/CoVPN Biostatistics Team, Immune correlates analysis of the mRNA-1273 COVID-19 vaccine efficacy clinical trial. *Science* **375**, 43–50 (2022). doi:10.1126/science.abm3425 Medline
35. D. Corti, L. A. Purcell, G. Snell, D. Veesler, Tackling COVID-19 with neutralizing monoclonal antibodies. *Cell* **184**, 3086–3108 (2021). doi:10.1016/j.cell.2021.05.005 Medline
36. L. Piccoli, Y. J. Park, M. A. Tortorici, N. Czudnochowski, A. C. Walls, M. Beltramello, C. Silacci-Fregni, D. Pinto, L. E. Rosen, J. E. Bowen, O. J. Acton, S. Jaconi, B. Guarino, A. Minola, F. Zatta, N. Sprugasci, J. Bassi, A. Peter, A. De Marco, J. C. Nix, F. Mele, S. Jovic, B. F. Rodriguez, S. V. Gupta, F. Jin, G. Piumatti, G. Lo Presti, A. F. Pellanda, M. Biggiogero, M. Tarkowski, M. S. Pizzuto, E. Cameroni, C. Havenar-Daughton, M. Smithey, D. Hong, V. Lepori, E. Albanese, A. Ceschi, E. Bernasconi, L. Elzi, P. Ferrari, C. Garzoni, A. Riva, G. Snell, F. Sallusto, K. Fink, H. W. Virgin, A. Lanzavecchia, D. Corti, D. Veesler, Mapping Neutralizing and Immunodominant Sites on the SARS-CoV-2 Spike Receptor-Binding Domain by Structure-Guided High-Resolution Serology. *Cell* **183**, 1024–1042.e21 (2020). doi:10.1016/j.cell.2020.09.037 Medline
37. L. Stamatatos, J. Czartoski, Y.-H. Wan, L. J. Homad, V. Rubin, H. Glantz, M. Neradilek, E. Seydoux, M. F. Jennewein, A. J. MacCamy, J. Feng, G. Mize, S. C. De Rosa, A. Finzi, M. P. Lemos, K. W. Cohen, Z. Moodie, M. J. McElrath, A. T. McGuire, mRNA vaccination boosts cross-variant neutralizing antibodies elicited by SARS-CoV-2 infection. *Science* **372**, 1413–1418 (2021). doi:10.1126/science.abg9175 Medline
38. A. J. Greaney, A. N. Loes, L. E. Gentles, K. H. D. Crawford, T. N. Starr, K. D. Malone, H. Y. Chu, J. D. Bloom, Antibodies elicited by mRNA-1273 vaccination bind more broadly to the receptor binding domain than do those from SARS-CoV-2 infection. *Sci. Transl. Med.* **13**, eabi9915 (2021). doi:10.1126/scitranslmed.abi9915 Medline
39. J. E. Bowen, A. C. Walls, A. Joshi, K. R. Sprouse, C. Stewart, M. A. Tortorici, N. M. Franko, J. K. Logue, I. G. Mazzitelli, S. W. Tiles, K. Ahmed, A. Shariq, G. Snell, N. T. Iqbal, J. Geffner, A. Bandera, A. Gori, R. Grifantini, H. Y. Chu, W. C. Van Voorhis, D. Corti, D. Veesler, SARS-CoV-2 spike conformation determines plasma neutralizing activity. bioRxiv 2021.12.19.473391 [Preprint] (2021). https://doi.org/10.1101/2021.12.19.473391.
40. A. C. Walls, M. C. Miranda, A. Schäfer, M. N. Pham, A. Greaney, P. S. Arunachalam, M.-J. Navarro, M. A. Tortorici, K. Rogers, M. A. O'Connor, L. Shirreff, D. E. Ferrell, J. Bowen, N. Brunette, E. Kepl, S. K. Zepeda, T. Starr, C.-L. Hsieh, B. Fiala, S. Wrenn, D. Pettie, C. Sydeman, K. R. Sprouse, M. Johnson, A. Blackstone, R. Ravichandran, C. Ogohara, L. Carter, S. W. Tilles, R. Rappuoli, S. R. Leist, D. R. Martinez, M. Clark, R. Tisch, D. T. O'Hagan, R. Van Der Most, W. C. Van Voorhis, D. Corti, J. S. McLellan, H. Kleanthous, T. P. Sheahan, K. D. Smith, D. H. Fuller, F. Villinger, J. Bloom, B. Pulendran, R. S. Baric, N. P. King, D. Veesler, Elicitation of broadly protective sarbecovirus immunity by receptor-binding domain nanoparticle vaccines. *Cell* **184**, 5432–5447.e16 (2021). doi:10.1016/j.cell.2021.09.015 Medline
41. A. C. Walls, B. Fiala, A. Schäfer, S. Wrenn, M. N. Pham, M. Murphy, L. V. Tse, L. Shehata, M. A. O'Connor, C. Chen, M. J. Navarro, M. C. Miranda, D. Pettie, R. Ravichandran, J. C. Kraft, C. Ogohara, A. Palser, S. Chalk, E. C. Lee, K. Guerriero, E. Kepl, C. M. Chow, C. Sydeman, E. A. Hodge, B. Brown, J. T. Fuller, K. H. Dinnon 3rd, L. E. Gralinski, S. R. Leist, K. L. Gully, T. B. Lewis, M. Guttman, H. Y. Chu, K. K. Lee, D. H. Fuller, R. S. Baric, P. Kellam, L. Carter, M. Pepper, T. P. Sheahan, D. Veesler, N. P. King, Elicitation of Potent Neutralizing Antibody Responses by Designed Protein Nanoparticle Vaccines for SARS-CoV-2. *Cell* **183**, 1367–1382.e17 (2020). doi:10.1016/j.cell.2020.10.043 Medline
42. M. Kodaka, Z. Yang, K. Nakagawa, J. Maruyama, X. Xu, A. Sarkar, A. Ichimura, Y. Nasu, T. Ozawa, H. Iwasa, M. Ishigami-Yuasa, S. Ito, H. Kagechika, Y. Hata, A new cell-based assay to evaluate myogenesis in mouse myoblast C2C12 cells. *Exp. Cell Res.* **336**, 171–181 (2015). doi:10.1016/j.yexcr.2015.06.015 Medline
43. F. A. Lempp, L. B. Soriaga, M. Montiel-Ruiz, F. Benigni, J. Noack, Y.-J. Park, S. Bianchi, A. C. Walls, J. E. Bowen, J. Zhou, H. Kaiser, A. Joshi, M. Agostini, M. Meury, E. Dellota Jr., S. Jaconi, E. Cameroni, J. Martinez-Picado, J. Vergara-Alert, N. Izquierdo-Useros, H. W. Virgin, A. Lanzavecchia, D. Veesler, L. A. Purcell, A. Telenti, D. Corti, Lectins enhance SARS-CoV-2 infection and influence neutralizing antibodies. *Nature* **598**, 342–347 (2021). doi:10.1038/s41586-021-03925-1 Medline
44. Z. Zhang, J. Mateus, C. H. Coelho, J. M. Dan, C. R. Moderbacher, R. I. Gálvez, F. H. Cortes, A. Grifoni, A. Tarke, J. Chang, E. A. Escarrega, C. Kim, B. Goodwin, N. I. Bloom, A. Frazier, D. Weiskopf, A. Sette, S. Crotty, Humoral and cellular immune memory to four COVID-19 vaccines. *Cell* **185**, 2434–2451.e17 (2022). doi:10.1016/j.cell.2022.05.022
45. D. H. Barouch, K. E. Stephenson, J. Sadoff, J. Yu, A. Chang, M. Gebre, K. McMahan, J. Liu, A. Chandrashekar, S. Patel, M. Le Gars, A. M. de Groot, D. Heerwegh, F. Struyf, M. Douoguih, J. van Hoof, H. Schuitemaker, Durable Humoral and Cellular Immune Responses 8 Months after Ad26.COV2.S Vaccination. *N. Engl. J. Med.* **385**, 951–953 (2021). doi:10.1056/NEJMc2108829 Medline
46. T. A. Bates, S. K. McBride, B. Winders, D. Schoen, L. Trautmann, M. E. Curlin, F. G. Tafesse, Antibody Response and Variant Cross-Neutralization After SARS-CoV-2 Breakthrough Infection. *JAMA* **327**, 179–181 (2022). doi:10.1001/jama.2021.22898 Medline

47. A.-r. Y. Collier, C. M. Brown, K. A. McMahan, J. Yu, J. Liu, C. Jacob-Dolan, A. Chandrashekar, D. Tierney, J. L. Ansel, M. Rowe, D. Sellers, K. Ahmad, R. Aguayo, T. Anioke, S. Gardner, M. Siamatu, L. Bermudez-Rivera, M. R. Hacker, L. C. Madoff, D. H. Barouch, Characterization of immune responses in fully vaccinated individuals after breakthrough infection with the SARS-CoV-2 delta variant. *Sci. Transl. Med.* **14**, eabn6150 (2022). doi:10.1126/scitranslmed.abn6150 Medline
48. Y.-J. Park, D. Pinto, A. C. Walls, Z. Liu, A. De Marco, F. Benigni, F. Zatta, C. Silacci-Fregni, J. Bassi, K. R. Sprouse, A. Addetia, J. E. Bowen, C. Stewart, M. Giurdanella, C. Saliba, B. Guarino, M. A. Schmid, N. Franko, J. Logue, H. V. Dang, K. Hauser, J. di Iulio, W. Rivera, G. Schnell, F. A. Lempp, J. Janer, R. Abdelnabi, P. Maes, P. Ferrari, A. Ceschi, O. Giannini, G. D. de Melo, L. Kergoat, H. Bourhy, J. Neyts, L. Soriagha, L. A. Purcell, G. Snell, S. P. J. Whelan, A. Lanzavecchia, H. W. Virgin, L. Piccoli, H. Chu, M. S. Pizzuto, D. Corti, D. Veesler, Imprinted antibody responses against SARS-CoV-2 Omicron sublineages. bioRxiv 2022.05.08.491108 [Preprint] (2022). https://doi.org/10.1101/2022.05.08.491108.
49. W. F. Garcia-Beltran, K. J. St. Denis, A. Hoelzemer, E. C. Lam, A. D. Nitido, M. L. Sheehan, C. Berrios, O. Ofoman, C. C. Chang, B. M. Hauser, J. Feldman, A. L. Roederer, D. J. Gregory, M. C. Poznansky, A. G. Schmidt, A. J. Iafrate, V. Naranbhai, A. B. Balazs, mRNA-based COVID-19 vaccine boosters induce neutralizing immunity against SARS-CoV-2 Omicron variant. *Cell* **185**, 457–466.e4 (2022). doi:10.1016/j.cell.2021.12.033 Medline
50. K. H. D. Crawford, R. Eguia, A. S. Dingens, A. N. Loes, K. D. Malone, C. R. Wolf, H. Y. Chu, M. A. Tortorici, D. Veesler, M. Murphy, D. Pettie, N. P. King, A. B. Balazs, J. D. Bloom, Protocol and Reagents for Pseudotyping Lentiviral Particles with SARS-CoV-2 Spike Protein for Neutralization Assays. *Viruses* **12**, 513 (2020). doi:10.3390/v12050513 Medline
51. M. Hoffmann, H. Kleine-Weber, S. Pöhlmann, A Multibasic Cleavage Site in the Spike Protein of SARS-CoV-2 Is Essential for Infection of Human Lung Cells. *Mol. Cell* **78**, 779–784.e5 (2020). doi:10.1016/j.molcel.2020.04.022 Medline
52. K. Wu, A. P. Werner, M. Koch, A. Choi, E. Narayanan, G. B. E. Stewart-Jones, T. Colpitts, H. Bennett, S. Boyoglu-Barnum, W. Shi, J. I. Moliva, N. J. Sullivan, B. S. Graham, A. Carfi, K. S. Corbett, R. A. Seder, D. K. Edwards, Serum Neutralizing Activity Elicited by mRNA-1273 Vaccine. *N. Engl. J. Med.* **384**, 1468–1470 (2021). doi:10.1056/NEJMc2102179 Medline
53. K. S. Corbett, D. K. Edwards, S. R. Leist, O. M. Abiona, S. Boyoglu-Barnum, R. A. Gillespie, S. Himansu, A. Schäfer, C. T. Ziwawo, A. T. DiPiazza, K. H. Dinnon, S. M. Elbashir, C. A. Shaw, A. Woods, E. J. Fritch, D. R. Martinez, K. W. Bock, M. Minai, B. M. Nagata, G. B. Hutchinson, K. Wu, C. Henry, K. Bahl, D. Garcia-Dominguez, L. Ma, I. Renzi, W.-P. Kong, S. D. Schmidt, L. Wang, Y. Zhang, E. Phung, L. A. Chang, R. J. Loomis, N. E. Altaras, E. Narayanan, M. Metkar, V. Presnyak, C. Liu, M. K. Louder, W. Shi, K. Leung, E. S. Yang, A. West, K. L. Gully, L. J. Stevens, N. Wang, D. Wrapp, N. A. Doria-Rose, G. Stewart-Jones, H. Bennett, G. S. Alvarado, M. C. Nason, T. J. Ruckwardt, J. S. McLellan, M. R. Denison, J. D. Chappell, I. N. Moore, K. M. Morabito, J. R. Mascola, R. S. Baric, A. Carfi, B. S. Graham, SARS-CoV-2 mRNA vaccine design enabled by prototype pathogen preparedness. *Nature* **586**, 567–571 (2020). doi:10.1038/s41586-020-2622-0 Medline
54. E. E. Walsh, R. W. Frenck Jr., A. R. Falsey, N. Kitchin, J. Absalon, A. Gurtman, S. Lockhart, K. Neuzil, M. J. Mulligan, R. Bailey, K. A. Swanson, P. Li, K. Koury, W. Kalina, D. Cooper, C. Fontes-Garfias, P.-Y. Shi, Ö. Türeci, K. R. Tompkins, K. E. Lyke, V. Raabe, P. R. Dormitzer, K. U. Jansen, U. Şahin, W. C. Gruber, Safety and Immunogenicity of Two RNA-Based Covid-19 Vaccine Candidates. *N. Engl. J. Med.* **383**, 2439–2450 (2020). doi:10.1056/NEJMoa2027906 Medline
55. D. Planas, N. Saunders, P. Maes, F. Guivel-Benhassine, C. Planchais, J. Buchrieser, W.-H. Bolland, F. Porrot, I. Staropoli, F. Lemoine, H. Péré, D. Veyer, J. Puech, J. Rodary, G. Baela, S. Dellicour, J. Raymenants, S. Gorissen, C. Geenen, B. Vanmechelen, T. Wawina-Bokalanga, J. Martí-Carreras, L. Cuypers, A. Sève, L. Hocqueloux, T. Prazuck, F. A. Rey, E. Simon-Lorriere, T. Bruel, H. Mouquet, E. André, O. Schwartz, Considerable escape of SARS-CoV-2 Omicron to antibody neutralization. *Nature* **602**, 671–675 (2022). doi:10.1038/d41586-021-03827-2 Medline
56. E. Pérez-Then, C. Lucas, V. S. Monteiro, M. Miric, V. Brache, L. Cochon, C. B. F. Vogels, A. A. Malik, E. De la Cruz, A. Jorge, M. De Los Santos, P. Leon, M. I. Breban, K. Billig, I. Yildirim, C. Pearson, R. Downing, E. Gagnon, A. Muyombwe, J. Razeq, M. Campbell, A. I. Ko, S. B. Omer, N. D. Grubaugh, S. H. Vermund, A. Iwasaki, Neutralizing antibodies against the SARS-CoV-2 Delta and Omicron variants following heterologous CoronaVac plus BNT162b2 booster vaccination. *Nat. Med.* **28**, 481–485 (2022). doi:10.1038/s41591-022-01705-6 Medline
57. R. R. Goel, M. M. Painter, K. A. Lundgreen, S. A. Apostolidis, A. E. Baxter, J. R. Giles, D. Mathew, A. Pattekar, A. Reynaldi, D. S. Khoury, S. Gouma, P. Hicks, S. Dysinger, A. Hicks, H. Sharma, S. Herring, S. Korte, K. C. Wumesh, D. A. Oldridge, R. I. Erickson, M. E. Weirick, C. M. McAllister, M. Awofolaju, N. Tanenbaum, J. Dougherty, S. Long, K. D'Andrea, J. T. Hamilton, M. McLaughlin, J. C. Williams, S. Adamski, O. Kuthuru, E. M. Drapeau, M. P. Davenport, S. E. Hensley, P. Bates, A. R. Greenplate, E. J. Wherry, Efficient recall of Omicron-reactive B cell memory after a third dose of SARS-CoV-2 mRNA vaccine. *Cell* **185**, 1875–1887.e8 (2022). doi:10.1016/j.cell.2022.04.009 Medline
58. F. Muecksch, Z. Wang, A. Cho, C. Gaebler, T. B. Tanfous, J. DaSilva, E. Bednarski, V. Ramos, S. Zong, B. Johnson, R. Raspe, D. Schaefer-Babajew, I. Shimeliovich, M. Daga, K.-H. Yao, F. Schmidt, K. G. Millard, M. Turroja, M. Jankovic, T. Y. Oliveria, A. Gazumyan, M. Caskey, T. Hatziioannou, P. D. Bieniasz, M. C. Nussenzweig, Increased Potency and Breadth of SARS-CoV-2 Neutralizing Antibodies After a Third mRNA Vaccine Dose. bioRxiv 2022.02.14.480394 [Preprint] (2022). https://doi.org/10.1101/2022.02.14.480394.
59. B. Grunau, D. M. Goldfarb, M. Asamoah-Boaheng, L. Golding, T. L. Kirkham, P. A. Demers, P. M. Lavoie, Immunogenicity of Extended mRNA SARS-CoV-2 Vaccine Dosing Intervals. *JAMA* **327**, 279–281 (2022). doi:10.1001/jama.2021.21921 Medline
60. M. Voysey, S. A. Costa Clemens, S. A. Madhi, L. Y. Weckx, P. M. Folegatti, P. K. Aley, B. Angus, V. L. Baillie, S. L. Barnabas, Q. E. Bhorat, S. Bibi, C. Briner, P. Cicconi, E. A. Clutterbuck, A. M. Collins, C. L. Cutland, T. C. Darton, K. Dheda, C. Dold, C. J. A. Duncan, K. R. W. Emary, K. J. Ewer, A. Flaxman, L. Fairlie, S. N. Faust, S. Feng, D. M. Ferreira, A. Finn, E. Galiza, A. L. Goodman, C. M. Green, C. A. Green, M. Greenland, C. Hill, H. C. Hill, I. Hirsch, A. Izu, D. Jenkin, C. C. D. Joe, S. Kerridge, A. Koen, G. Kwatra, R. Lazarus, V. Libri, P. J. Lillie, N. G. Marchevsky, R. P. Marshall, A. V. A. Mendes, E. P. Milan, A. M. Minassian, A. McGregor, Y. F. Mujadidi, A. Nana, S. D. Padayachee, D. J. Phillips, A. Pittella, E. Plested, K. M. Pollock, M. N. Ramasamy, A. J. Ritchie, H. Robinson, A. V. Schwarzbold, A. Smith, R. Song, M. D. Snape, E. Sprinz, R. K. Sutherland, E. C. Thomson, M. E. Török, M. Toshner, D. P. J. Turner, J. Vekemans, T. L. Villafana, T. White, C. J. Williams, A. D. Douglas, A. V. S. Hill, T. Lambe, S. C. Gilbert, A. J. Pollard, Oxford COVID Vaccine Trial Group, Single-dose administration and the influence of the timing of the booster dose on immunogenicity and efficacy of ChAdOx1 nCoV-19 (AZD1222) vaccine: A pooled analysis of four randomised trials. *Lancet* **397**, 881–891 (2021). doi:10.1016/S0140-6736(21)00432-3 Medline
61. H. Parry, R. Bruton, C. Stephens, C. Bentley, K. Brown, G. Amirthalingam, B. Hallis, A. Otter, J. Zuo, P. Moss, Extended interval BNT162b2 vaccination enhances peak antibody generation. *NPJ Vaccines* **7**, 14 (2022). doi:10.1038/s41541-022-00432-w Medline
62. R. P. Payne, S. Longet, J. A. Austin, D. T. Skelly, W. Dejnirattisai, S. Adele, N. Meardon, S. Faustini, S. Al-Taei, S. C. Moore, T. Tipton, L. M. Hering, A. Angyal, R. Brown, A. R. Nicols, N. Gillson, S. L. Dobson, A. Amini, P. Supasa, A. Cross, A. Bridges-Webb, L. S. Reyes, A. Linder, G. Sandhar, J. A. Kilby, J. K. Tyerman, T. Altmann, H. Hornsby, R. Whitham, E. Phillips, T. Malone, A. Hargreaves, A. Shields, A. Saei, S. Foulkes, L. Stafford, S. Johnson, D. G. Wootton, C. P. Conlon, K. Jeffery, P. C. Matthews, J. Frater, A. S. Deeks, A. J. Pollard, A. Brown, S. L. Rowland-Jones, J. Mongkolsapaya, E. Barnes, S. Hopkins, V. Hall, C. Dold, C. J. A. Duncan, A. Richter, M. Carroll, G. Screaton, T. I. de Silva, L. Turtle, P. Klenerman, S. Dunachie, PITCH Consortium, Immunogenicity of standard and extended dosing intervals of BNT162b2 mRNA vaccine. *Cell* **184**, 5699–5714.e11 (2021). doi:10.1016/j.cell.2021.10.011 Medline
63. J. Liu, A. Chandrashekar, D. Sellers, J. Barrett, C. Jacob-Dolan, M. Lifton, K. McMahan, M. Sciacca, H. VanWyk, C. Wu, J. Yu, A. Y. Collier, D. H. Barouch, Vaccines elicit highly conserved cellular immunity to SARS-CoV-2 Omicron. *Nature* **603**, 493–496 (2022). doi:10.1038/s41586-022-04465-y Medline
64. A. Tarke, C. H. Coelho, Z. Zhang, J. M. Dan, E. D. Yu, N. Methot, N. I. Bloom, B. Goodwin, E. Phillips, S. Mallal, J. Sidney, G. Filaci, D. Weiskopf, R. da Silva Antunes, S. Crotty, A. Grifoni, A. Sette, SARS-CoV-2 vaccination induces immunological T cell memory able to cross-recognize variants from Alpha to Omicron. *Cell* **185**, 847–859.e11 (2022). doi:10.1016/j.cell.2022.01.015 Medline
65. N. Andrews, J. Stowe, F. Kirsebom, S. Toffa, T. Rickeard, E. Gallagher, C. Gower,

M. Kall, N. Groves, A.-M. O'Connell, D. Simons, P. B. Blomquist, A. Zaidi, S. Nash, N. Iwani Binti Abdul Aziz, S. Thelwall, G. Dabrera, R. Myers, G. Amirthalingam, S. Gharbia, J. C. Barrett, R. Elson, S. N. Ladhani, N. Ferguson, M. Zambon, C. N. J. Campbell, K. Brown, S. Hopkins, M. Chand, M. Ramsay, J. Lopez Bernal, Covid-19 Vaccine Effectiveness against the Omicron (B.1.1.529) Variant. *N. Engl. J. Med.* **386**, 1532–1546 (2022). doi:10.1056/NEJMoa2119451 Medline

66. E. K. Accorsi, A. Britton, K. E. Fleming-Dutra, Z. R. Smith, N. Shang, G. Derado, J. Miller, S. J. Schrag, J. R. Verani, Association Between 3 Doses of mRNA COVID-19 Vaccine and Symptomatic Infection Caused by the SARS-CoV-2 Omicron and Delta Variants. *JAMA* **327**, 639–651 (2022). doi:10.1001/jama.2022.0470 Medline
67. B. Ying, S. M. Scheaffer, B. Whitener, C.-Y. Liang, O. Dmytrenko, S. Mackin, K. Wu, D. Lee, L. E. Avena, Z. Chong, J. B. Case, L. Ma, T. Kim, C. Sein, A. Woods, D. M. Berrueta, A. Carfi, S. M. Elbashir, D. K. Edwards, L. B. Thackray, M. S. Diamond, Boosting with Omicron-matched or historical mRNA vaccines increases neutralizing antibody responses and protection against B.1.1.529 infection in mice. bioRxiv 2022.02.07.479419 [Preprint] (2022). https://doi.org/10.1101/2022.02.07.479419.
68. I.-J. Lee, C.-P. Sun, P.-Y. Wu, Y.-H. Lan, I.-H. Wang, W.-C. Liu, S.-C. Tseng, S.-I. Tsung, Y.-C. Chou, M. Kumari, Y.-W. Chang, H.-F. Chen, Y.-S. Lin, T.-Y. Chen, C.-W. Chiu, C.-H. Hsieh, C.-Y. Chuang, C.-C. Lin, C.-M. Cheng, H.-T. Lin, W.-Y. Chen, P.-C. Chiang, C.-C. Lee, J. C. Liao, H.-C. Wu, M.-H. Tao, Omicron-specific mRNA vaccine induced potent neutralizing antibody against Omicron but not other SARS-CoV-2 variants. bioRxiv 2022.01.31.478406 [Preprint] (2022). https://doi.org/10.1101/2022.01.31.478406.
69. D. W. Hawman, K. Meade-White, C. Clancy, J. Archer, T. Hinkley, S. S. Leventhal, D. Rao, A. Stamper, M. Lewis, R. Rosenke, K. Krieger, S. Randall, A. P. Khandhar, L. Hao, T.-Y. Hsiang, A. L. Greninger, M. Gale Jr., P. Berglund, D. H. Fuller, K. Rosenke, H. Feldmann, J. H. Erasmus, Replicating RNA platform enables rapid response to the SARS-CoV-2 Omicron variant and elicits enhanced protection in naïve hamsters compared to ancestral vaccine. bioRxiv 2022.01.31.478520 [Preprint] (2022). https://doi.org/10.1101/2022.01.31.478520.
70. P. S. Arunachalam, Y. Feng, U. Ashraf, M. Hu, V. V. Edara, V. I. Zarnitsyna, P. P. Aye, N. Golden, K. W. M. Green, B. M. Threeton, N. J. Maness, B. J. Beddingfield, R. P. Bohm, J. Dufour, K. Russell-Lodrigue, M. C. Miranda, A. C. Walls, K. Rogers, L. Shirreff, D. E. Ferrell, N. R. Deb Adhikary, J. Fontenot, A. Grifoni, A. Sette, D. T. O'Hagan, R. Van Der Most, R. Rappuoli, F. Villinger, H. Kleanthous, J. Rappaport, M. S. Suthar, D. Veesler, T. T. Wang, N. P. King, B. Pulendran, Durable protection against SARS-CoV-2 Omicron induced by an adjuvanted subunit vaccine. bioRxiv 2022.03.18.484950 [Preprint] (2022). https://doi.org/10.1101/2022.03.18.484950.
71. M. Gagne, J. I. Moliva, K. E. Foulds, S. F. Andrew, B. J. Flynn, A. P. Werner, D. A. Wagner, I.-T. Teng, B. C. Lin, C. Moore, N. Jean-Baptiste, R. Carroll, S. L. Foster, M. Patel, M. Ellis, V.-V. Edara, N. V. Maldonado, M. Minai, L. McCormick, C. C. Honeycutt, B. M. Nagata, K. W. Bock, C. N. M. Dulan, J. Cordon, D. R. Flebbe, J. M. Todd, E. McCarthy, L. Pessaint, A. Van Ry, B. Narvaez, D. Valentin, A. Cook, A. Dodson, K. Steingrebe, S. T. Nurmukhambetova, S. Godbole, A. R. Henry, F. Laboune, J. Roberts-Torres, C. G. Lorang, S. Amin, J. Trost, M. Naisan, M. Basappa, J. Willis, L. Wang, W. Shi, N. A. Doria-Rose, Y. Zhang, E. S. Yang, K. Leung, S. O'Dell, S. D. Schmidt, A. S. Olia, C. Liu, D. R. Harris, G.-Y. Chuang, G. Stewart-Jones, I. Renzi, Y.-T. Lai, A. Malinowski, K. Wu, J. R. Mascola, A. Carfi, P. D. Kwong, D. K. Edwards, M. G. Lewis, H. Andersen, K. S. Corbett, M. C. Nason, A. B. McDermott, M. S. Suthar, I. N. Moore, M. Roederer, N. J. Sullivan, D. C. Douek, R. A. Seder, mRNA-1273 or mRNA-Omicron boost in vaccinated macaques elicits similar B cell expansion, neutralizing responses, and protection from Omicron. *Cell* **185**, 1556–1571.e18 (2022). doi:10.1016/j.cell.2022.03.038 Medline
72. S. I. Richardson, V. S. Madzorera, H. Spencer, N. P. Manamela, M. A. van der Mescht, B. E. Lambson, B. Oosthuysen, F. Ayres, Z. Makhado, T. Moyo-Gwete, N. Mzindle, T. Motlou, A. Strydom, A. Mendes, H. Tegally, Z. de Beer, T. Roma de Villiers, A. Bodenstein, G. van den Berg, M. Venter, T. de Oliviera, V. Ueckermann, T. M. Rossouw, M. T. Boswell, P. L. Moore, SARS-CoV-2 Omicron triggers cross-reactive neutralization and Fc effector functions in previously vaccinated, but not unvaccinated, individuals. *Cell Host Microbe* **30**, 880–886.e4 (2022). doi:10.1016/j.chom.2022.03.029 Medline
73. A. Rössler, L. Knabl, D. von Laer, J. Kimpel, Neutralization profile after recovery from SARS-CoV-2 omicron infection. *N. Engl. J. Med.* **386**, 1764–1766 (2022). doi:10.1056/NEJMc2201607 Medline
74. K. Stiasny, I. Medits, D. Springer, M. Graninger, J. Camp, E. Höltl, S. Aberle, M. Traugott, W. Hoepler, J. Deutsch, O. Lammel, C. Borsodi, E. Puchhammer-Stöckl, A. Zoufaly, L. Weseslindtner, J. Aberle, Human primary Omicron BA.1 and BA.2 infections result in sub-lineage-specific neutralization. Research Square [Preprint] (2022). https://doi.org/10.21203/rs.3.rs-1536794/v1.
75. T. Mao, B. Israelow, A. Suberi, L. Zhou, M. Reschke, M. A. Peña-Hernández, H. Dong, R. J. Homer, W. M. Saltzman, A. Iwasaki, Unadjuvanted intranasal spike vaccine booster elicits robust protective mucosal immunity against sarbecoviruses. bioRxiv 2022.01.24.477597 [Preprint] (2022). https://doi.org/10.1101/2022.01.24.477597.
76. J. E. Oh, E. Song, M. Moriyama, P. Wong, S. Zhang, R. Jiang, S. Strohmeier, S. H. Kleinstein, F. Krammer, A. Iwasaki, Intranasal priming induces local lung-resident B cell populations that secrete protective mucosal antiviral IgA. *Sci. Immunol.* **6**, eabj5129 (2021). doi:10.1126/sciimmunol.abj5129 Medline
77. A. O. Hassan, F. Feldmann, H. Zhao, D. T. Curiel, A. Okumura, T.-L. Tang-Huau, J. B. Case, K. Meade-White, J. Callison, R. E. Chen, J. Lovaglio, P. W. Hanley, D. P. Scott, D. H. Fremont, H. Feldmann, M. S. Diamond, A single intranasal dose of chimpanzee adenovirus-vectored vaccine protects against SARS-CoV-2 infection in rhesus macaques. *Cell Rep. Med.* **2**, 100230 (2021). doi:10.1016/j.xcrm.2021.100230 Medline
78. S. N. Langel, S. Johnson, C. I. Martinez, S. N. Tedjakusuma, N. Peinovich, E. G. Dora, P. J. Kuehl, H. Irshad, E. G. Barrett, A. Werts, S. N. Tucker, Adenovirus type 5 SARS-CoV-2 vaccines delivered orally or intranasally reduced disease severity and transmission in a hamster model. *Sci. Transl. Med.* 10.1126/scitranslmed.abn6868 (2022). doi:10.1126/scitranslmed.abn6868 Medline
79. A. A. Cohen, P. N. P. Gnanapragasam, Y. E. Lee, P. R. Hoffman, S. Ou, L. M. Kakutani, J. R. Keeffe, H.-J. Wu, M. Howarth, A. P. West, C. O. Barnes, M. C. Nussenzweig, P. J. Bjorkman, Mosaic nanoparticles elicit cross-reactive immune responses to zoonotic coronaviruses in mice. *Science* **371**, 735–741 (2021). doi:10.1126/science.abf6840 Medline
80. D. R. Martinez, A. Schäfer, S. R. Leist, G. De la Cruz, A. West, E. N. Atochina-Vasserman, L. C. Lindesmith, N. Pardi, R. Parks, M. Barr, D. Li, B. Yount, K. O. Saunders, D. Weissman, B. F. Haynes, S. A. Montgomery, R. S. Baric, Chimeric spike mRNA vaccines protect against Sarbecovirus challenge in mice. *Science* **373**, 991–998 (2021). doi:10.1126/science.abi4506 Medline
81. J. B. Case, P. W. Rothlauf, R. E. Chen, Z. Liu, H. Zhao, A. S. Kim, L. M. Bloyet, Q. Zeng, S. Tahan, L. Droit, M. X. G. Ilagan, M. A. Tartell, G. Amarasinghe, J. P. Henderson, S. Miersch, M. Ustav, S. Sidhu, H. W. Virgin, D. Wang, S. Ding, D. Corti, E. S. Theel, D. H. Fremont, M. S. Diamond, S. P. J. Whelan, Neutralizing Antibody and Soluble ACE2 Inhibition of a Replication-Competent VSV-SARS-CoV-2 and a Clinical Isolate of SARS-CoV-2. *Cell Host Microbe* **28**, 475–485.e5 (2020). doi:10.1016/j.chom.2020.06.021 Medline
82. X. Ou, Y. Liu, X. Lei, P. Li, D. Mi, L. Ren, L. Guo, R. Guo, T. Chen, J. Hu, Z. Xiang, Z. Mu, X. Chen, J. Chen, K. Hu, Q. Jin, J. Wang, Z. Qian, Characterization of spike glycoprotein of SARS-CoV-2 on virus entry and its immune cross-reactivity with SARS-CoV. *Nat. Commun.* **11**, 1620 (2020). doi:10.1038/s41467-020-15562-9 Medline
83. C. L. Hsieh, J. A. Goldsmith, J. M. Schaub, A. M. DiVenere, H. C. Kuo, K. Javanmardi, K. C. Le, D. Wrapp, A. G. Lee, Y. Liu, C. W. Chou, P. O. Byrne, C. K. Hjorth, N. V. Johnson, J. Ludes-Meyers, A. W. Nguyen, J. Park, N. Wang, D. Amengor, J. J. Lavinder, G. C. Ippolito, J. A. Maynard, I. J. Finkelstein, J. S. McLellan, Structure-based design of prefusion-stabilized SARS-CoV-2 spikes. *Science* **369**, 1501–1505 (2020). doi:10.1126/science.abd0826 Medline

ACKNOWLEDGMENTS

We thank Hideki Tani (University of Toyama) for providing the reagents necessary for preparing VSV pseudotyped viruses. We thank Josh R. Dillen and Cindy Ng for assistance with protein production. **Funding:** This study was supported by the National Institute of Allergy and Infectious Diseases (DP1AI158186 and HHSN272201700059C to D.V.), the National Institute of Health Cellular and Molecular Biology Training Grant (T32GM007270 to A.A.), a Pew Biomedical Scholars Award (D.V.), an Investigators in the Pathogenesis of Infectious Disease

Awards from the Burroughs Wellcome Fund (D.V.), Fast Grants (D.V.), the Bill & Melinda Gates Foundation (OPP1156262 to D.V.), the University of Washington Arnold and Mabel Beckman cryoEM center and the National Institute of Health grant S10OD032290 (to D.V.) and grant U01 AI151698 for the United World Antiviral Research Network (UWARN) as part of the Centers for Research in Emerging Infectious Diseases (CREID) Network. Additionally, work was supported by NIH NIAID grant CCHI AI142742 (S.C., A.S), NIAID Contract No. 75N9301900065 (A.S, D.W), and U01 CA260541-01 (D.W). D.V. is an Investigator of the Howard Hughes Medical Institute. This project has been funded in part with federal funds from the National Institute of Allergy and Infectious Diseases, National Institutes of Health, Department of Health and Human Services, under grant CCHI AI142742 (S.C., A.S). This work was additionally supported in part by LJI Institutional Funds. **Author contributions:** J.E.B., A.A., and D.V. conceived the project and designed experiments. J.E.B. and K.R.S. produced pseudotyped viruses. J.E.B. and A.A. performed neutralization assays. A.A. and H.V.D. carried out ACE2 binding assays. A.A. carried out cell-cell fusion assays. J.E.B., C.S., J.T.B., and W.K.S. carried out ELISA binding assays. J.E.B., C.S., J.T.B., W.K.S., N.C., and A.E.P. expressed and purified glycoproteins. A.C.W., I.G.K., J.K.L, N.M.F., K.A., A.S., E.D., C.M.P., J.D., Z.Z., D.W., A.S, S.C., N.T.I., D.C., J.G., G.S., R.G., and H.Y.C. provided unique reagents. D.V. supervised the project and obtained funding. J.E.B., A.A., and D.V. analyzed the data and wrote the manuscript with input from all authors. **Competing interests:** DC, HVD, NC, AEP, ED Jr, and GS are employees of Vir Biotechnology Inc. and may hold shares in Vir Biotechnology Inc. A.C.W and D.V. are named as inventors on patent applications filed by the University of Washington for SARS-CoV-2 and sarbecovirus receptor-binding domain nanoparticle vaccines. The Veesler laboratory has received a sponsored research agreement from Vir Biotechnology Inc. HYC reported consulting with Ellume, Pfizer, The Bill and Melinda Gates Foundation, Glaxo Smith Kline, and Merck. She has received research funding from Emergent Ventures, Gates Ventures, Sanofi Pasteur, The Bill and Melinda Gates Foundation, and support and reagents from Ellume and Cepheid outside of the submitted work. A.S. is a consultant for Gritstone Bio, Flow Pharma, ImmunoScape, Avalia, Moderna, Fortress, Repertoire, Gerson Lehrman Group, RiverVest, MedaCorp, and Guggenheim. SC has consulted for GSK, JP Morgan, Citi, Morgan Stanley, Avalia NZ, Nutcracker Therapeutics, University of California, California State Universities, United Airlines, Adagio, and Roche. The remaining authors declare that the research was conducted in the absence of any commercial or financial relationships that could be construed as a potential conflict of interest. **Data and materials availability:** All data associated with this manuscript are available in the main text or the supplementary materials. All further relevant source data that support the findings of this study are available from the corresponding authors upon reasonable request. Materials are available through materials transfer agreements (UBMTAs or similar agreements) with the University of Washington. **License information:** This work is licensed under a Creative Commons Attribution 4.0 International (CC BY 4.0) license, which permits unrestricted use, distribution, and reproduction in any medium, provided the original work is properly cited. To view a copy of this license, visit https://creativecommons.org/licenses/by/4.0/. This license does not apply to figures/photos/artwork or other content included in the article that is credited to a third party; obtain authorization from the rights holder before using such material.

SUPPLEMENTARY MATERIALS

science.org/doi/10.1126/science.abq0203
Materials and Methods
Figs. S1 to S12
Tables S1 to S4
References (*81*–*83*)
MDAR Reproducibility Checklist
Movies S1 to S6

Submitted 16 March 2022; accepted 12 July 2022
Published online 19 July 2022
10.1126/science.abq0203

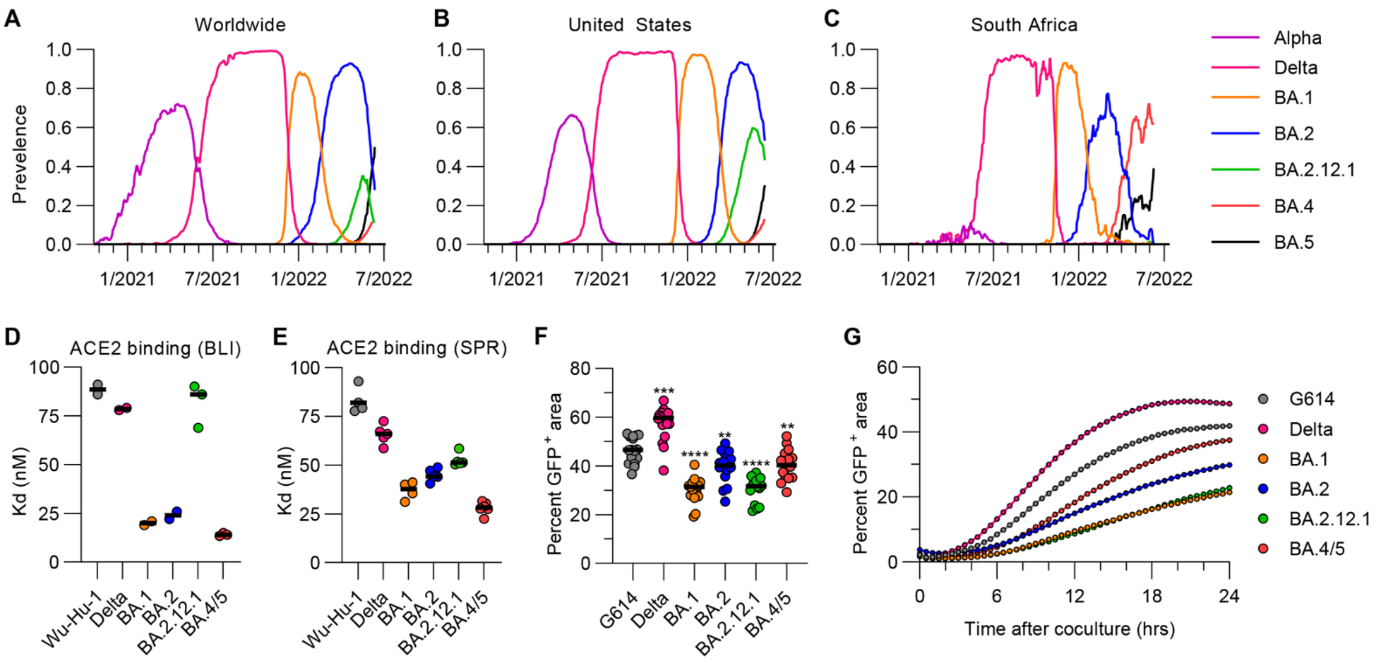

Fig. 1. Omicron sublineage RBDs bind ACE2 with enhanced affinity but exhibit impaired S-mediated fusogenicity relative to the ancestral virus. (**A-C**) Prevalence of the different variants of concern measured globally (A), in the United States (B), or in South Africa (C). Alpha comprises B.1.1.7 and all Q sublineages, Delta comprises B.1.617.2 and all AY sublineages, and BA.1, BA.2, BA.4, and BA.5 comprise their respective sublineages (including BA.2.12.1 for BA.2). Prevalence calculations rely on shared GISAID sequences and may be biased by sampling. (**D-E**) Equilibrium dissociation constants of binding of the monomeric ACE2 ectodomain to immobilized biotinylated Wuhan-Hu-1, Alpha, Delta, BA.1, BA.2, BA2.12.1, and BA.4/5 RBDs assessed by biolayer interferometry (BLI, D) or surface plasmon resonance (SPR, E). Data presented are the results of at least two independent biological replicates for BLI and for SPR (except for the BA.1 RBD SPR data which come from four technical replicates). (**F**) Quantification of cell-cell fusion after 24 hours mediated by Wuhan-Hu-1/G614, Delta, BA.1, BA.2, BA.2.12.1, and BA.4/5 S glycoproteins expressed as the fraction of the total area with GFP fluorescence assessed using a split GFP system. Data are from 16 fields of view from a single experiment and are representative of results obtained from two independent biological replicates. Comparisons between fusion mediated by the Wuhan-Hu-1/G614 S and the other variant S were completed using the Wilcoxon Rank Sum Test: **p< 0.01, ***p<0.001, ****p< 0.0001. (**G**) Kinetics of cell-cell fusion mediated by Wuhan-Hu-1/G614, Delta, BA.1, BA.2, BA.2.12.1, and BA.4/5 S glycoproteins expressed as the fraction of the total area with GFP fluorescence assessed using a split GFP system.

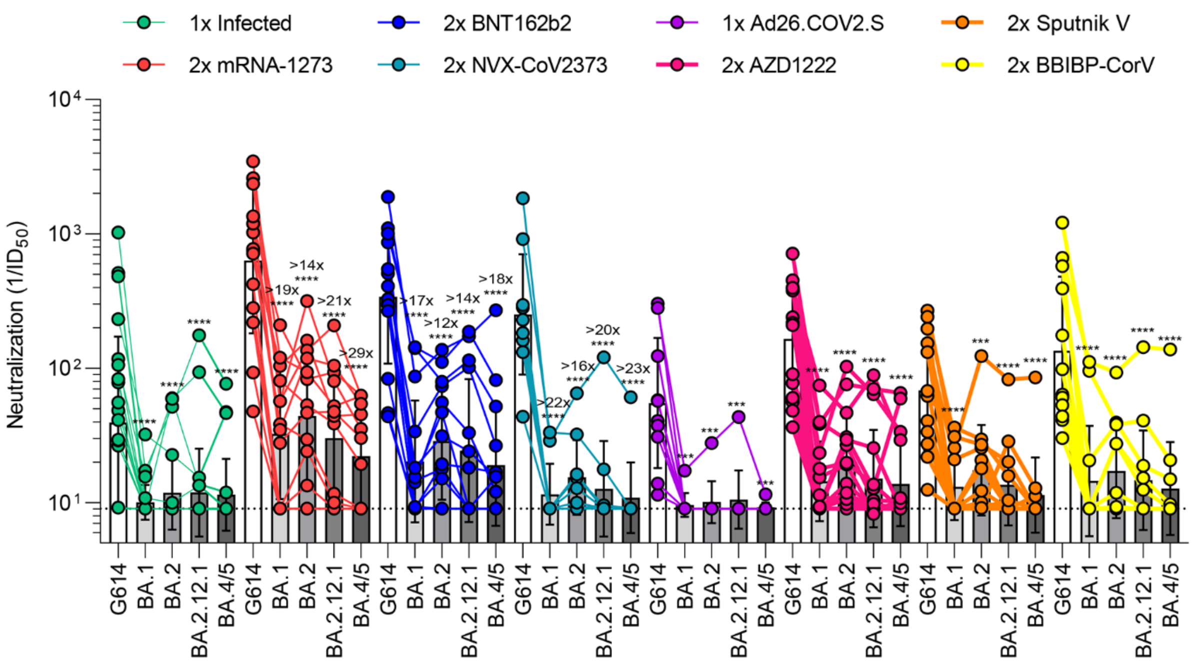

Fig. 2. SARS-CoV-2 Omicron sublineages evade human plasma neutralizing antibodies elicited by infection or primary vaccine series. Plasma neutralizing antibody titers elicited by primary COVID-19 vaccination series determined using SARS-CoV-2 S VSV pseudotypes using VeroE6/TMPRSS2 as target cells. 1x infected samples (n=24) were obtained 26-78 days (mean 41) post symptom onset, 2x mRNA-1273 samples (n=14) were obtained 6-50 days (mean 13) post second dose, 2x BNT162b2 samples (n=14) were obtained 6-33 days (mean 14) post second dose, 2x NVX-CoV2373 samples (n=10) were obtained 17-168 days (mean 82) post second dose, 1x Ad26.COV2.S samples (n=10) were obtained 9-142 days (mean 79) post first dose, 2x AZD1222 samples (n=16) were obtained approximately 30 days post second dose, 2x Sputnik V samples (n=12) were obtained 60-90 days post second dose, and BBIBP-CorV samples (n=12) were obtained 9-104 days (mean 69) post second dose. Individual points are representative geometric mean titers from two independent experiments consisting of two replicates each. Bars represent geometric means and error bars represent geometric standard deviations for each group. Statistical significance between groups of paired data was determined by Wilcoxon rank test and ***$p < 0.001$, ****$p < 0.0001$. Patient demographics are shown in table S4. Normalized curves and fits are shown in fig. S5. G614: Wuhan-Hu-1/G614.

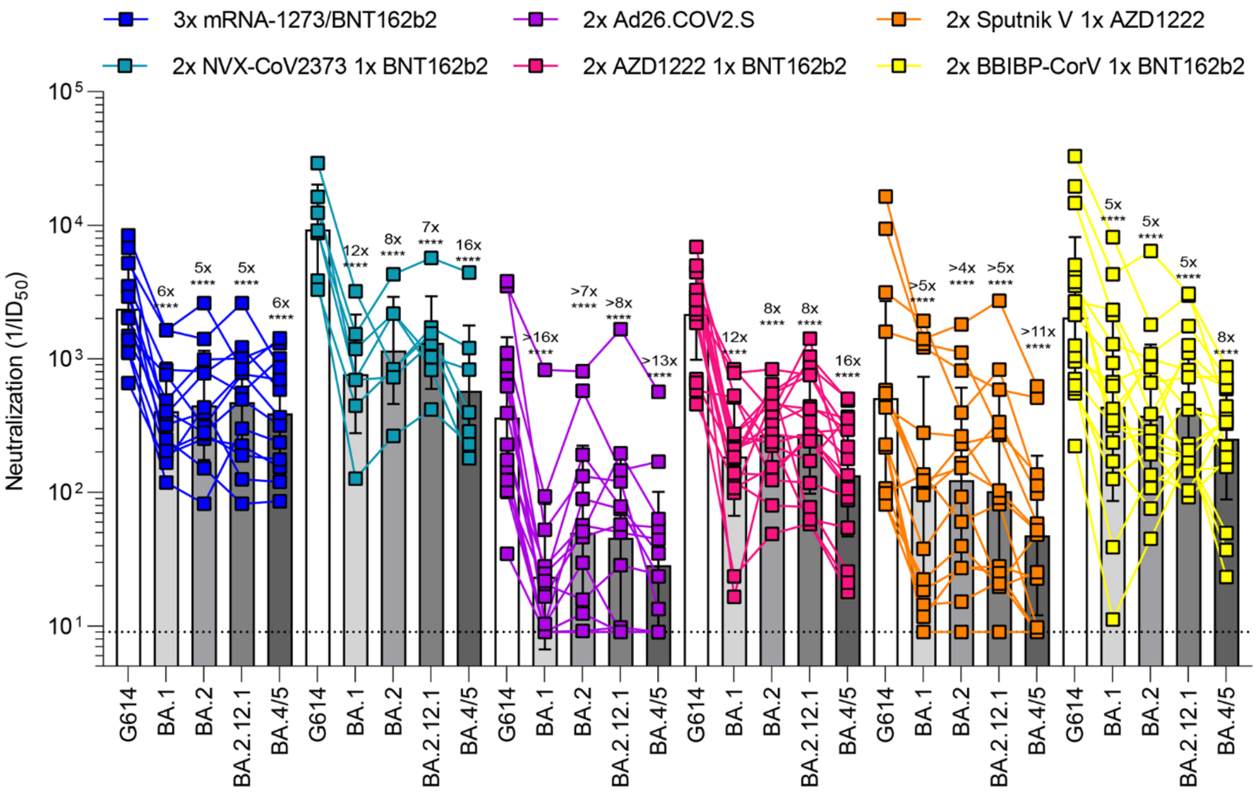

Fig. 3. Administration of a booster dose rescues neutralization potency against Omicron sublineages for all vaccines. Plasma neutralizing antibody titers elicited by COVID-19 vaccine boosters determined using SARS-CoV-2 S VSV pseudotypes and VeroE6/TMPRSS2 as target cells. 3x mRNA-1273/BNT162b2 samples (n=13) were donated 13-97 days (mean 30) post third dose, 2x NVX-CoV2373 1x mRNA-1273/BNT162b2/Ad26.COV2.S samples (n=7) were donated 5-20 days (mean 13) post third dose, 1x Ad26.COV2.S 1x Ad26.COV2.S/BNT162b2 samples (n=14) were donated 12-16 days (mean 14) post second dose, 2x AZD1222 1x BNT162b2/mRNA-1273 samples (n=18) were donated 30-123 days (mean 87) post third dose, 2x Sputnik V 1x AZD1222/BNT162b2 samples (n=14) were donated 45-60 days post third dose, and 2x BBIBP-CorV 1x BNT162b2/mRNA-1273 samples (n=18) were donated 29-89 days (mean 50) post third dose. Individual points are representative geometric mean titers from two to four independent experiments consisting of two replicates each. Bars represent geometric means and error bars represent geometric standard deviations for each group. Statistical significance between groups of paired data was determined by Wilcoxon rank test and $^{**}p < 0.01$, $^{***}p < 0.001$, $^{****}p < 0.0001$. Patient demographics are shown in table S4. Normalized curves and fits are shown in fig. S8. G614: Wuhan-Hu-1/G614.
