# Supplementary material for: Omicron spike function and neutralizing activity elicited by a comprehensive panel of vaccines
Source: Science. 2022 Jul 19;377(6608):890–4. doi: 10.1126/science.abq0203 (PMC9348749; doi:10.1126/science.abq0203)
Supplement: Supplementary file 2 — Materials and Methods Figs. S1 to S12 Tables S1 to S4 References (81–83) [file science.abq0203_sm.pdf]

Science
AAAS

Supplementary Materials for

Omicron spike function and neutralizing activity elicited by a comprehensive panel of vaccines

John E. Bowen *et al*.

Corresponding author: David Veesler, dveesler@uw.edu

Science **377**, 890 (2022)
DOI: 10.1126/science.abq0203

The PDF file includes:

Materials and Methods
Figs. S1 to S12
Tables S1 to S4
References

Other Supplementary Material for this manuscript includes the following:

Movies S1 to S6
MDAR Reproducibility Checklist

Methods

Variant incidence analysis

Average daily prevalence for Alpha (containing B.1.1.7 and all Q sublineages), Delta (containing B.1.617.2 and all AY sublineages), BA.1 (containing all BA.1 sublineages), BA.2 (containing all BA.2 sublineages, including BA.2.12.1), BA.2.12.1, BA.4 (containing all BA.4 sublineages), and BA.5 (containing all BA.5 sublineages) were obtained from GISAID using outbreak.info and RStudio and plotted using GraphPad PRISM software version 9.4.0.

Cell lines

Cell lines used in this study were obtained from ThermoFisher Scientific (HEK293T), Kerafast (BHK-21 clone WI-2), or were kindly gifted by Florian Lempp (VeroE6/TMPRSS2 cells (*43*)) or Jesse Bloom (HEK293T/ACE2) (*50*). Cell lines stably expressing components of the split GFP system were generated as described below. None of the cell lines used were authenticated or tested for mycoplasma contamination.

Generation of split GFP stable lines

To generate cell lines stably expressing either GFP_{1-10} or GFP_{11}, MLV retrovirus was generated as previously described (*22*) using either the transfer vector pQCXIP-GFP_{1-10} (Addgene; a gift from Yutaka Hatat) or pQCXIP-BSR-GFP_{11} (Addgene; a gift from Yutaka Hatat) (*42*). BHK-21 or Vero/TMPRSS2 were transduced with MLV-GFP_{1-10} or MLV-GFP_{11}, respectively, and stable lines were selected for using 2 μg/mL of puromycin or 4 μg/mL of blasticidin. The BHK-21-GFP_{1-10} line was maintained in DMEM supplemented with 10% FBS, 1% Pen-strep, and 2 μg/mL puromycin. The Vero/TMPRSS2-GFP_{11} line was maintained in DMEM supplemented with 10% FBS, 1% Pen-strep, 8 μg/mL puromycin, and 4 μg/mL blasticidin.

Sample donors

Convalescent plasma, Ad26.COV2.S, and some BNT162b2 samples were obtained from the HAARVI study approved by the University of Washington Human Subjects Division Institutional Review Board (STUDY00000959). mRNA-1273 and the rest of BNT162b2 samples were obtained from individuals enrolled in the UWARN: COVID-19 in WA study approved by the University of Washington Human Subjects Division Institutional Review Board (STUDY00010350). AZD1222 samples were obtained from the PolImmune-COVID study conducted by INGM and IRCCS Ca' Granda Ospedale Maggiore Policlinico of Milan, approved by INMI "Lazzaro Spallanzani" Ethics Committee (286_2021). Samples from NVX-CoV2373 immunized individuals were collected in the San Diego region by the La Jolla Institute for Immunology (*44*). This work was approved by the institutional review board (IRB) of the La Jolla Institute (IRB#: VD-214). Sputnik V samples were obtained from healthcare workers at the hospital de Clínicas "José de San Martín", Buenos Aires, Argentina. BBIBP-CorV samples were obtained from Aga Khan University, Karachi, Pakistan. Demographic data for these individuals are summarized in Table S4. All samples were tested for binding to the SARS-CoV-2 nucleocapsid by ELISA and plasma with area under the curve values ≥4.0 were excluded from the neutralization assay due to likely prior infection (**fig. S11-12**). Excluded samples (with predicted prior infection) included two individuals that received two Sputnik V doses and two individuals that received two Sputnik V doses followed by a single AZD1222 dose.

Plasmid construction

Full length S constructs used for pseudovirus production: the SARS-CoV-2 G614 S (YP 009724390.1) gene was placed into the HDM vector with a 21 residue C-terminal deletion, as previously described (*3*, *40*, *82*). The plasmids encoding the SARS-CoV-2 Omicron S variants BA.1 and BA.2 were generated by overlap PCR mutagenesis of the wildtype plasmid,

pcDNA3.1(+)-spike-D19 (*12*, *83*). The SARS-CoV-2 BA.2.12.1 and BA.4/5 Omicron subvariant S genes were subcloned by Genscript via mutagenesis of the G614 S construct (**table S1**).

Proteins used for BLI: the SARS-CoV-2 Wuhan-Hu-1 RBD construct was synthesized by GenScript into pcDNA3.1- with an N-terminal mu-phosphatase signal peptide and a C-terminal octa-histidine tag, flexible linker, and avi tag (GHHHHHHHHGGSSGLNDIFEAQKIEWHE). The boundaries of the construct are N-328RFPN331 and 528KKST531-C (*41*). The SARS-CoV-2 Delta (B.1.617.2) RBD (L452R, T478K) construct and the SARS-CoV-2 Omicron BA.1 RBD construct (G339D, S371L, S373P, S375F, K417N, N440K, G446S, S477N, T478K, E484A, Q493R, G496S, Q498R, N501Y, Y505H) are described as above and were cloned into the CMVR plasmid by GenScript. The SARS-CoV-2 Omicron BA.2 RBD (G339D, S371F, S373P, S375F, T376A, D405N, R408S, K417N, N440K, S477N, T478K, E484A, Q493R, Q498R, N501Y, Y505H) construct was synthesized by Genscript into pcDNA3.1(+) with a BM40+A (for Kozak) signal peptide and a C-terminal avi tag, flexible linker, and octa-histidine tag (GGLNDIFEAQKIEWHEGSGHHHHHHHH*). The boundaries of the construct are N-328RFPN331 and 528KKST531-C. Genscript mutagenized the BA.2 RBD construct to produce the SARS-CoV-2 BA.2.12.1 (BA.2 mutations with L452Q) and BA.4/5 (BA.2 mutations with L452R, F486V, R493Q (Wuhan-Hu-1 reversion)) RBD constructs.

Proteins used for SPR: The SARS-CoV-2 Wuhan-Hu-1 RBD construct contains S residues 328-531 (GenBank NC_045512.2) with an N-terminal signal peptide and a C-terminal 8xHis-AviTag. RBDs from other SARS-CoV-2 variants were obtained by Gibson Assembly (New England Biolabs) or In-Fusion (Takara) using the digested wildtype plasmid and gBlocks Gene Fragments (IDT) encoding the variant RBD sequences. All plasmids were verified by sequencing. The ACE2 construct encodes for residues 19-615 from Uniprot Q9BYF1 with an N-terminal signal peptide and a C-terminal thrombin cleavage site-TwinStrep-10xHis-GGG-tag.

Recombinant protein production for BLI and ELISA

SARS-CoV-2 S or RBDs were produced in Expi293F Cells (ThermoFisher Scientific) grown in suspension using Expi293 Expression Medium (ThermoFisher Scientific) at 37°C in a humidified 8% CO_2 incubator rotating at 130 rpm. Cells grown to a density of 3 million cells per mL were transfected using the ExpiFectamine 293 Transfection Kit (ThermoFisher Scientific) and cultivated for 3-5 days. Proteins were purified from clarified supernatants using a nickel HisTrap HP affinity column (Cytiva) and washed with ten column volumes of 20 mM imidazole, 25 mM sodium phosphate pH 8.0, and 300 mM NaCl before elution on a gradient to 500 mM imidazole, 25 mM sodium phosphate pH 8.0, and 300 mM NaCl. Proteins were buffer exchanged into 20 mM sodium phosphate pH 8 and 100 mM NaCl and concentrated using centrifugal filters (Amicon Ultra) before being flash frozen.

Recombinant protein production for SPR assays

SARS-CoV-2 RBDs were expressed in Expi293F cells (Thermo Fisher Scientific) at 37 °C and 8% CO_2. Transfections were performed using the ExpiFectamine 293 Transfection Kit (Thermo Fisher Scientific). Cell culture supernatants were harvested five days after transfection by spinning at 4,000 xg for 20 min. Supernatants were then filtered through a 0.22-µm filter and supplemented with 10x PBS to a final concentration of 2.5x PBS (342.5 mM NaCl, 6.75 mM KCl and 29.75 mM phosphates). SARS-CoV-2 RBDs were purified using HisPur Cobalt resin (Thermo Fisher Scientific) or 5 ml HisTALON Superflow cartridges (Takara) followed by buffer exchange into PBS using Amicon centrifugal filters (MilliporeSigma) or by size exclusion chromatography using a Superdex 200 Increase 10/300 GL column (Cytiva). Recombinant human ACE2 was expressed in Expi293F cells at 37°C and 8% CO2. Transfection was performed using the ExpiFectamine 293 transfection kit (Thermo Fisher Scientific). Cell

culture supernatant was collected 7-8 days after transfection and supplemented to a final concentration of 80 mM Tris-HCl pH 8.0, 100 mM NaCl, and then incubated with BioLock (IBA GmbH) solution. ACE2 was purified using a 1 mL StrepTrap HP column (Cytiva) followed by size exclusion chromatography using a Superdex 200 Increase 10/300 GL column (Cytiva) pre-equilibrated in PBS.

Pseudotyped VSV production
SARS-CoV-2 G614 and Omicron BA.1, BA.2, BA.2.12.1, and BA.4/5 pseudotypes were prepared similarly as previously described (*3*). Briefly, HEK293T cells seeded in poly-D-lysine-coated 100 mm dishes at ~75 % confluency were washed five times with Opti-MEM and transfected using 24 µg of the S glycoprotein plasmid with Lipofectamine 2000 (Life Technologies). After 5 h at 37°C, media supplemented with 20% FBS and 2% PenStrep was added. After 20 hours, cells were washed five times with DMEM and cells were transduced with VSVΔG-luc before a 2 h incubation at 37°C. Infected cells were then washed an additional five times with DMEM prior to adding media supplemented with anti-VSV-G antibody (I1-mouse hybridoma supernatant diluted 1:25, from CRL-2700, ATCC) to reduce parental background. After 18-24 h, the supernatant was harvested and clarified by low-speed centrifugation at 2,500 g for 10 min. The supernatant was then filtered (0.45 µm) and concentrated 10 times using a 30 kDa centrifugal concentrator (Amicon Ultra). The pseudotypes were then aliquoted and frozen at -80 °C.

Pseudotyped VSV neutralization assay
To evaluate neutralization of SARS-CoV-2 G614 and Omicron BA.1, BA.2, BA.2.12.1, and BA.4/5 pseudotypes by plasma of vaccinees or previously infected individuals, VeroE6/TMPRSS2 or HEK293T/hACE2 cells in DMEM supplemented with 10% FBS, 1% PenStrep, and 8 ug/mL puromycin were seeded at 60-70% confluency into white clear-bottom 96 well plates (Corning) and incubated at 37°C. The following day, a half-area 96-well plate (Greiner) was prepared with eight 3-fold serial plasma dilutions. An equal volume of DMEM with pseudovirus and 1:20 anti-VSV-G antibody (I1-mouse hybridoma supernatant from CRL-2700, ATCC) was then added to the half-area plate. Added pseudovirus was 1:15-1:50 depending on the batch to normalize entry levels, and the same dilution was added regardless of target cell line. The mixture was incubated at room temperature for 20-30 minutes. Media was removed from the cells and 40 µL from each well (containing plasma and pseudovirus) was transferred to the 96-well plate seeded with Vero/TMPRSS2 cells and incubated at 37°C for 2 h. After 2 h, an additional 40 µL of DMEM supplemented with 20% FBS and 2% PenStrep was added to the cells. After 16-20 h, 40 µL of One-Glo-EX substrate (Promega) was added to each well and incubated on a plate shaker in the dark for 5 min. Relative luciferase units were read using a Biotek plate reader. Relative luciferase units were plotted and normalized in Prism (GraphPad): 100% neutralization being cells lacking pseudovirus and 0% neutralizing being cells containing virus but lacking plasma. Prism (GraphPad) nonlinear regression with "[inhibitor] versus normalized response with a variable slope" was used to determine ID_{50} values from curve fits with 2-3 repeats. 2-4 biological replicates consisting of distinct pseudovirus batches were carried out for each sample.

Cell-cell Fusion Assay
The day prior to the cell fusion assay, Vero-TMPRSS2-GFP_{11} cells were split into 96-well, glass bottom, black walled plates (CellVis) at a density of 18,000 cells per well. BHK-21-GFP_{1-10} cells were split into 6-well plates at a density of $1x10^6$ cells per well. The following day, the growth media was removed and replaced with DMEM containing 10% FBS and 1% Pen-strep and the cells were transfected with 4 µg of S protein using Lipofectamine 2000 (Life Technology). Twenty-four hours after transfection, BHK-21-GFP_{1-10} expressing the S protein were washed three times using FluoroBrite DMEM (Thermo Fisher) and detached using an enzyme-free cell dissociation buffer (Gibco). The Vero/TMPRSS2-GFP_{11} were washed three times with FluoroBrite DMEM and

12,000 BHK-21-GFP_{1-10} cells were plated on top of the Vero/TMPRSS2-GFP_{11} cells. The cells were incubated at 37°C for 18 h and then imaged with a Cytation 7 Imager (BioTek). Fusogenicity was determined by measuring the area showing GFP fluorescence for each image using Gen5 Image Prime v3.11 software and comparisons between the different S variant proteins were assessed using the Wilcoxon Rank Sum Test in Prism 9.

To measure S expression at the surface of BHK-21-GFP_{1-10} cells, $1x10^6$ cells were collected by centrifugation at 1,000 x g for 5 mins. The cells were washed once with flow staining buffer (1% BSA, 1 mM EDTA, 0.1% NaN_3 in PBS) and labeled with 250 µg/mL of S2L20 (*81*) , for 45 mins, an NTD-directed antibody that binds to all currently characterized SARS-CoV-2 variants. The cells were washed twice with flow staining buffer and labeled with a PE-conjugated anti-Human IgG Fc antibody (Thermo Fisher) for 30 mins. The cells were washed twice with flow staining buffer and once with PBS. The cells were fixed with 2% paraformaldehyde for 15 mins and washed twice with PBS. The labeled cells were analyzed using a BD FACSAria III. Cells were gated on singleton events and a total of 10,000 singleton events were collected for each sample. The fraction of S-positive cells was determined in FlowJo 10.8.1 by gating singleton events for the mock transfected cells on PE intensity.

Biolayer interferometry
All steps of the affinity measurements using biolayer interferometry were carried out at 30ºC with a shaking speed of 1,000 r.p.m. Biotinylated Wuhan-Hu-1, Delta, BA.1, BA.2, BA.2.12.1, or BA.4/5 RBDs were diluted to a concentration of 5 ng/µL in 10X kinetics buffer and loaded onto pre-hydrated streptavidin biosensors to a 1 nm total shift. The loaded tips were dipped into a 1:3 dilution series of monomeric human ACE2 starting at 900 nM or 300 nM for 300 seconds followed by dissociation in 10X kinetics buffer for 300 seconds. The resulting data were baseline subtracted and curves were globally fitted using a 1:1 binding model with Octet Data Analysis HT software v12.0 and plotted in GraphPad Prism 9.

Surface plasmon resonance
Measurements were performed using a Biacore T200 instrument. A CM5 chip with covalently immobilized anti-Avi polyclonal antibody (GenScript, Cat #: A00674-40) was used for surface capture of His-Avi tag-containing RBDs. Running buffer was HBS-EP+ pH 7.4 (Cytiva) and measurements were performed at 25℃. Experiments were performed with a 4-fold dilution series of monomeric ACE2 protein: 11.11, 33.33, 100, 300 mM and were run as single-cycle kinetics. Data were double reference-subtracted and fit to a binding model using Biacore Evaluation software. The 1:1 binding model was used to estimate the kinetics parameters. Each RBD-ACE2 pair were measured in at least 4 replicates consisting of a single biological replicate for the BA.1 RBD and two for all other tested RBDs.

Enzyme-linked immunosorbent assay (ELISA)
30 µL of 3 µg/mL prefusion SARS-CoV-2 nucleocapsid protein (The Native Antigen Company) was diluted in PBS and incubated on a 384-well Nunc Maxisorp plate (ThermoFisher 464718) for one hour at 37˚C. Plates were slapped dry before addition of 80 µL blocker Casein in PBS (ThermoFisher) and incubation for one hour at 37˚C. Plates were slapped dry and a 1:4 serial dilution of plasma in 30 µL TBST was added and incubated for one hour at 37˚C. Plates were slapped dry and washed 4x with TBST using a BioTek plate washer followed by addition of Invitrogen anti-Human IgG (ThermoFisher A18817) and one hour incubation at 37˚C. Plates were once again slapped dry and washed 4x with TBST before addition of room temperature TMB Microwell Peroxidase (Seracare 5120-0083). The reaction was quenched after 1-2 minutes with 1 N HCl and the absorbance at 450nm (A_{450}) of each well was read using a BioTek plate reader.

The x axes were log transformed and Prism (GraphPad) “Area Under Curve” was used to determine binding strength.

Supplementary Material

Movies S1-S6. Representative movies of real-time cell-cell fusion captured every 30 minutes over a 24-hour period with effector cells expressing SARS-CoV-2 S with the G614 (S1), Delta (S2), BA.1 (S3), BA.2 (S4), BA.2.12.1 (S5), or BA.4/5 (S6) mutations.

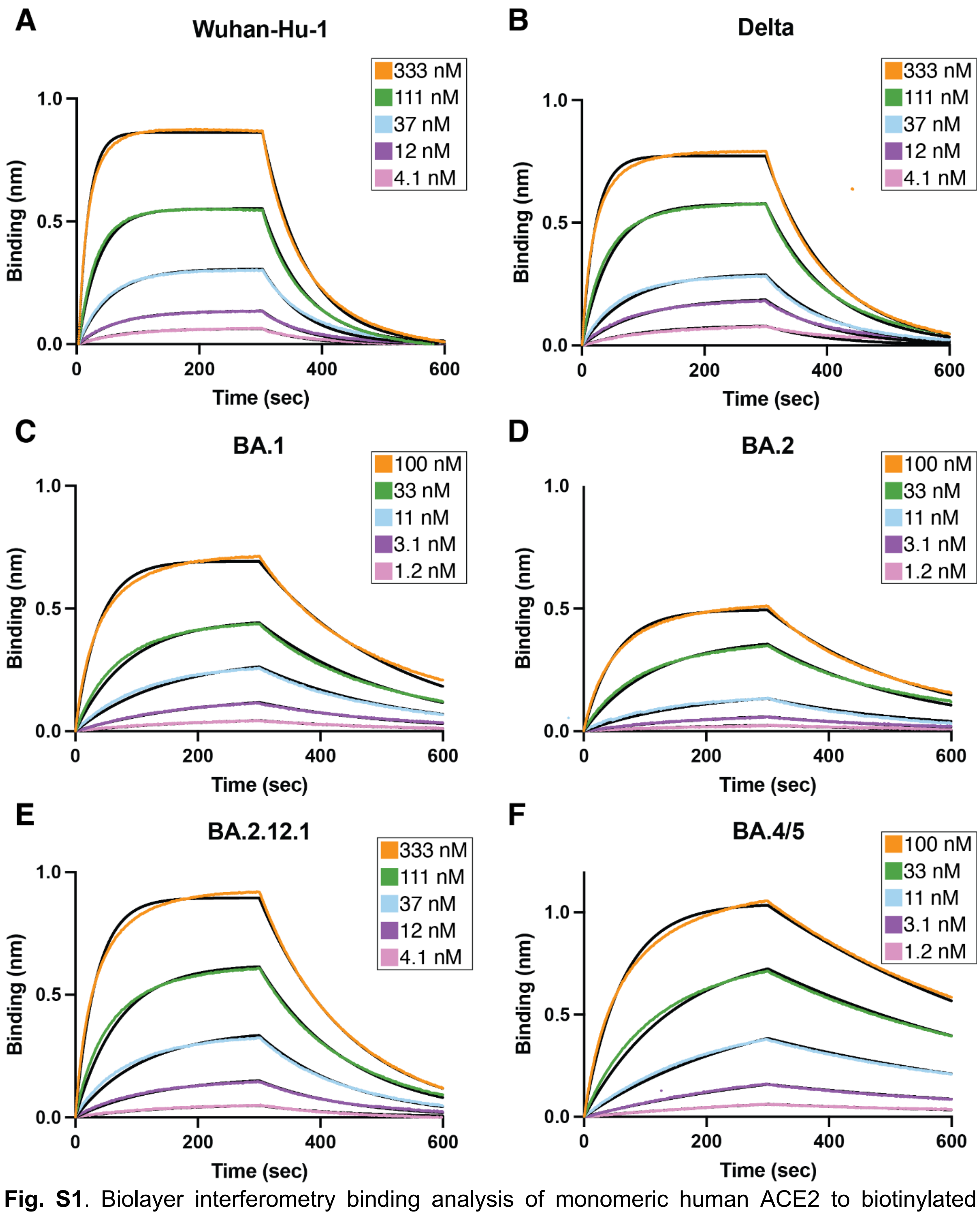

Fig. S1. Biolayer interferometry binding analysis of monomeric human ACE2 to biotinylated Wuhan-Hu-1 (A), Delta (B), BA.1 (C), BA.2 (D), BA.2.12.1 (E), or BA.4/5 (F) RBDs immobilized at the surface of SA biosensors. Kinetic rate constants and affinities are presented in Table S1.

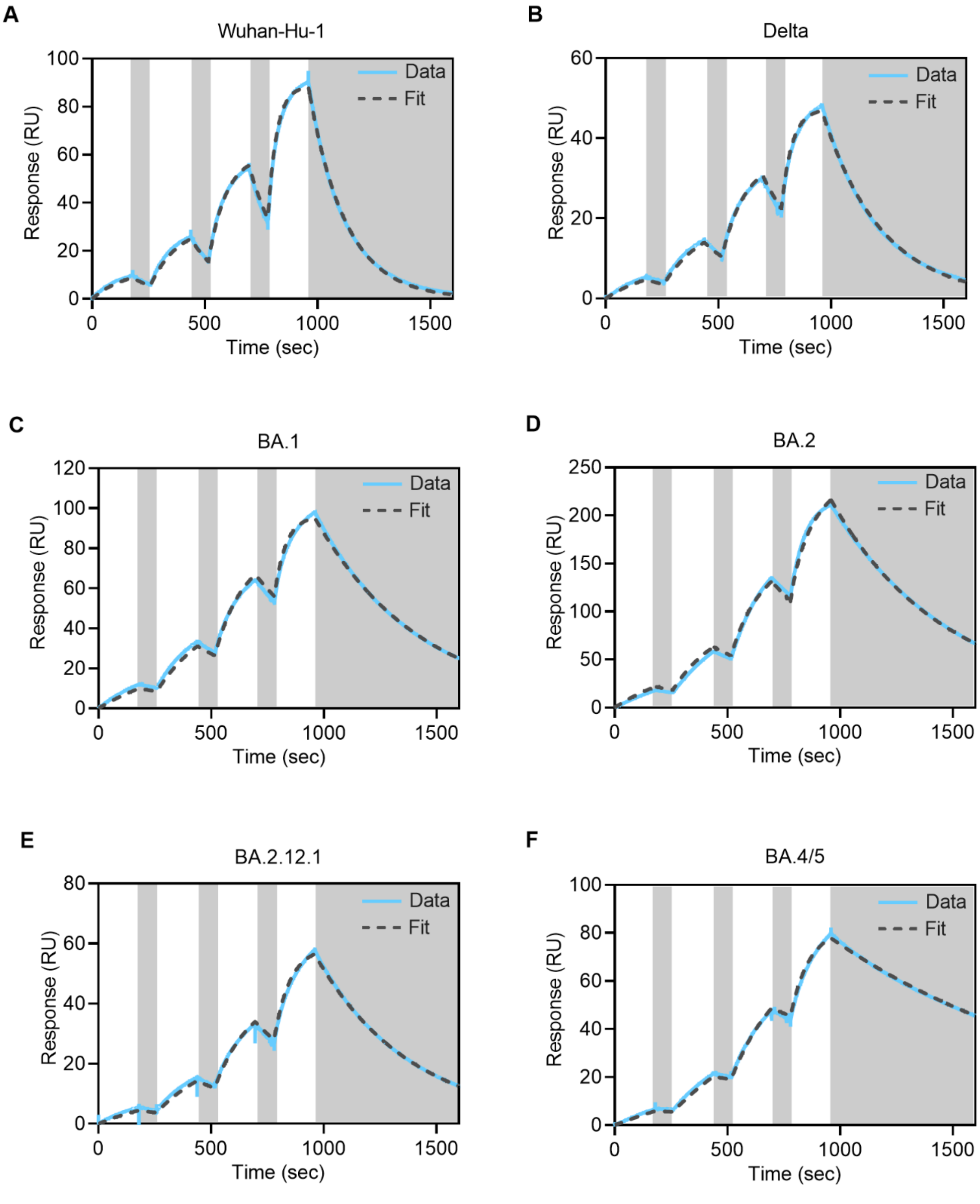

Fig. S2. Representative surface plasmon resonance binding analysis of monomeric human ACE2 to Wuhan-Hu-1 (A), Delta (B), BA.1 (C), BA.2 (D), BA.2.12.1 (E), or BA.4/5 (F) RBDs immobilized at the surface of a SPR chip coated with anti-Avi polyclonal antibody. Experiments were performed with a 3-fold dilution series of Fab: 11.11, 33.33, 100, 300nM, and were run as single-cycle kinetics. Gray blocks denote the dissociation phase.

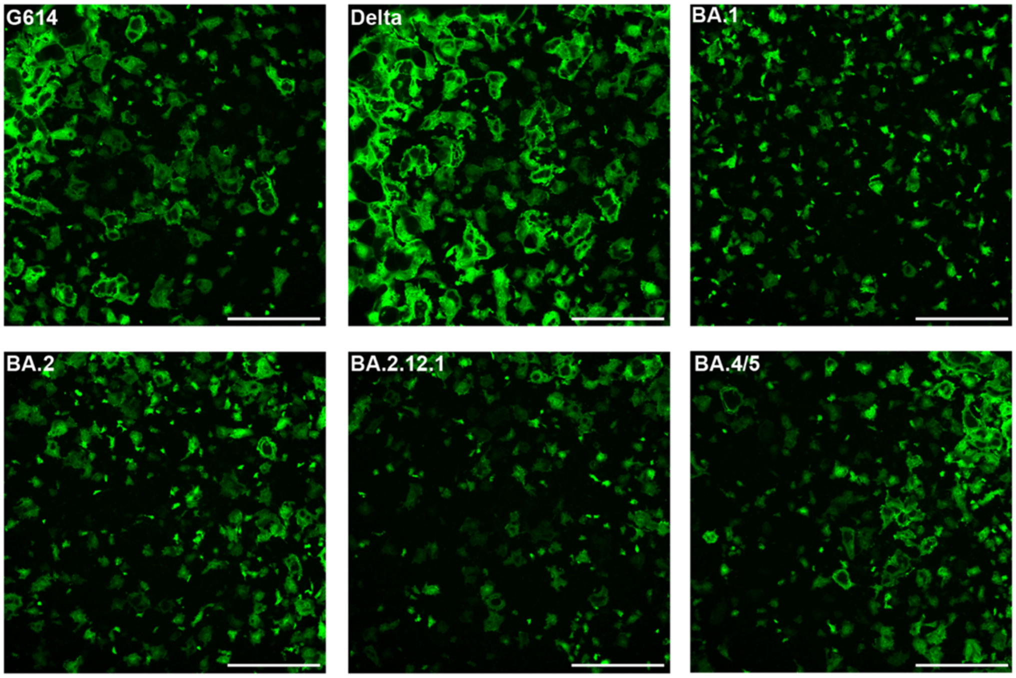

Fig. S3. Representative cell-cell fusion fluorescence images after 24 h. Scale bar: 1 mm.

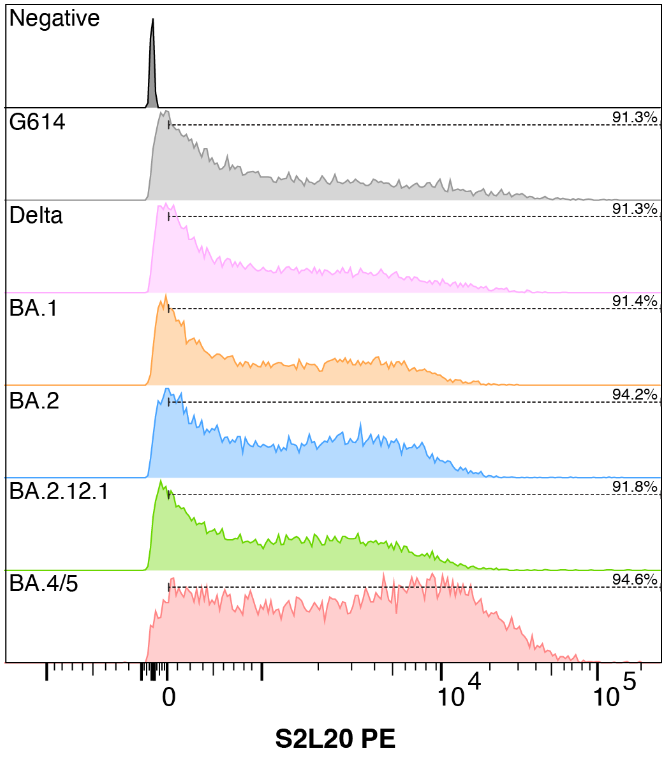

Fig. S4. Evaluation of cell surface S expression determined by labeling effector cells with the broadly reactive NTD-directed S2L20 antibody (*81*) and measuring PE intensity by flow cytometry. The y-axis of each histogram is presented as a modal scale proportional to the maximum cell count for that plot. The percentage of S-positive cells based on gating for singleton events for the

mock transfected (negative) cells, represented by the dashed lines, for each sample is shown above each histogram.

A 1x Infected

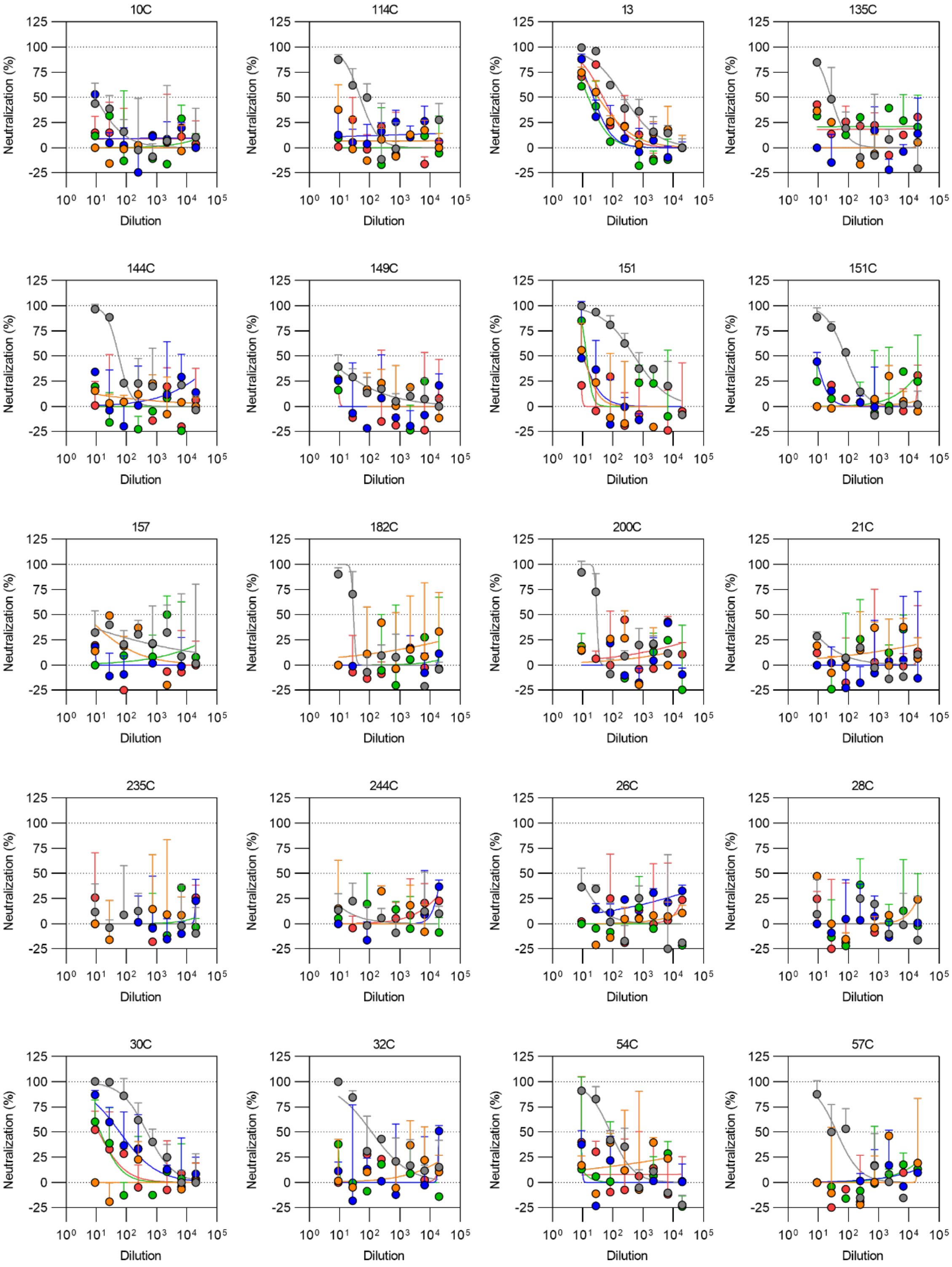

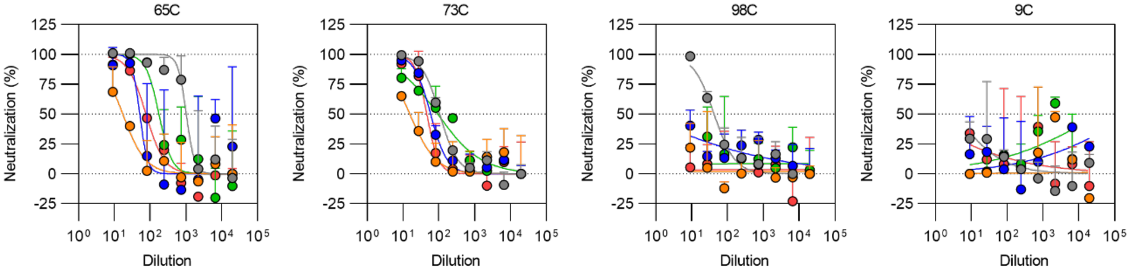

B 2x mRNA-1273

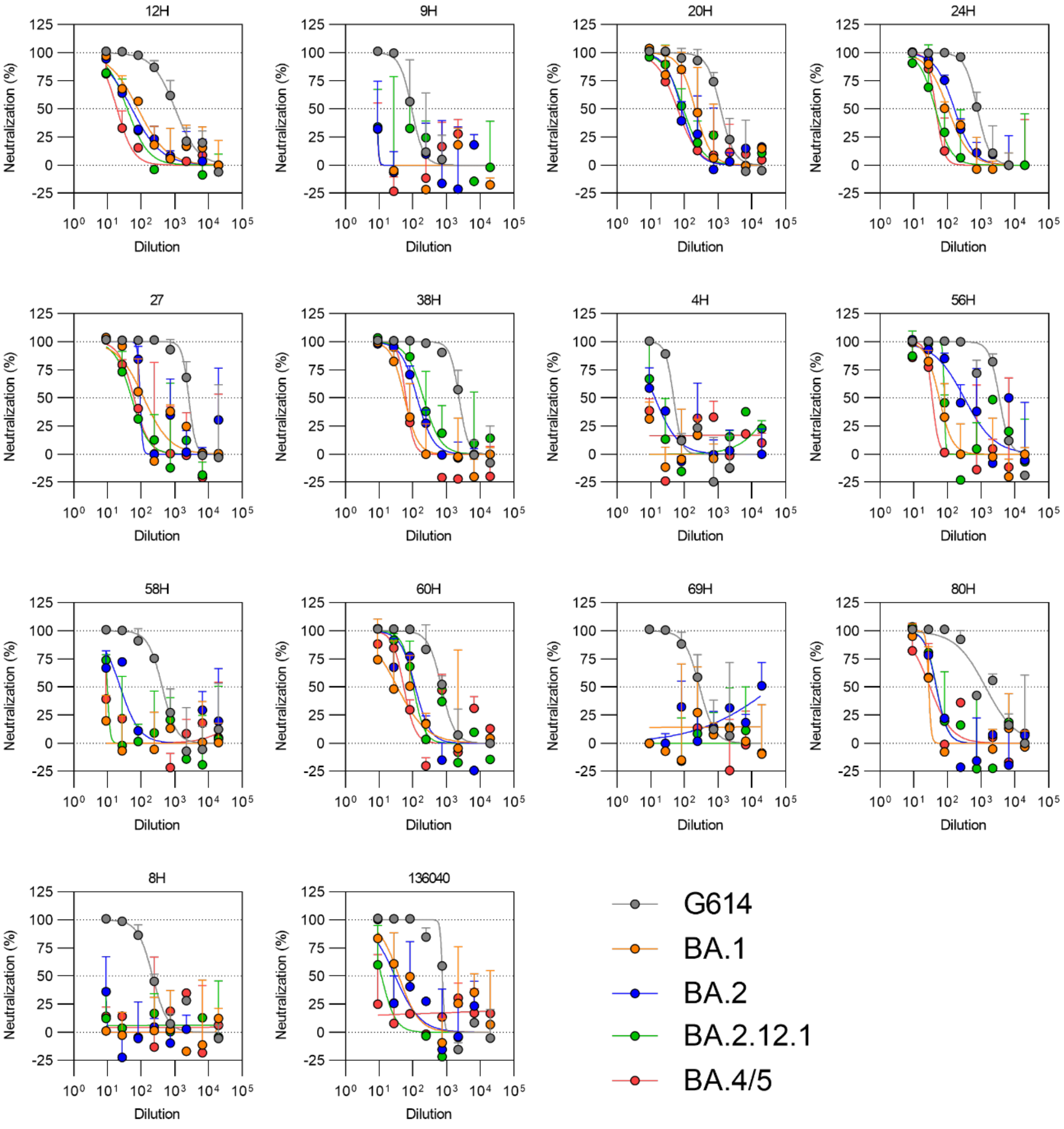

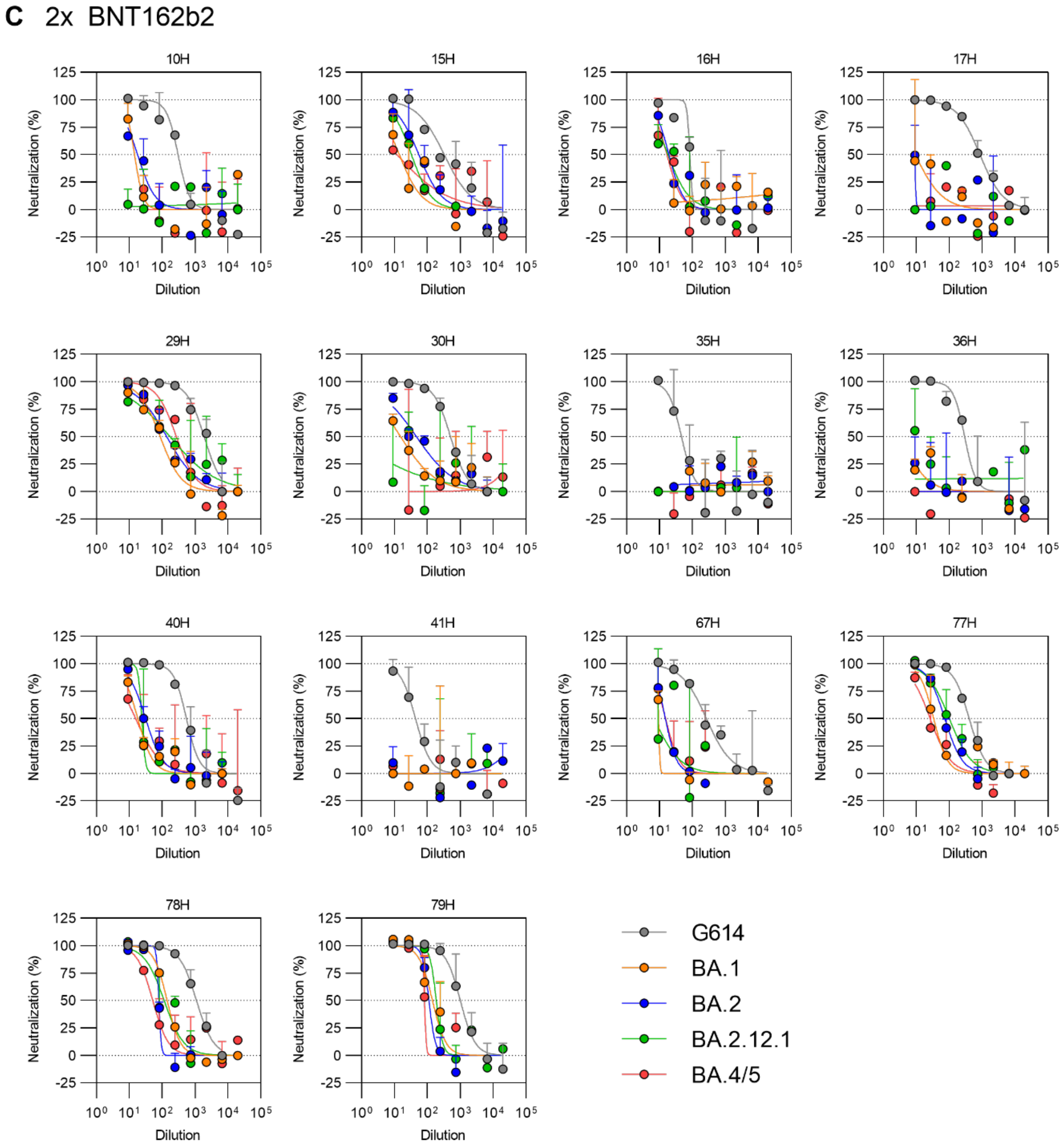
C 2x BNT162b2
10H
15H
16H
17H
29H
30H
35H
36H
40H
41H
67H
77H
78H
79H
Neutralization (%)
Dilution
G614
BA.1
BA.2
BA.2.12.1
BA.4/5

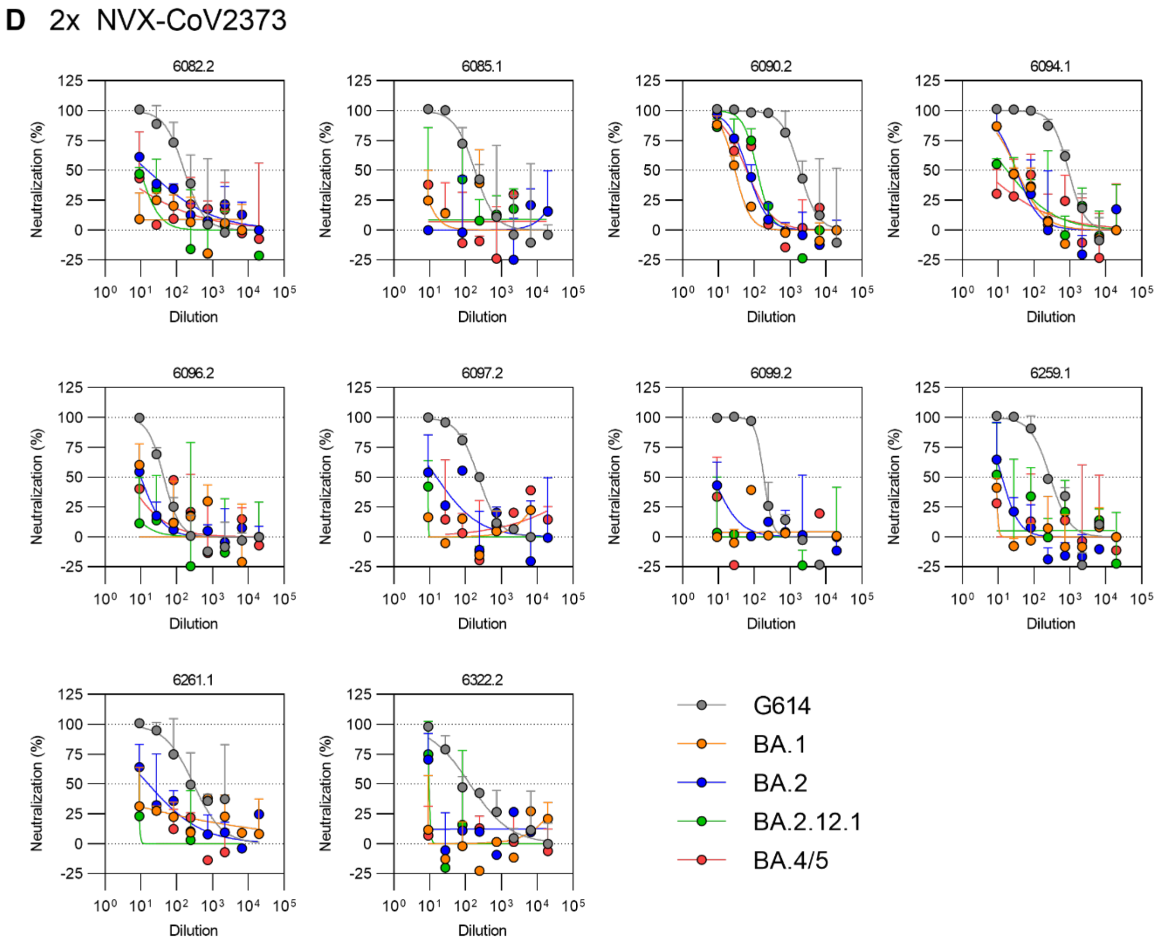
D 2x NVX-CoV2373
6082.2
6085.1
6090.2
6094.1
6096.2
6097.2
6099.2
6259.1
6261.1
6322.2
Neutralization (%)
Dilution
G614
BA.1
BA.2
BA.2.12.1
BA.4/5

E 1x Ad26.COV2.S

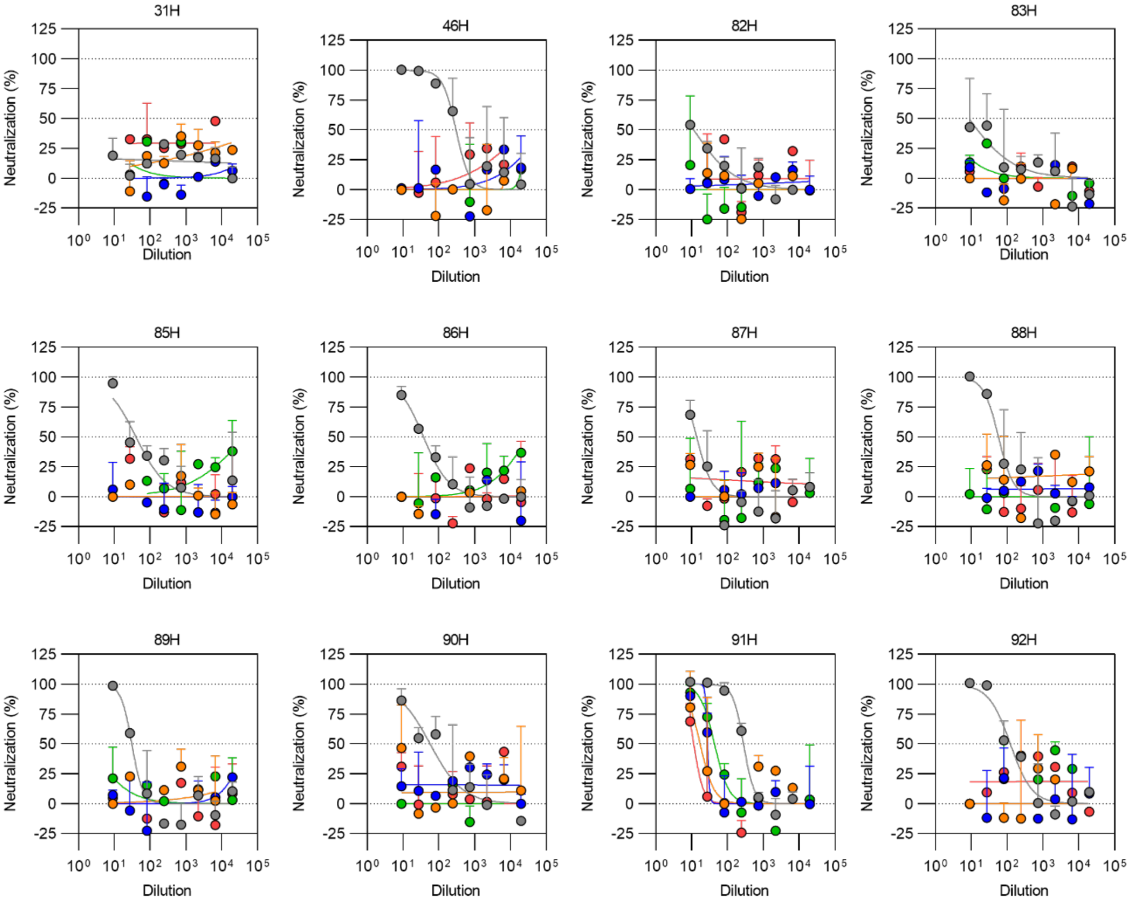

F 2x AZD1222

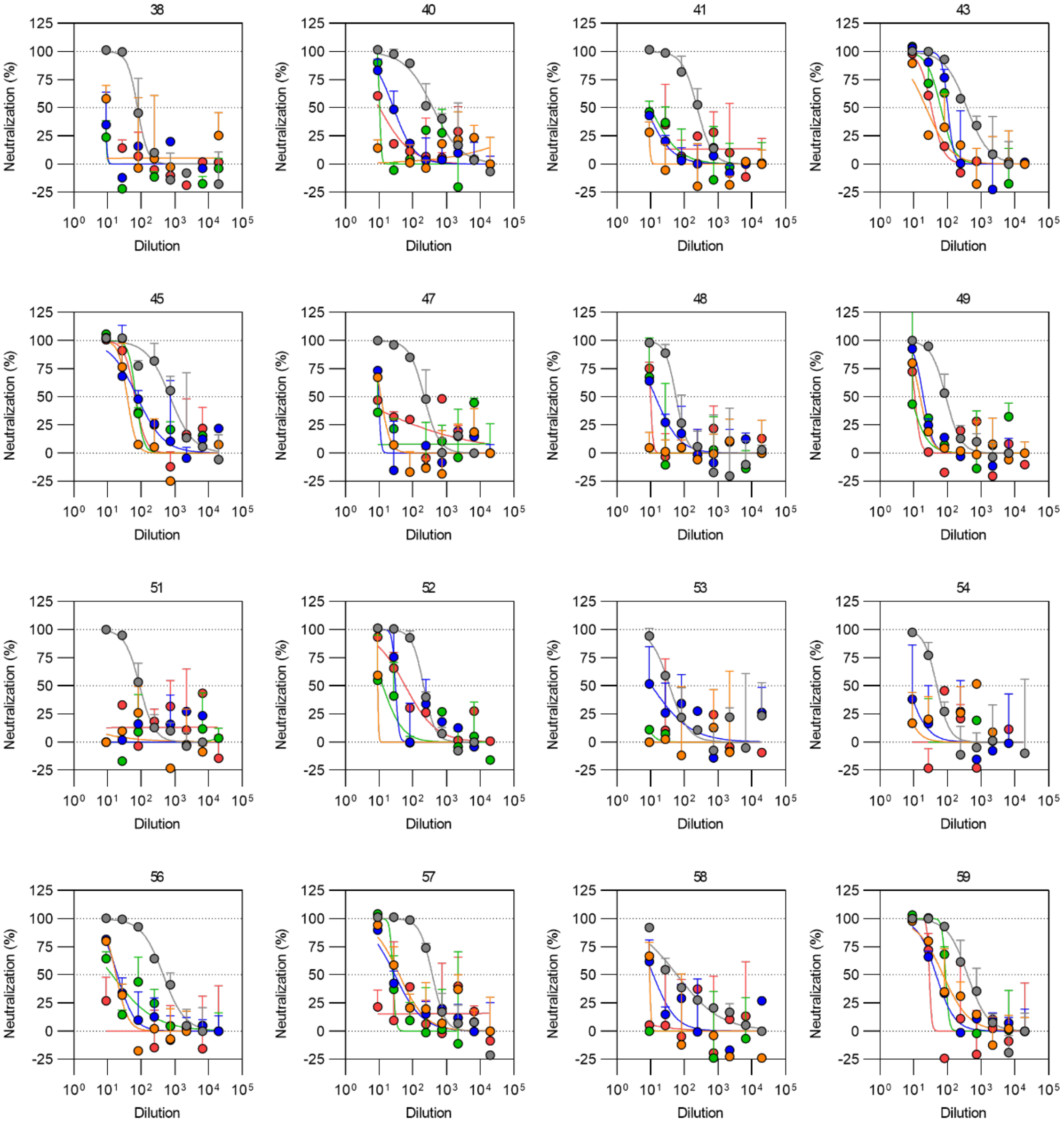
38
40
41
43
45
47
48
49
51
52
53
54
56
57
58
59
Neutralization (%)
Dilution
125
100
75
50
25
0
-25
10^0 10^1 10^2 10^3 10^4 10^5

G 2x Sputnik V

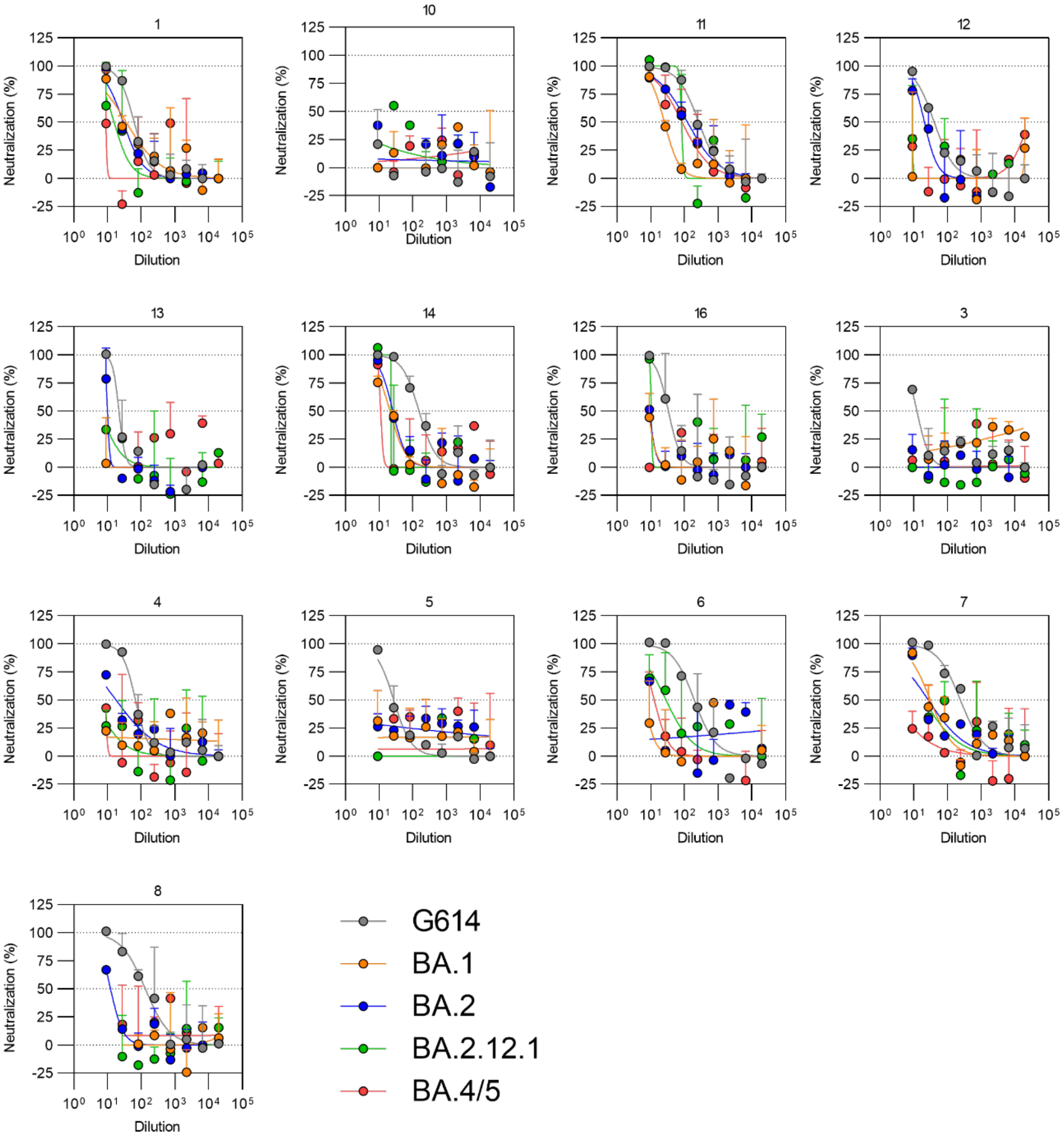

H 2x BBIBP-CorV

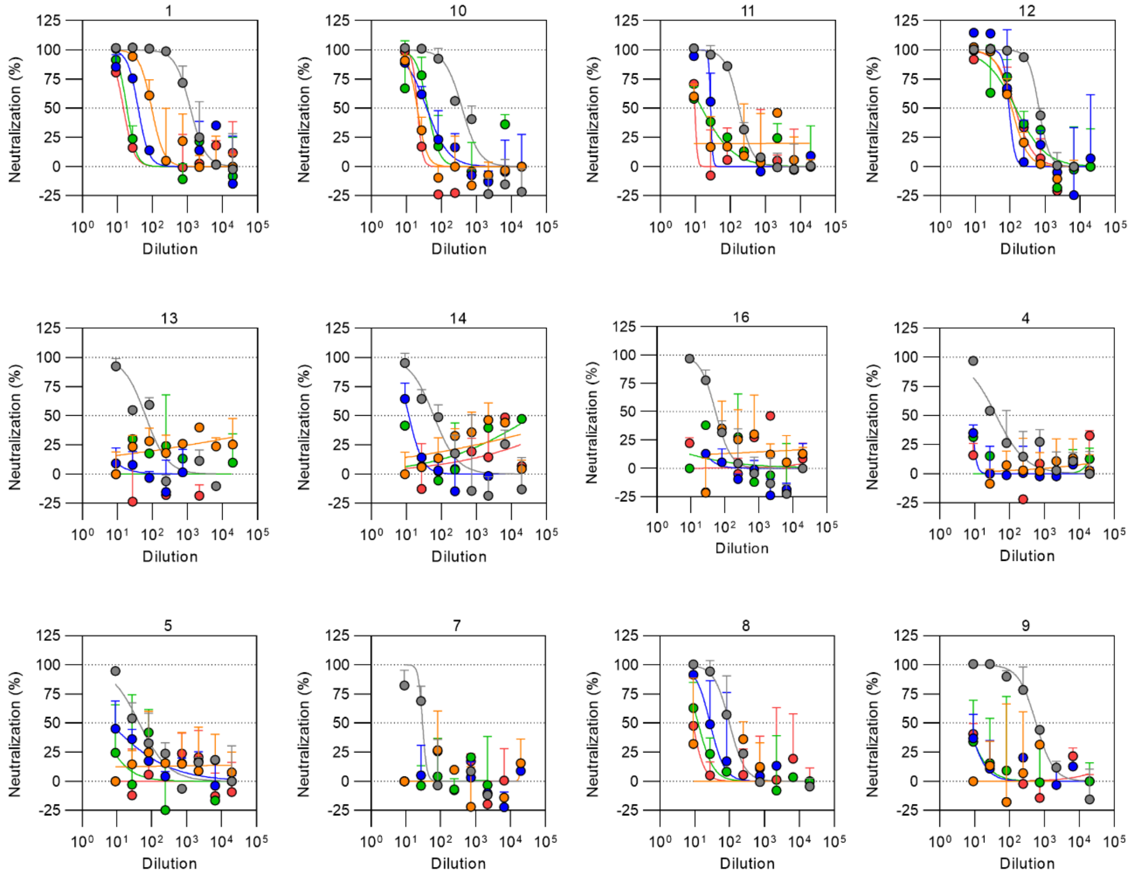

Fig. S5. Normalized neutralization curves using VSV pseudovirus harboring SARS-CoV-2 S with the D614G, BA.1, BA.2, BA.2.12.1, or BA.4/5 mutations using VeroE6/TMPRSS2 target cells and plasma from subjects previously infected (A) or administered with a primary vaccine series (B-H).

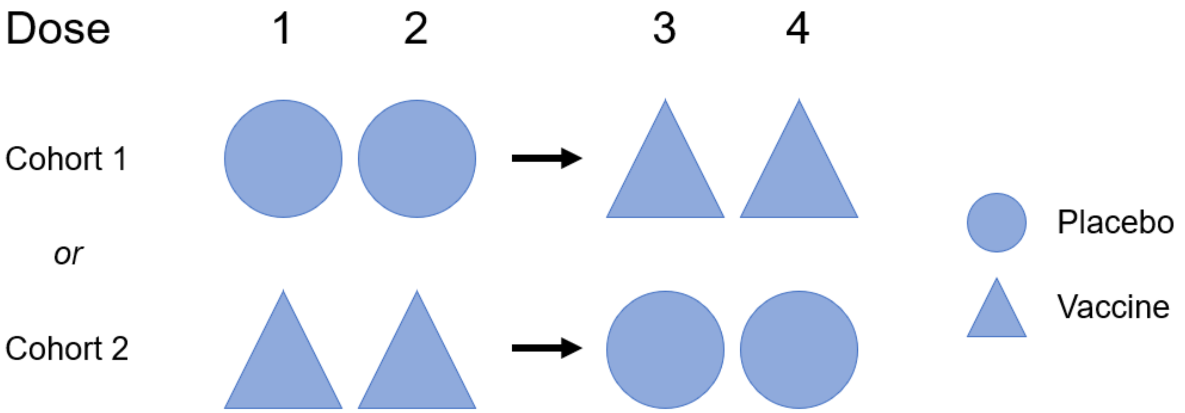

Fig. S6. Double blinded immunization scheme for individuals vaccinated with two doses of NVX-CoV2373. Each subject received two doses of NVX-CoV2373 (either at time points 1 and 2 or at time points 3 and 4) and two doses of placebo (at the other two time points). Each individual thus

received a total of two doses of NVX-CoV2373 as part of their primary vaccine series, although the time since vaccination is unknown for some donors (as a result of study double blinding).

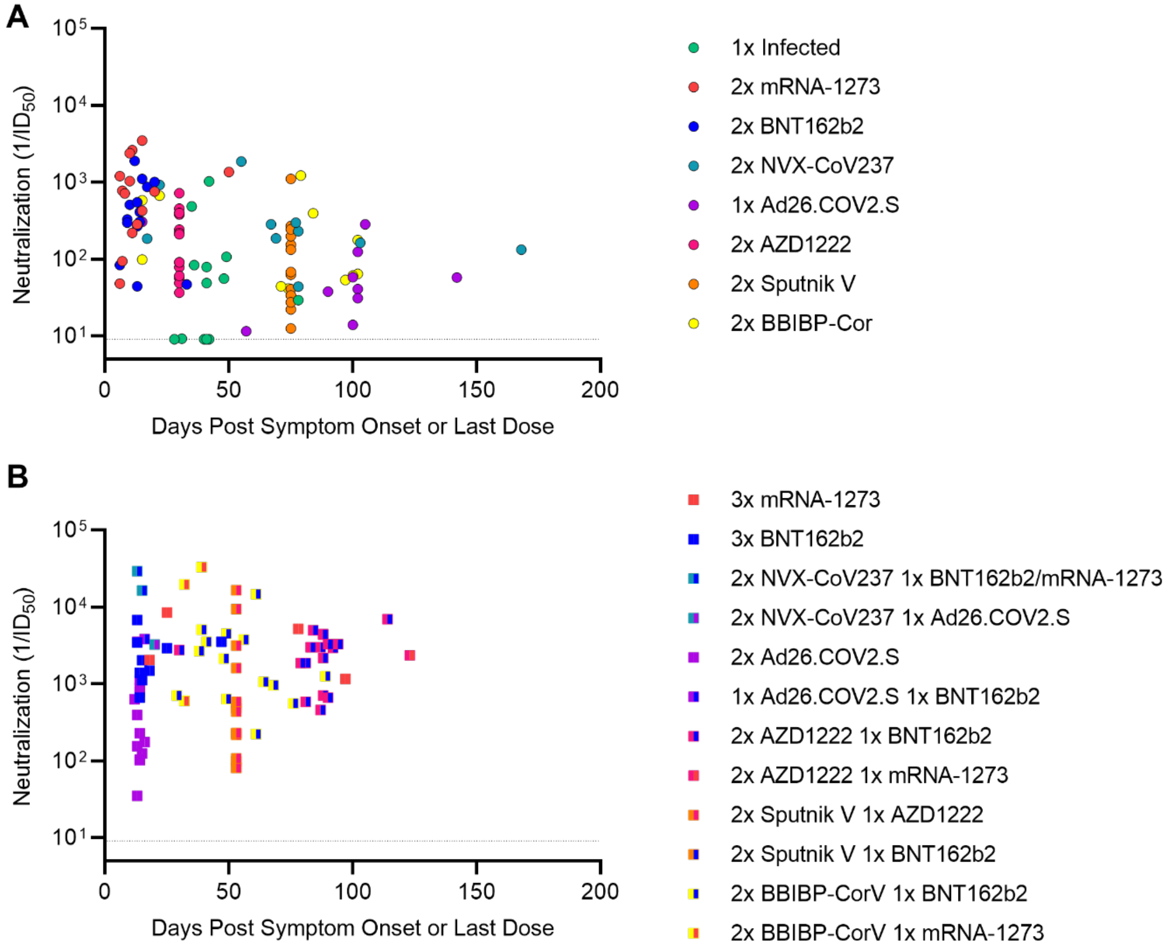

Fig. S7. Plasma neutralizing activity against SARS-CoV-2 G614 VSV pseudovirus plotted against the number days since symptoms onset (for 1x infected subjects) or since administration of the last vaccine dose (for all other samples) following primary vaccine series (A) or booster dose (B).

A 3x mRNA-1273 / BNT162b2

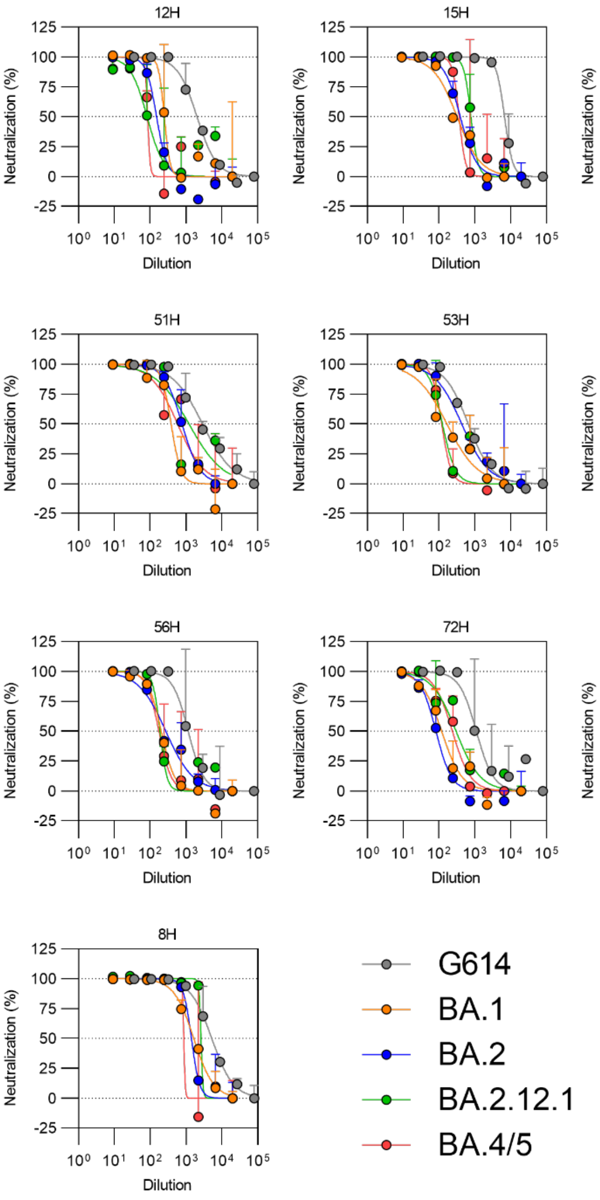

B 2x NVX-CoV2373 1x mRNA-1273/NVX-CoV2373

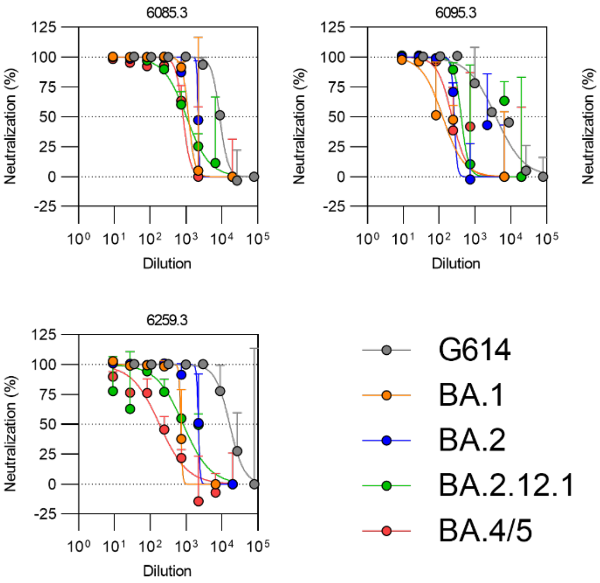

C 2x Ad26.COV2.S or 1x Ad26.COV2.S 1x BNT162b2

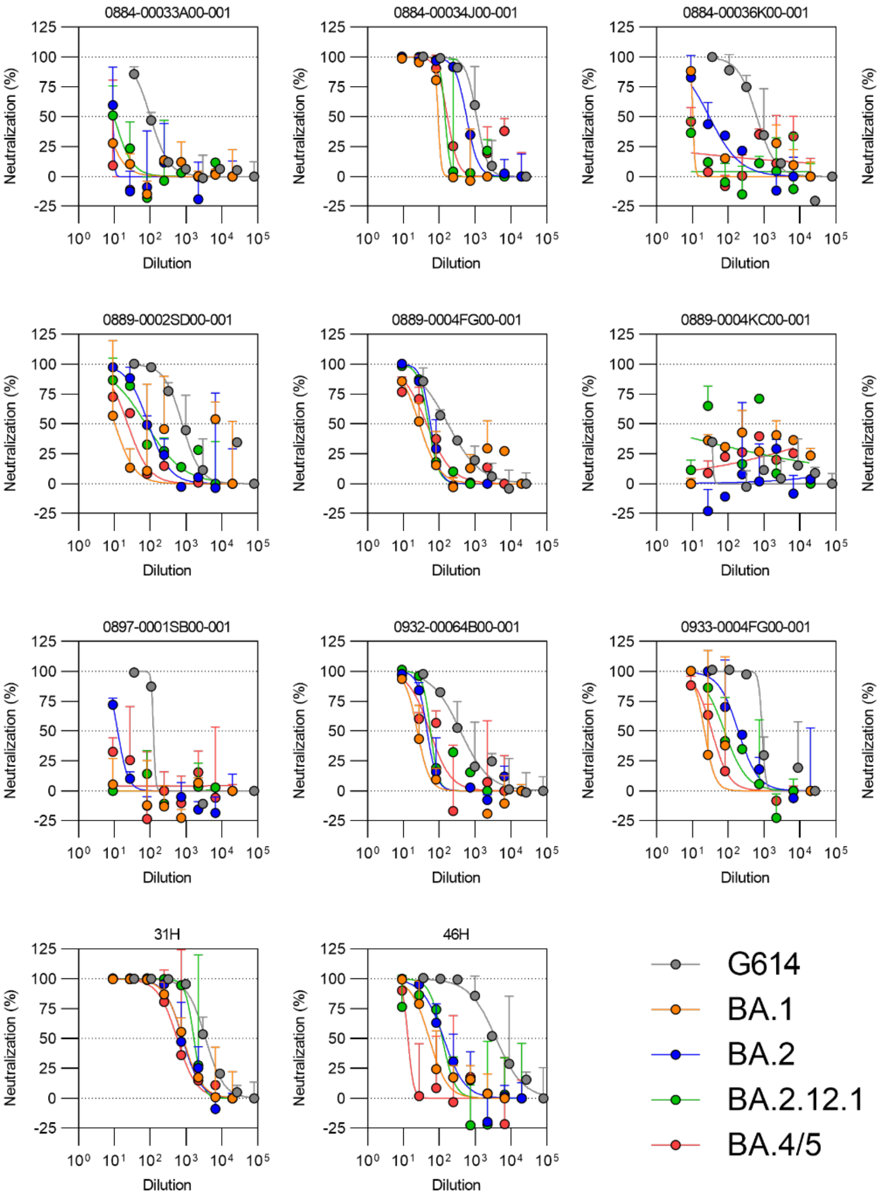

D 2x AZD1222 1x BNT162b2/mRNA-1273

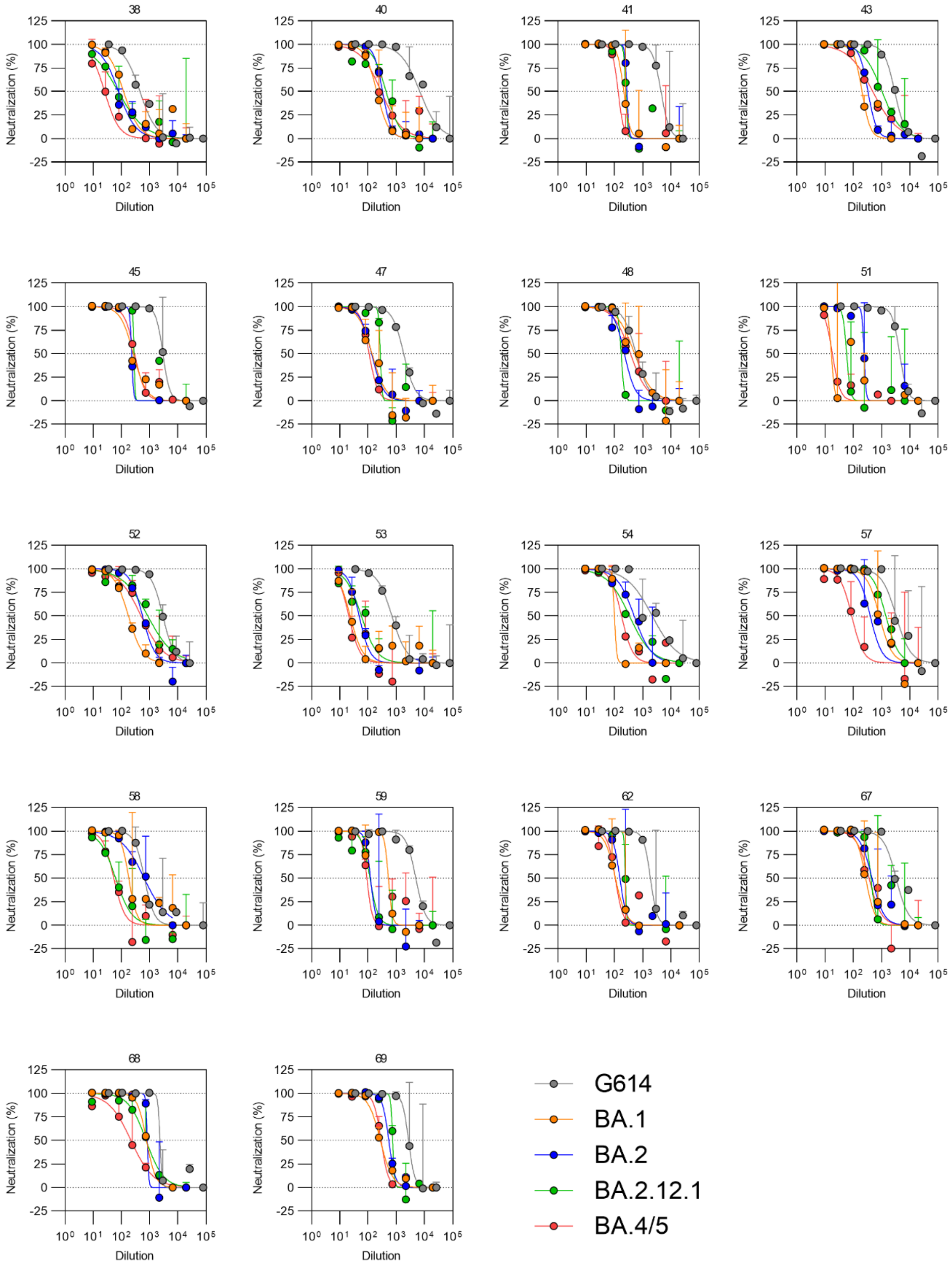

E 2x Sputnik V 1x AZD1222/BNT162b2

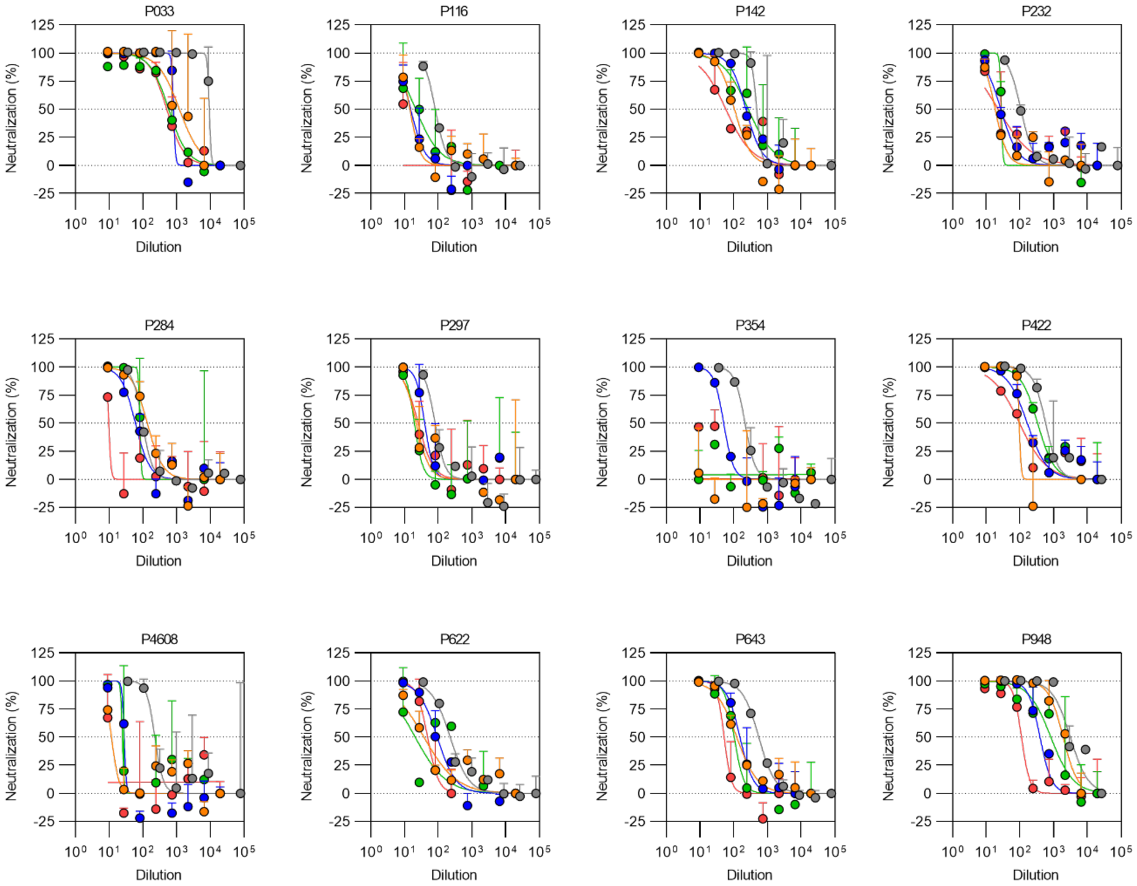

F 2x BBIBP-CorV 1x BNT162b2/mRNA-1273

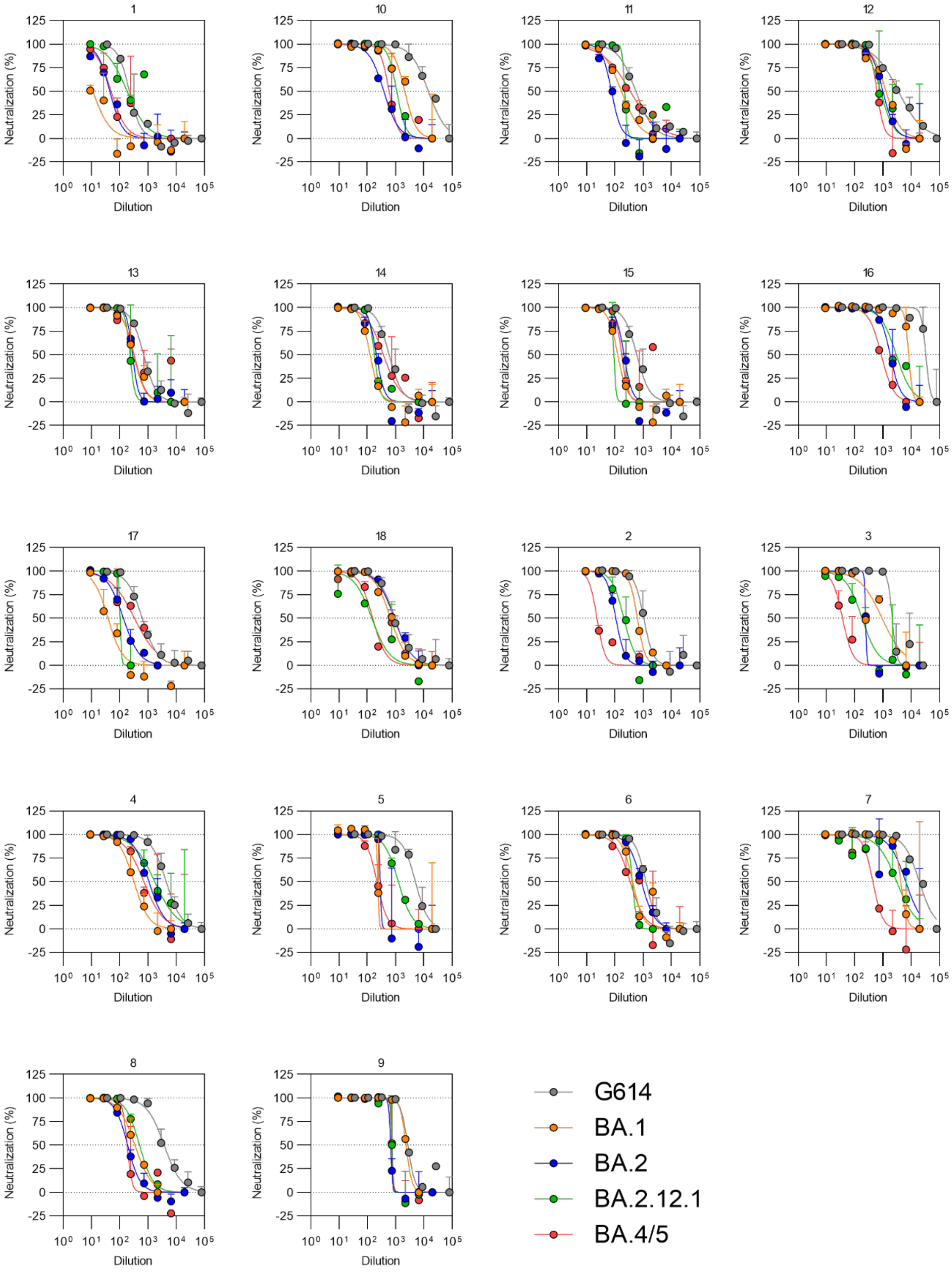

Fig. S8. Normalized neutralization curves using VSV pseudovirus containing the SARS-CoV-2 Wuhan-Hu-1/G614, BA.1, BA.2, BA.2.12.1, BA.4/5 S on VeroE6/TMPRSS2 cells using plasma from subjects that received a booster dose.

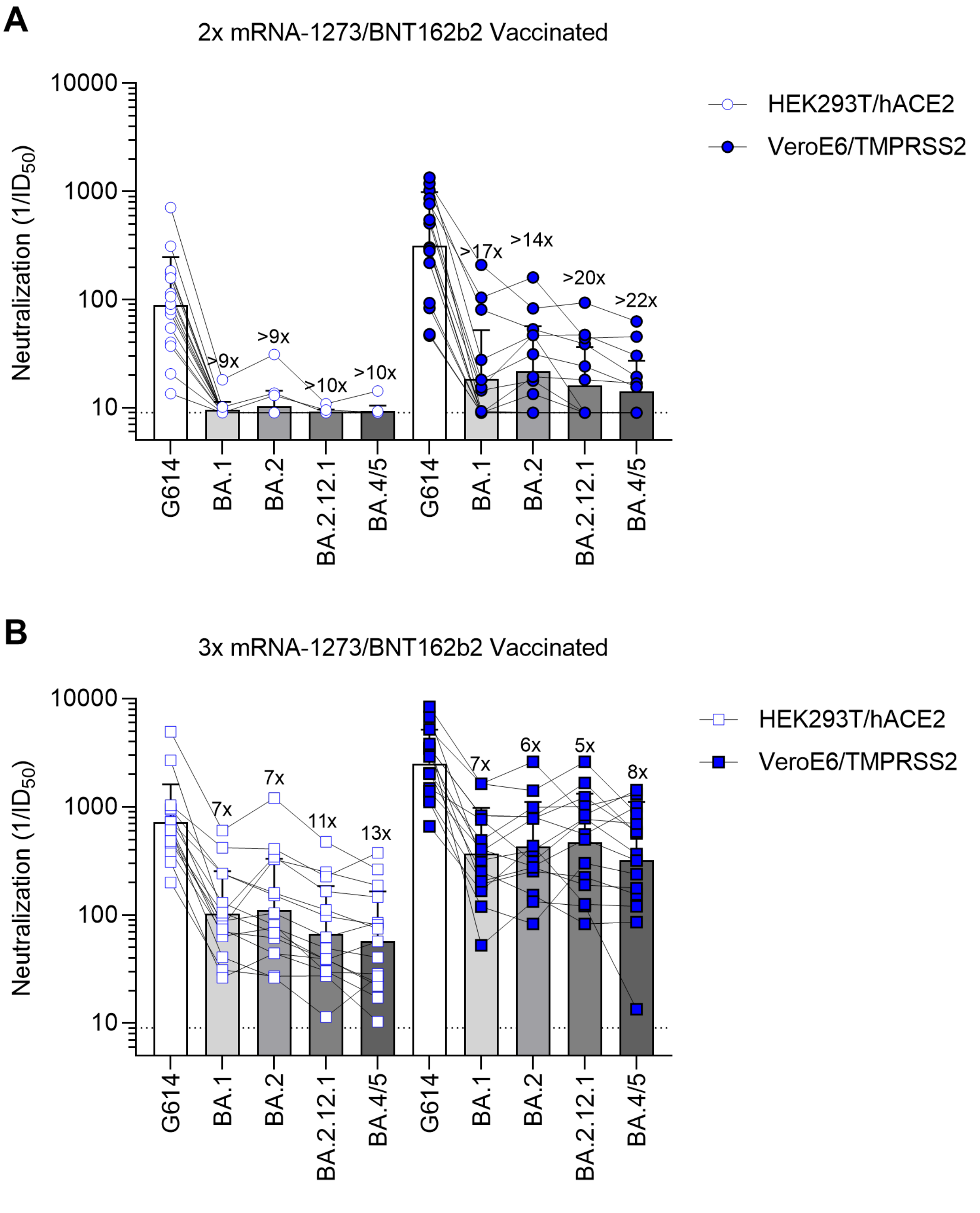

Fig. S9. Plasma neutralizing antibody titers elicited by COVID-19 vaccine boosters determined using SARS-CoV-2 S VSV pseudotypes and VeroE6/TMPRSS2 or HEK293T stably expressing human ACE2 (HEK293T/ACE2) as target cells. Individual points are representative geometric mean titers from two independent experiments consisting of two replicates each. Bars represent geometric means and error bars represent geometric standard deviations for each group. Patient

demographics are shown in Table S4. Normalized curves and fits are shown in Figure S10. G614: Wuhan-Hu-1/G614.

A 2x mRNA-1273 / BNT162b2

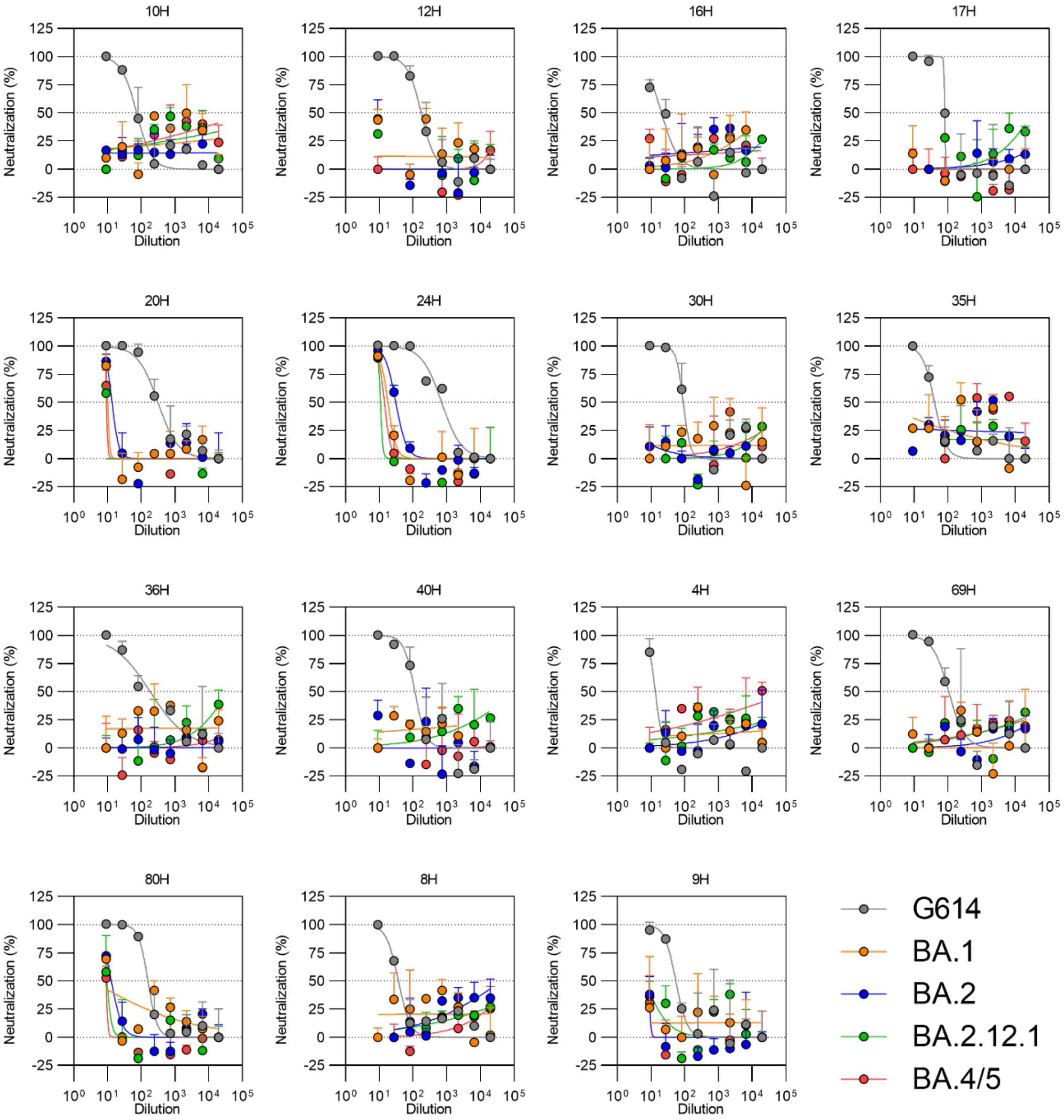

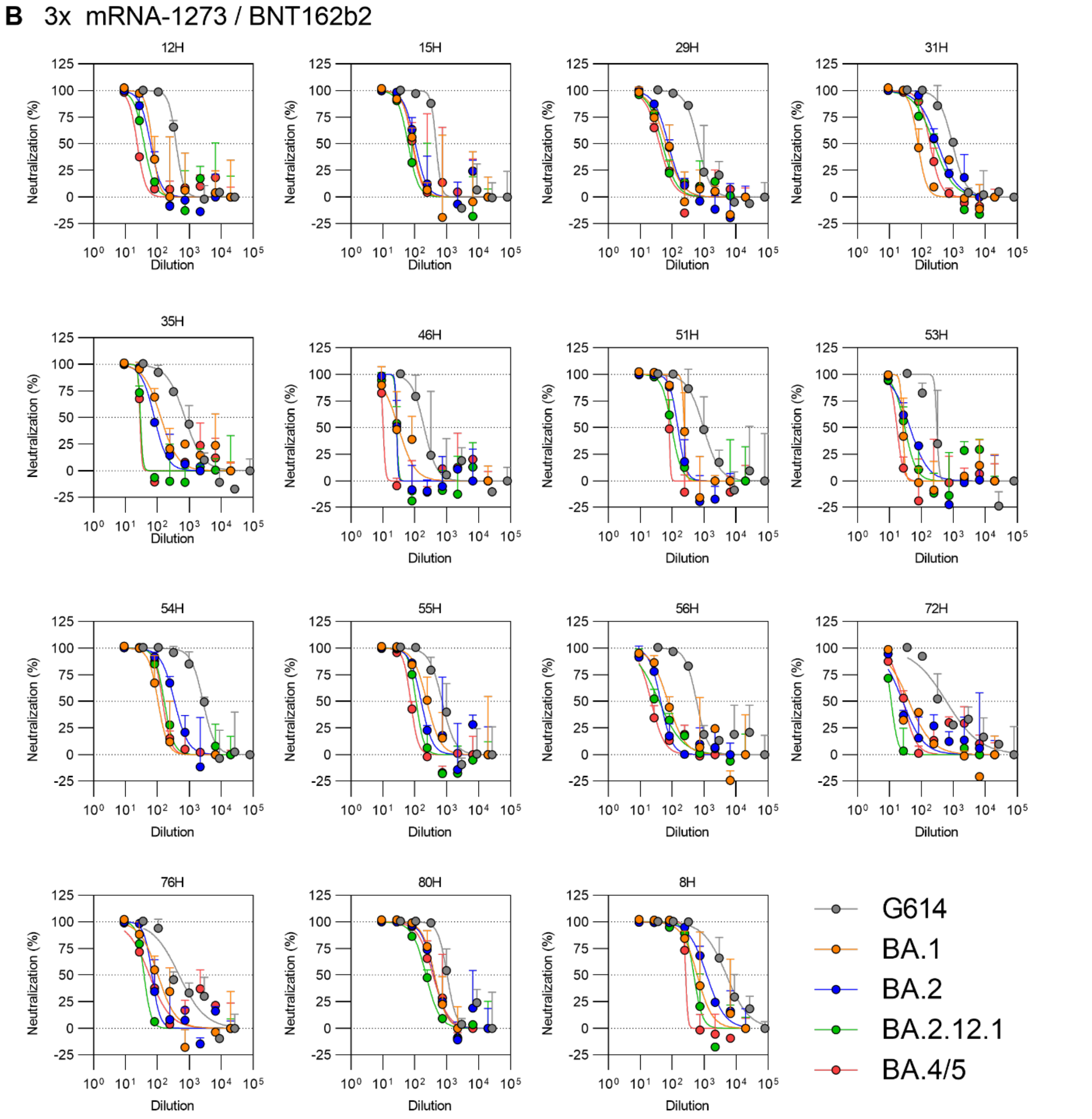

Fig. S10. Normalized neutralization curves using VSV pseudotyped with the SARS-CoV-2 S harboring the G614, BA.1, BA.2, BA.2.12.1, or BA.4/5 mutations with HEK293T/ACE2 target cells using plasma from mRNA-vaccinated subjects after their (A) primary series or (B) booster dose.

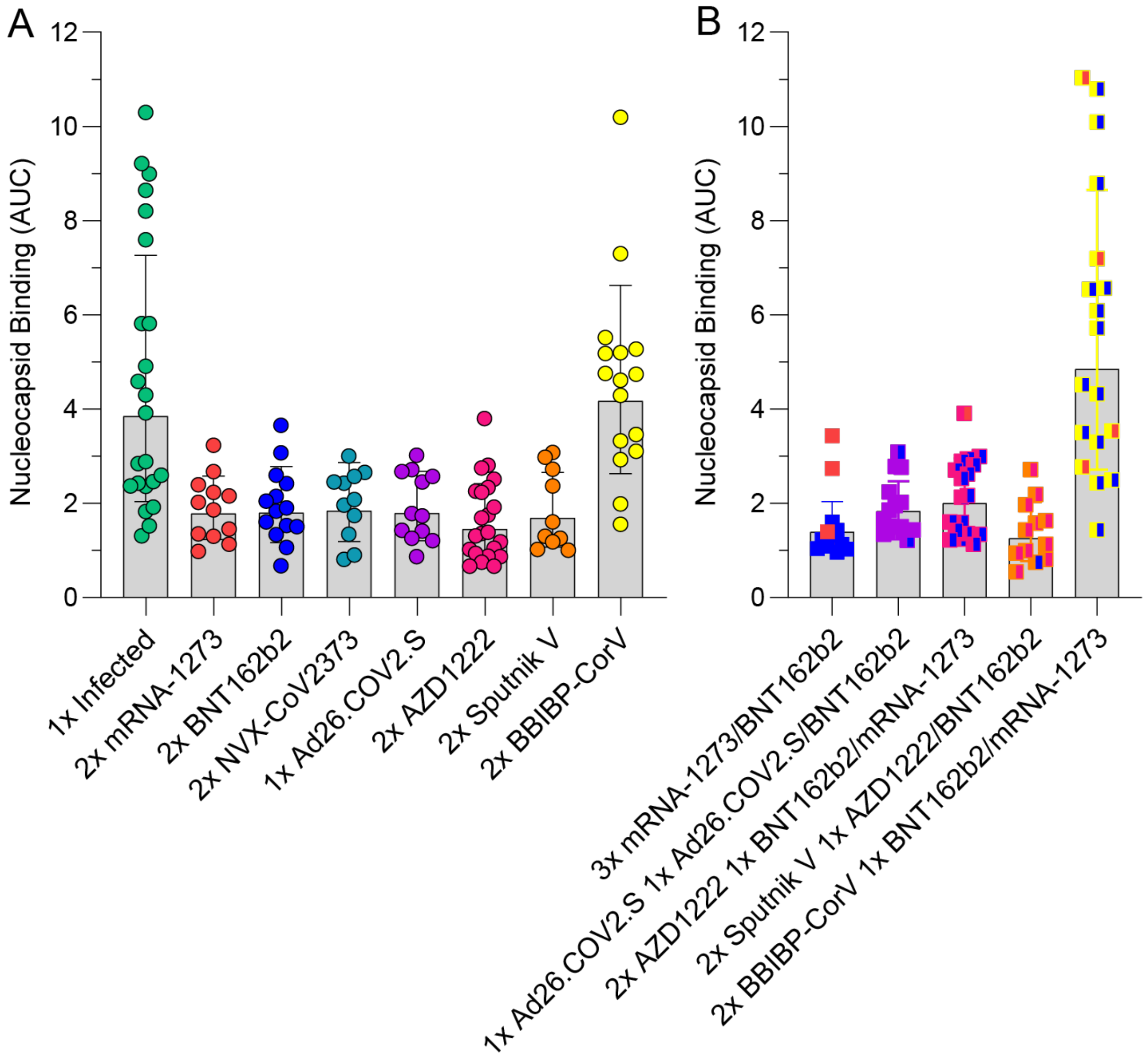

Fig. S11. Area under curve of log transformed SARS-CoV-2 nucleocapsid antibody binding titers of plasma samples obtained after primary vaccine series (A) or booster dose (B).

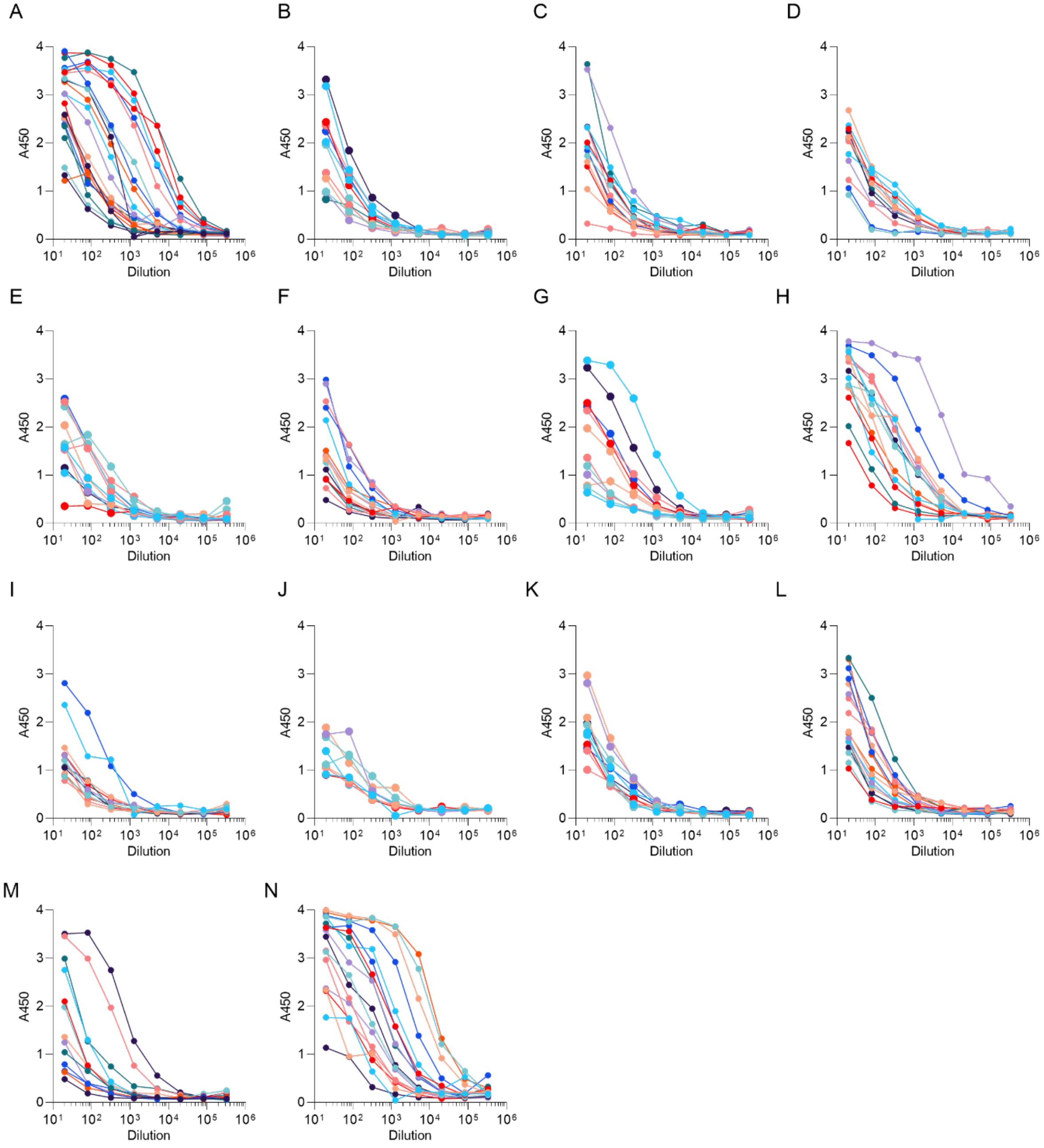

Fig. S12. Raw ELISA curves for plasma binding to the SARS-CoV-2 nucleoprotein following infection (A) or administration of two doses of mRNA-1273 (B), two doses of BNT162b2 (C), two doses of NVX-CoV2373 (D), one dose of Ad26.COV2.S (E), two doses of AZD1222 (F), two doses of Sputnik V (G), two doses of BBIBP-CorV (H), three doses of mRNA-1273 or BNT162b2 (I), two doses of NXV-CoV2373 followed by one dose of BNT162b2 or mRNA-1273 (J), two doses of Ad26.COV2.S (K), two doses of AZD1222 followed by one dose of BNT162b2 or mRNA-1273 (L), two doses of Sputnik V followed by one dose of AZD1222 or BNT162b2 (M), or two doses of BBIBP-CorV followed by one dose of BNT162b2 or mRNA-1273 (N).

Table S1. Kinetics of human ACE2 binding to immobilized SARS-CoV-2 RBDs determined by biolayer interferometry. Values are presented as mean ± standard error.

	K_D (nM)	k_{on} ($M^{-1}s^{-1}$)	k_{off} (s^{-1})
**Wuhan-Hu-1**	88.7 ± 2.4	1.6×10^5	1.4×10^{-2}
**Delta**	78.2 ± 0.4	1.2×10^5	9.2×10^{-3}
**BA.1**	19.7 ± 1.1	2.2×10^5	4.4×10^{-3}
**BA.2**	23.9 ± 2.1	1.6×10^5	3.8×10^{-3}
**BA.2.12.1**	81.6 ± 6.5	8.7×10^5	6.9×10^{-3}
**BA.4/5**	14.4 ± 0.3	1.4×10^5	2.0×10^{-3}

Table S2. Kinetics of human ACE2 binding to immobilized SARS-CoV-2 RBDs determined by SPR binding assays. K_D, k_{on}, and k_{off} values are reported as the average with the standard deviation from at least 4 replicates.

**ACE-2 binding**					
**RBD**	**K_D (nM)**	**k_{on} (1/Ms)**	**stdev (k_{on})**	**k_{off} (1/s)**	**stdev (k_{off})**
Wuhan-Hu-1	87.00 ± 10.80	7.11E+04	7.71E+03	6.12E-03	1.41E-04
Delta	65.66 ± 5.01	5.78E+04	1.84E+03	3.79E-03	2.09E-04
BA.1	36.94 ± 4.58	5.30E+04	5.70E+03	1.97E-03	3.61E-04
BA.2	44.96 ± 3.23	4.31E+04	1.06E+03	1.94E-03	1.32E-04
BA.2.12	52.56 ± 3.36	4.39E+04	2.31E+03	2.30E-03	4.07E-05
BA.4/5	28.06 ± 3.15	3.52E+04	1.34E+03	9.85E-04	1.01E-04

Table S3. SARS-CoV-2 Omicron S mutations as compared to Wuhan-Hu-1.

**BA.1**	**BA.2**	**BA.2.12.1**	**BA.4/5**
A67V, del69/70, T95I, G142D, del143/145, del211, L212I, ins214EPE, G339D, S371L, S373P, S375F, K417N, N440K, G446S, S477N, T478K, E484A, Q493R, G496S, Q498R, N501Y, Y505H, T547K, D614G, H655Y, N679K, P681H, N764K, D796Y, N856K, Q954H, N969K L981F	T19I, L24S, del25/27, G142D, V213G, G339D, S371F, S373P, S375F, T376A, D405N, R408S, K417N, N440K, S477N, T478K, E484A, Q493R, Q498R, N501Y, Y505H, D614G, H655Y, N679K, P681H, N764K, D796Y, Q954H, N969K	T19I, L24S, del25/27, G142D, V213G, G339D, S371F, S373P, S375F, T376A, D405N, R408S, K417N, N440K, L452Q, S477N, T478K, E484A, Q493R, Q498R, N501Y, Y505H, D614G, H655Y, N679K, P681H, S704L, N764K, D796Y, Q954H, N969K	T19I, L24S, del25/27, del69/70, G142D, V213G, G339D, S371F, S373P, S375F, T376A, D405N, R408S, K417N, N440K, L452R, S477N, T478K, E484A, F486V, Q498R, N501Y, Y505H, D614G, H655Y, N679K, P681H, N764K, D796Y, Q954H, N969K

Table S4. Demographics data of enrolled plasma donors.

Vaccine	Sample	Age	Sex	Days since symptoms onset or last vaccine dose	Vaccine	Sample	Age	Sex	Days Since Last Dose
1x Infected	10C	31	F	31	2x BBIBP-CorV	1	47	F	79
	114C	54	F	41		10	27	M	84
	13	54	M	32		11	28	F	102
	135C	43	M	49		12	36	M	22
	144C	30	M	48		13	26	F	102
	149C	25	M	40		14	26	F	100
	151	67	M	-		15	32	F	104
	151C	69	F	41		16	39	M	97
	157	42	F	-		2	36	M	78
	182C	75	M	78		3	41	F	9
	200C	61	M	42		4	25	F	74
	21C	36	F	26		5	26	F	71
	235C	65	M	31		6	34	F	82
	244C	57	F	68		7	29	M	76
	26C	25	F	28		8	34	F	15
	28C	64	F	42		9	33	F	15
	30C	60	F	35	3x mRNA-1273/BNT162b2	8H	79	M	78
	32C	36	M	30		12H	42	F	18
	54C	37	M	36		15H	52	M	13
	57C	56	F	35		29H	64	M	13
	65C	73	M	42		35H	60	M	47
	73C	60	M	49		51H	50	F	25
	98C	33	M	38		53H	38	F	14
	9C	37	F	41		54H	34	F	15
2x mRNA-1273	12H	42	F	10		55H	52	F	18
	136040	40	M	20		56H	46	M	97
	20H	33	F	6		72H	35	F	15
	24H	24	M	7		76H	38	F	14
	27H	59	M	11		80H	35	F	25
	38H	26	F	10	2x NVX-CoV2373<br>1x mRNA-1273	6085.2	36	M	20
	4H	23	M	6	3x NVX-CoV2373	6095.3	56	F	14
	56H	46	M	15		6096.3	18	M	14
	58H	50	F	15		6097.3	18	M	14
	60H	42	F	8		6259.3	42	F	15
	69H	68	F	13	2x Ad26.COV2.S	0884-00033A00-001	38	M	14
	80H	35	F	50		0884-00034J00-001	72	F	14
	8H	79	M	11		0884-00036K00-001	43	F	12
	9H	75	F	7		0884-0003WD00-001	54	M	13
2x BNT162b2	10H	33	M	14		0889-0002SD00-001	43	M	14
	15H	52	M	9		0889-0004FG00-001	28	F	16
	16H	46	F	6		0889-0004KC00-001	36	F	13
	17H	76	M	17		0889-0004WJ00-001	48	F	14
	29H	64	M	12		0897-0001SB00-001	41	F	15
	30H	61	F	10		0932-00064B00-001	60	F	13

	35H	60	M	33		0933-0004FG00-001	51	M	14
	36H	37	M	9		0933-0004KC00-001	26	F	14
	40H	38	F	13	1x Ad26.COV.2.S 1x BNT162b2	31H	49	F	13
	41H	36	F	13		46H	60	M	16
	67H	40	F	13	2x AZD1222 1x BNT162b2	38	30	F	87
	77H	28	F	14		40	52	F	114
	78H	22	M	15		41	40	F	88
	79H	27	F	20		43	48	M	92
2x NVX-CoV2373	6082.2	M	21	103		45	49	F	83
	6085.1	M	36	17 or 92		47	32	F	81
	6090.2	F	36	55		48	38	F	81
	6094.1	F	46	22 or 127		51	30	F	88
	6096.2	M	18	155		52	47	M	87
	6097.2	M	18	78		53	38	F	88
	6099.2	M	20	69 or 156		54	30	F	88
	6259.1	F	42	172		57	46	F	94
	6261.1	M	60	191		58	29	F	90
	6322.2	F	41	168		59	48	M	84
1x Ad26.COV2.S	31H	49	F	9		62	41	F	79
	46H	60	M	15		67	42	F	90
	82H	28	M	57		69	24	M	30
	83H	23	M	24	2x AZD1222 1x mRNA-1273	68	26	M	123
	85H	29	M	102	2x Sputnik V 1x BNT162b2	P643	61	F	45-60
	86H	26	F	90	2x Sputnik V 1x AZD1222	P142	45	F	45-60
	87H	28	F	100		P297	47	F	45-60
	88H	30	F	100		P284	64	M	45-60
	89H	31	M	102		P354	61	M	45-60
	90H	38	M	142		P116	47	F	45-60
	91H	26	F	105		P948	38	F	45-60
	92H	33	F	102		P033	54	M	45-60
2x AZD1222	38	29	F	30		P622	60	M	45-60
	40	51	F	30		P232	58	M	45-60
	41	39	F	30		P4608	33	F	45-60
	43	47	M	30		P422	45	F	45-60
	45	48	F	30	2x BBIBP-CorV 1x BNT162b2	1	27	F	61
	47	31	F	30		10	32	M	61
	48	37	F	30		11	35	M	76
	49	36	F	30		12	62	M	41
	51	29	F	30		13	17	M	29
	52	46	M	30		17	34	M	49
	53	37	F	30		18	27	M	68
	54	29	F	30		2	37	M	64
	56	45	F	30		3	33	F	48
	57	45	F	30		4	30	F	49
	58	28	F	30		5	29	M	39
	59	47	M	30		6	30	M	89
2x Sputnik V	1	-	-	60-90		8	36	M	56
	10	-	-	60-90		9	34	M	38
	11	-	-	60-90	2x BBIBP-CorV 1x mRNA-1273	7	24	F	32
	12	-	-	60-90		14	30	M	32
	13	-	-	60-90		15	27	F	32

	14	-	-	60-90		16	28	M	39
	16	-	-	60-90					
	3	-	-	60-90					
	4	-	-	60-90					
	5	-	-	60-90					
	6	-	-	60-90					
	7	-	-	60-90					
	8	-	-	60-90					

References and Notes

1. M. McCallum, J. Bassi, A. De Marco, A. Chen, A. C. Walls, J. Di Iulio, M. A. Tortorici, M.-J. Navarro, C. Silacci-Fregni, C. Saliba, K. R. Sprouse, M. Agostini, D. Pinto, K. Culap, S. Bianchi, S. Jaconi, E. Cameroni, J. E. Bowen, S. W. Tilles, M. S. Pizzuto, S. B. Guastalla, G. Bona, A. F. Pellanda, C. Garzoni, W. C. Van Voorhis, L. E. Rosen, G. Snell, A. Telenti, H. W. Virgin, L. Piccoli, D. Corti, D. Veesler, SARS-CoV-2 immune evasion by the B.1.427/B.1.429 variant of concern. *Science* **373**, 648–654 (2021). doi:10.1126/science.abi7994 Medline

2. E. B. Hodcroft, M. Zuber, S. Nadeau, T. G. Vaughan, K. H. D. Crawford, C. L. Althaus, M. L. Reichmuth, J. E. Bowen, A. C. Walls, D. Corti, J. D. Bloom, D. Veesler, D. Mateo, A. Hernando, I. Comas, F. González-Candelas, SeqCOVID-SPAIN consortium, T. Stadler, R. A. Neher, Spread of a SARS-CoV-2 variant through Europe in the summer of 2020. *Nature* **595**, 707–712 (2021). doi:10.1038/s41586-021-03677-y Medline

3. M. McCallum, A. C. Walls, K. R. Sprouse, J. E. Bowen, L. E. Rosen, H. V. Dang, A. De Marco, N. Franko, S. W. Tilles, J. Logue, M. C. Miranda, M. Ahlrichs, L. Carter, G. Snell, M. S. Pizzuto, H. Y. Chu, W. C. Van Voorhis, D. Corti, D. Veesler, Molecular basis of immune evasion by the Delta and Kappa SARS-CoV-2 variants. *Science* **374**, 1621–1626 (2021). doi:10.1126/science.abl8506 Medline

4. M. McCallum, N. Czudnochowski, L. E. Rosen, S. K. Zepeda, J. E. Bowen, A. C. Walls, K. Hauser, A. Joshi, C. Stewart, J. R. Dillen, A. E. Powell, T. I. Croll, J. Nix, H. W. Virgin, D. Corti, G. Snell, D. Veesler, Structural basis of SARS-CoV-2 Omicron immune evasion and receptor engagement. *Science* **375**, 864–868 (2022). doi:10.1126/science.abn8652 Medline

5. A. C. Walls, K. R. Sprouse, J. E. Bowen, A. Joshi, N. Franko, M. J. Navarro, C. Stewart, E. Cameroni, M. McCallum, E. A. Goecker, E. J. Degli-Angeli, J. Logue, A. Greninger, D. Corti, H. Y. Chu, D. Veesler, SARS-CoV-2 breakthrough infections elicit potent, broad, and durable neutralizing antibody responses. *Cell* **185**, 872–880.e3 (2022). doi:10.1016/j.cell.2022.01.011 Medline

6. S. Cele, L. Jackson, D. S. Khoury, K. Khan, T. Moyo-Gwete, H. Tegally, J. E. San, D. Cromer, C. Scheepers, D. G. Amoako, F. Karim, M. Bernstein, G. Lustig, D. Archary, M. Smith, Y. Ganga, Z. Jule, K. Reedoy, S.-H. Hwa, J. Giandhari, J. M. Blackburn, B. I. Gosnell, S. S. Abdool Karim, W. Hanekom, NGS-SA, COMMIT-KZN Team, A. von Gottberg, J. N. Bhiman, R. J. Lessells, M.-Y. S. Moosa, M. P. Davenport, T. de Oliveira, P. L. Moore, A. Sigal, Omicron extensively but incompletely escapes Pfizer BNT162b2 neutralization. *Nature* **602**, 654–656 (2022). doi:10.1038/s41586-021-04387-1 Medline

7. R. Viana, S. Moyo, D. G. Amoako, H. Tegally, C. Scheepers, C. L. Althaus, U. J. Anyaneji, P. A. Bester, M. F. Boni, M. Chand, W. T. Choga, R. Colquhoun, M. Davids, K. Deforche, D. Doolabh, L. du Plessis, S. Engelbrecht, J. Everatt, J. Giandhari, M. Giovanetti, D. Hardie, V. Hill, N.-Y. Hsiao, A. Iranzadeh, A. Ismail, C. Joseph, R. Joseph, L. Koopile, S. L. Kosakovsky Pond, M. U. G. Kraemer, L. Kuate-Lere, O. Laguda-Akingba, O. Lesetedi-Mafoko, R. J. Lessells, S. Lockman, A. G. Lucaci, A. Maharaj, B. Mahlangu, T. Maponga, K. Mahlakwane, Z. Makatini, G. Marais, D. Maruapula, K. Masupu, M. Matshaba, S. Mayaphi, N. Mbhele, M. B. Mbulawa, A. Mendes, K. Mlisana, A. Mnguni,

T. Mohale, M. Moir, K. Moruisi, M. Mosepele, G. Motsatsi, M. S. Motswaledi, T. Mphoyakgosi, N. Msomi, P. N. Mwangi, Y. Naidoo, N. Ntuli, M. Nyaga, L. Olubayo, S. Pillay, B. Radibe, Y. Ramphal, U. Ramphal, J. E. San, L. Scott, R. Shapiro, L. Singh, P. Smith-Lawrence, W. Stevens, A. Strydom, K. Subramoney, N. Tebeila, D. Tshiabuila, J. Tsui, S. van Wyk, S. Weaver, C. K. Wibmer, E. Wilkinson, N. Wolter, A. E. Zarebski, B. Zuze, D. Goedhals, W. Preiser, F. Treurnicht, M. Venter, C. Williamson, O. G. Pybus, J. Bhiman, A. Glass, D. P. Martin, A. Rambaut, S. Gaseitsiwe, A. von Gottberg, T. de Oliveira, Rapid epidemic expansion of the SARS-CoV-2 Omicron variant in southern Africa. *Nature* **603**, 679–686 (2022). doi:10.1038/s41586-022-04411-y Medline

8. H. Tegally, E. Wilkinson, M. Giovanetti, A. Iranzadeh, V. Fonseca, J. Giandhari, D. Doolabh, S. Pillay, E. J. San, N. Msomi, K. Mlisana, A. von Gottberg, S. Walaza, M. Allam, A. Ismail, T. Mohale, A. J. Glass, S. Engelbrecht, G. Van Zyl, W. Preiser, F. Petruccione, A. Sigal, D. Hardie, G. Marais, N. Hsiao, S. Korsman, M.-A. Davies, L. Tyers, I. Mudau, D. York, C. Maslo, D. Goedhals, S. Abrahams, O. Laguda-Akingba, A. Alisoltani-Dehkordi, A. Godzik, C. K. Wibmer, B. T. Sewell, J. Lourenço, L. C. J. Alcantara, S. L. Kosakovsky Pond, S. Weaver, D. Martin, R. J. Lessells, J. N. Bhiman, C. Williamson, T. de Oliveira, Detection of a SARS-CoV-2 variant of concern in South Africa. *Nature* **592**, 438–443 (2021). doi:10.1038/s41586-021-03402-9 Medline

9. P. Mlcochova, S. A. Kemp, M. S. Dhar, G. Papa, B. Meng, I. A. T. M. Ferreira, R. Datir, D. A. Collier, A. Albecka, S. Singh, R. Pandey, J. Brown, J. Zhou, N. Goonawardane, S. Mishra, C. Whittaker, T. Mellan, R. Marwal, M. Datta, S. Sengupta, K. Ponnusamy, V. S. Radhakrishnan, A. Abdullahi, O. Charles, P. Chattopadhyay, P. Devi, D. Caputo, T. Peacock, C. Wattal, N. Goel, A. Satwik, R. Vaishya, M. Agarwal, Indian SARS-CoV-2 Genomics Consortium (INSACOG), Genotype to Phenotype Japan (G2P-Japan) Consortium, CITIID-NIHR BioResource COVID-19 Collaboration, A. Mavousian, J. H. Lee, J. Bassi, C. Silacci-Fegni, C. Saliba, D. Pinto, T. Irie, I. Yoshida, W. L. Hamilton, K. Sato, S. Bhatt, S. Flaxman, L. C. James, D. Corti, L. Piccoli, W. S. Barclay, P. Rakshit, A. Agrawal, R. K. Gupta, SARS-CoV-2 B.1.617.2 Delta variant replication and immune evasion. *Nature* **599**, 114–119 (2021). doi:10.1038/s41586-021-03944-y Medline

10. D. A. Collier, A. De Marco, I. A. T. M. Ferreira, B. Meng, R. P. Datir, A. C. Walls, S. A. Kemp, J. Bassi, D. Pinto, C. Silacci-Fregni, S. Bianchi, M. A. Tortorici, J. Bowen, K. Culap, S. Jaconi, E. Cameroni, G. Snell, M. S. Pizzuto, A. F. Pellanda, C. Garzoni, A. Riva, CITIID-NIHR BioResource COVID-19 Collaboration, A. Elmer, N. Kingston, B. Graves, L. E. McCoy, K. G. C. Smith, J. R. Bradley, N. Temperton, L. Ceron-Gutierrez, G. Barcenas-Morales, COVID-19 Genomics UK (COG-UK) Consortium, W. Harvey, H. W. Virgin, A. Lanzavecchia, L. Piccoli, R. Doffinger, M. Wills, D. Veesler, D. Corti, R. K. Gupta, Sensitivity of SARS-CoV-2 B.1.1.7 to mRNA vaccine-elicited antibodies. *Nature* **593**, 136–141 (2021). doi:10.1038/s41586-021-03412-7 Medline

11. A. Saito, T. Irie, R. Suzuki, T. Maemura, H. Nasser, K. Uriu, Y. Kosugi, K. Shirakawa, K. Sadamasu, I. Kimura, J. Ito, J. Wu, K. Iwatsuki-Horimoto, M. Ito, S. Yamayoshi, S. Loeber, M. Tsuda, L. Wang, S. Ozono, E. P. Butlertanaka, Y. L. Tanaka, R. Shimizu, K. Shimizu, K. Yoshimatsu, R. Kawabata, T. Sakaguchi, K. Tokunaga, I. Yoshida, H. Asakura, M. Nagashima, Y. Kazuma, R. Nomura, Y. Horisawa, K. Yoshimura, A. Takaori-Kondo, M. Imai, Genotype to Phenotype Japan (G2P-Japan) Consortium, S.

Tanaka, S. Nakagawa, T. Ikeda, T. Fukuhara, Y. Kawaoka, K. Sato, Enhanced fusogenicity and pathogenicity of SARS-CoV-2 Delta P681R mutation. *Nature* **602**, 300–306 (2022). doi:10.1038/s41586-021-04266-9 Medline

12. E. Cameroni, J. E. Bowen, L. E. Rosen, C. Saliba, S. K. Zepeda, K. Culap, D. Pinto, L. A. VanBlargan, A. De Marco, J. di Iulio, F. Zatta, H. Kaiser, J. Noack, N. Farhat, N. Czudnochowski, C. Havenar-Daughton, K. R. Sprouse, J. R. Dillen, A. E. Powell, A. Chen, C. Maher, L. Yin, D. Sun, L. Soriaga, J. Bassi, C. Silacci-Fregni, C. Gustafsson, N. M. Franko, J. Logue, N. T. Iqbal, I. Mazzitelli, J. Geffner, R. Grifantini, H. Chu, A. Gori, A. Riva, O. Giannini, A. Ceschi, P. Ferrari, P. E. Cippà, A. Franzetti-Pellanda, C. Garzoni, P. J. Halfmann, Y. Kawaoka, C. Hebner, L. A. Purcell, L. Piccoli, M. S. Pizzuto, A. C. Walls, M. S. Diamond, A. Telenti, H. W. Virgin, A. Lanzavecchia, G. Snell, D. Veesler, D. Corti, Broadly neutralizing antibodies overcome SARS-CoV-2 Omicron antigenic shift. *Nature* **602**, 664–670 (2022). doi:10.1038/s41586-021-04386-2 Medline

13. F. Schmidt, F. Muecksch, Y. Weisblum, J. Da Silva, E. Bednarski, A. Cho, Z. Wang, C. Gaebler, M. Caskey, M. C. Nussenzweig, T. Hatziioannou, P. D. Bieniasz, Plasma Neutralization of the SARS-CoV-2 Omicron Variant. *N. Engl. J. Med.* **386**, 599–601 (2022). doi:10.1056/NEJMc2119641 Medline

14. A. Wilhelm, M. Widera, K. Grikscheit, T. Toptan, B. Schenk, C. Pallas, M. Metzler, N. Kohmer, S. Hoehl, F. A. Helfritz, T. Wolf, U. Goetsch, S. Ciesek, Reduced neutralization of SARS-CoV-2 Omicron variant by vaccine Sera and monoclonal antibodies. medRxiv 2021.12.07.21267432 [Preprint] (2021). https://doi.org/10.1101/2021.12.07.21267432.

15. B. Meng, A. Abdullahi, I. A. T. M. Ferreira, N. Goonawardane, A. Saito, I. Kimura, D. Yamasoba, P. P. Gerber, S. Fatihi, S. Rathore, S. K. Zepeda, G. Papa, S. A. Kemp, T. Ikeda, M. Toyoda, T. S. Tan, J. Kuramochi, S. Mitsunaga, T. Ueno, K. Shirakawa, A. Takaori-Kondo, T. Brevini, D. L. Mallery, O. J. Charles, CITIID-NIHR BioResource COVID-19 Collaboration, Genotype to Phenotype Japan (GP-Japan) Consortium, Ecuador-COVID19 Consortium, J. E. Bowen, A. Joshi, A. C. Walls, L. Jackson, D. Martin, K. G. C. Smith, J. Bradley, J. A. G. Briggs, J. Choi, E. Madissoon, K. B. Meyer, P. Mlcochova, L. Ceron-Gutierrez, R. Doffinger, S. A. Teichmann, A. J. Fisher, M. S. Pizzuto, A. de Marco, D. Corti, M. Hosmillo, J. H. Lee, L. C. James, L. Thukral, D. Veesler, A. Sigal, F. Sampaziotis, I. G. Goodfellow, N. J. Matheson, K. Sato, R. K. Gupta, Altered TMPRSS2 usage by SARS-CoV-2 Omicron impacts infectivity and fusogenicity. *Nature* **603**, 706–714 (2022). doi:10.1038/s41586-022-04474-x Medline

16. H. Allen, E. Tessier, C. Turner, C. Anderson, P. Blomquist, D. Simons, A. Løchen, C. I. Jarvis, N. Groves, F. Capelastegui, J. Flannagan, A. Zaidi, C. Chen, C. Rawlinson, G. J. Hughes, D. Chudasama, S. Nash, S. Thelwall, J. Lopez-Bernal, G. Dabrera, A. Charlett, M. Kall, T. Lamagni, Comparative transmission of SARS-CoV-2 Omicron (B.1.1.529) and Delta (B.1.617.2) variants and the impact of vaccination: National cohort study, England. medRxiv 2022.02.15.22271001 [Preprint] (2022). https://doi.org/10.1101/2022.02.15.22271001.

17. W. Dejnirattisai, J. Huo, D. Zhou, J. Zahradník, P. Supasa, C. Liu, H. M. E. Duyvesteyn, H. M. Ginn, A. J. Mentzer, A. Tuekprakhon, R. Nutalai, B. Wang, A. Dijokaite, S. Khan, O. Avinoam, M. Bahar, D. Skelly, S. Adele, S. A. Johnson, A. Amini, T. G. Ritter, C.

Mason, C. Dold, D. Pan, S. Assadi, A. Bellass, N. Omo-Dare, D. Koeckerling, A. Flaxman, D. Jenkin, P. K. Aley, M. Voysey, S. A. Costa Clemens, F. G. Naveca, V. Nascimento, F. Nascimento, C. F. da Costa, P. C. Resende, A. Pauvolid-Correa, M. M. Siqueira, V. Baillie, N. Serafin, G. Kwatra, K. Da Silva, S. A. Madhi, M. C. Nunes, T. Malik, P. J. M. Openshaw, J. K. Baillie, M. G. Semple, A. R. Townsend, K.-Y. A. Huang, T. K. Tan, M. W. Carroll, P. Klenerman, E. Barnes, S. J. Dunachie, B. Constantinides, H. Webster, D. Crook, A. J. Pollard, T. Lambe, OPTIC Consortium, ISARIC4C Consortium, N. G. Paterson, M. A. Williams, D. R. Hall, E. E. Fry, J. Mongkolsapaya, J. Ren, G. Schreiber, D. I. Stuart, G. R. Screaton, SARS-CoV-2 Omicron-B.1.1.529 leads to widespread escape from neutralizing antibody responses. *Cell* **185**, 467–484.e15 (2022). doi:10.1016/j.cell.2021.12.046 Medline

18. T. N. Starr, A. J. Greaney, W. W. Hannon, A. N. Loes, K. Hauser, J. R. Dillen, E. Ferri, A. G. Farrell, B. Dadonaite, M. McCallum, K. A. Matreyek, D. Corti, D. Veesler, G. Snell, J. D. Bloom, Shifting mutational constraints in the SARS-CoV-2 receptor-binding domain during viral evolution. *Science* **377**, 420–424 (2022). doi:10.1126/science.abo7896 Medline

19. R. Suzuki, D. Yamasoba, I. Kimura, L. Wang, M. Kishimoto, J. Ito, Y. Morioka, N. Nao, H. Nasser, K. Uriu, Y. Kosugi, M. Tsuda, Y. Orba, M. Sasaki, R. Shimizu, R. Kawabata, K. Yoshimatsu, H. Asakura, M. Nagashima, K. Sadamasu, K. Yoshimura, Genotype to Phenotype Japan (G2P-Japan) Consortium, H. Sawa, T. Ikeda, T. Irie, K. Matsuno, S. Tanaka, T. Fukuhara, K. Sato, Attenuated fusogenicity and pathogenicity of SARS-CoV-2 Omicron variant. *Nature* **603**, 700–705 (2022). doi:10.1038/s41586-022-04462-1 Medline

20. D. Yamasoba, I. Kimura, H. Nasser, Y. Morioka, N. Nao, J. Ito, K. Uriu, M. Tsuda, J. Zahradnik, K. Shirakawa, R. Suzuki, M. Kishimoto, Y. Kosugi, K. Kobiyama, T. Hara, M. Toyoda, Y. L. Tanaka, E. P. Butlertanaka, R. Shimizu, H. Ito, L. Wang, Y. Oda, Y. Orba, M. Sasaki, K. Nagata, K. Yoshimatsu, H. Asakura, M. Nagashima, K. Sadamasu, K. Yoshimura, J. Kuramochi, M. Seki, R. Fujiki, A. Kaneda, T. Shimada, T.-a. Nakada, S. Sakao, T. Suzuki, T. Ueno, A. Takaori-Kondo, K. J. Ishii, G. Schreiber, Genotype to Phenotype Japan (G2P-Japan) Consortium, H. Sawa, A. Saito, T. Irie, S. Tanaka, K. Matsuno, T. Fukuhara, T. Ikeda, K. Sato, Virological characteristics of the SARS-CoV-2 Omicron BA.2 spike. *Cell* **185**, 2103–2115.e19 (2022). doi:10.1016/j.cell.2022.04.035 Medline

21. H. Tegally, M. Moir, J. Everatt, M. Giovanetti, C. Scheepers, E. Wilkinson, K. Subramoney, S. Moyo, D. G. Amoako, C. Baxter, C. L. Althaus, U. J. Anyaneji, D. Kekana, R. Viana, J. Giandhari, R. J. Lessells, T. Maponga, D. Maruapula, W. Choga, M. Matshaba, S. Mayaphi, N. Mbhele, M. B. Mbulawa, N. Msomi, NGS-SA consortium, Y. Naidoo, S. Pillay, T. J. Sanko, J. E. San, L. Scott, L. Singh, N. A. Magini, P. Smith-Lawrence, W. Stevens, G. Dor, D. Tshiabuila, N. Wolter, W. Preiser, F. K. Treurnicht, M. Venter, M. Davids, G. Chiloane, A. Mendes, C. McIntyre, A. O'Toole, C. Ruis, T. P. Peacock, C. Roemer, C. Williamson, O. G. Pybus, J. Bhiman, A. Glass, D. P. Martin, A. Rambaut, S. Gaseitsiwe, A. von Gottberg, T. de Oliveira, Continued emergence and evolution of Omicron in South Africa: New BA.4 and BA.5 lineages. medRxiv 2022.05.01.22274406 [Preprint] (2022). https://doi.org/10.1101/2022.05.01.22274406.

22. A. C. Walls, Y.-J. Park, M. A. Tortorici, A. Wall, A. T. McGuire, D. Veesler, Structure, Function, and Antigenicity of the SARS-CoV-2 Spike Glycoprotein. *Cell* **181**, 281–292.e6 (2020). doi:10.1016/j.cell.2020.02.058 Medline

23. M. Hoffmann, H. Kleine-Weber, S. Schroeder, N. Krüger, T. Herrler, S. Erichsen, T. S. Schiergens, G. Herrler, N.-H. Wu, A. Nitsche, M. A. Müller, C. Drosten, S. Pöhlmann, SARS-CoV-2 Cell Entry Depends on ACE2 and TMPRSS2 and Is Blocked by a Clinically Proven Protease Inhibitor. *Cell* **181**, 271–280.e8 (2020). doi:10.1016/j.cell.2020.02.052 Medline

24. P. Zhou, X.-L. Yang, X.-G. Wang, B. Hu, L. Zhang, W. Zhang, H.-R. Si, Y. Zhu, B. Li, C.-L. Huang, H.-D. Chen, J. Chen, Y. Luo, H. Guo, R.-D. Jiang, M.-Q. Liu, Y. Chen, X.-R. Shen, X. Wang, X.-S. Zheng, K. Zhao, Q.-J. Chen, F. Deng, L.-L. Liu, B. Yan, F.-X. Zhan, Y.-Y. Wang, G.-F. Xiao, Z.-L. Shi, A pneumonia outbreak associated with a new coronavirus of probable bat origin. *Nature* **579**, 270–273 (2020). doi:10.1038/s41586-020-2012-7 Medline

25. M. Letko, A. Marzi, V. Munster, Functional assessment of cell entry and receptor usage for SARS-CoV-2 and other lineage B betacoronaviruses. *Nat. Microbiol.* **5**, 562–569 (2020). doi:10.1038/s41564-020-0688-y Medline

26. D. Wrapp, N. Wang, K. S. Corbett, J. A. Goldsmith, C.-L. Hsieh, O. Abiona, B. S. Graham, J. S. McLellan, Cryo-EM structure of the 2019-nCoV spike in the prefusion conformation. *Science* **367**, 1260–1263 (2020). doi:10.1126/science.abb2507 Medline

27. A. C. Walls, M. A. Tortorici, J. Snijder, X. Xiong, B.-J. Bosch, F. A. Rey, D. Veesler, Tectonic conformational changes of a coronavirus spike glycoprotein promote membrane fusion. *Proc. Natl. Acad. Sci. U.S.A.* **114**, 11157–11162 (2017). doi:10.1073/pnas.1708727114 Medline

28. Y. Cai, J. Zhang, T. Xiao, H. Peng, S. M. Sterling, R. M. Walsh Jr., S. Rawson, S. Rits-Volloch, B. Chen, Distinct conformational states of SARS-CoV-2 spike protein. *Science* **369**, 1586–1592 (2020). doi:10.1126/science.abd4251 Medline

29. A. C. Walls, X. Xiong, Y. J. Park, M. A. Tortorici, J. Snijder, J. Quispe, E. Cameroni, R. Gopal, M. Dai, A. Lanzavecchia, M. Zambon, F. A. Rey, D. Corti, D. Veesler, Unexpected Receptor Functional Mimicry Elucidates Activation of Coronavirus Fusion. *Cell* **176**, 1026–1039.e15 (2019). doi:10.1016/j.cell.2018.12.028 Medline

30. P. S. Arunachalam, A. C. Walls, N. Golden, C. Atyeo, S. Fischinger, C. Li, P. Aye, M. J. Navarro, L. Lai, V. V. Edara, K. Röltgen, K. Rogers, L. Shirreff, D. E. Ferrell, S. Wrenn, D. Pettie, J. C. Kraft, M. C. Miranda, E. Kepl, C. Sydeman, N. Brunette, M. Murphy, B. Fiala, L. Carter, A. G. White, M. Trisal, C.-L. Hsieh, K. Russell-Lodrigue, C. Monjure, J. Dufour, S. Spencer, L. Doyle-Meyers, R. P. Bohm, N. J. Maness, C. Roy, J. A. Plante, K. S. Plante, A. Zhu, M. J. Gorman, S. Shin, X. Shen, J. Fontenot, S. Gupta, D. T. O'Hagan, R. Van Der Most, R. Rappuoli, R. L. Coffman, D. Novack, J. S. McLellan, S. Subramaniam, D. Montefiori, S. D. Boyd, J. L. Flynn, G. Alter, F. Villinger, H. Kleanthous, J. Rappaport, M. S. Suthar, N. P. King, D. Veesler, B. Pulendran, Adjuvanting a subunit COVID-19 vaccine to induce protective immunity. *Nature* **594**, 253–258 (2021). doi:10.1038/s41586-021-03530-2 Medline

31. K. McMahan, J. Yu, N. B. Mercado, C. Loos, L. H. Tostanoski, A. Chandrashekar, J. Liu, L. Peter, C. Atyeo, A. Zhu, E. A. Bondzie, G. Dagotto, M. S. Gebre, C. Jacob-Dolan, Z. Li, F. Nampanya, S. Patel, L. Pessaint, A. Van Ry, K. Blade, J. Yalley-Ogunro, M. Cabus, R. Brown, A. Cook, E. Teow, H. Andersen, M. G. Lewis, D. A. Lauffenburger, G. Alter, D. H. Barouch, Correlates of protection against SARS-CoV-2 in rhesus macaques. *Nature* **590**, 630–634 (2021). doi:10.1038/s41586-020-03041-6 Medline

32. D. S. Khoury, D. Cromer, A. Reynaldi, T. E. Schlub, A. K. Wheatley, J. A. Juno, K. Subbarao, S. J. Kent, J. A. Triccas, M. P. Davenport, Neutralizing antibody levels are highly predictive of immune protection from symptomatic SARS-CoV-2 infection. *Nat. Med.* **27**, 1205–1211 (2021). doi:10.1038/s41591-021-01377-8 Medline

33. K. S. Corbett, M. C. Nason, B. Flach, M. Gagne, S. O'Connell, T. S. Johnston, S. N. Shah, V. V. Edara, K. Floyd, L. Lai, C. McDanal, J. R. Francica, B. Flynn, K. Wu, A. Choi, M. Koch, O. M. Abiona, A. P. Werner, J. I. Moliva, S. F. Andrew, M. M. Donaldson, J. Fintzi, D. R. Flebbe, E. Lamb, A. T. Noe, S. T. Nurmukhambetova, S. J. Provost, A. Cook, A. Dodson, A. Faudree, J. Greenhouse, S. Kar, L. Pessaint, M. Porto, K. Steingrebe, D. Valentin, S. Zouantcha, K. W. Bock, M. Minai, B. M. Nagata, R. van de Wetering, S. Boyoglu-Barnum, K. Leung, W. Shi, E. S. Yang, Y. Zhang, J. M. Todd, L. Wang, G. S. Alvarado, H. Andersen, K. E. Foulds, D. K. Edwards, J. R. Mascola, I. N. Moore, M. G. Lewis, A. Carfi, D. Montefiori, M. S. Suthar, A. McDermott, M. Roederer, N. J. Sullivan, D. C. Douek, B. S. Graham, R. A. Seder, Immune correlates of protection by mRNA-1273 vaccine against SARS-CoV-2 in nonhuman primates. *Science* **373**, eabj0299 (2021). doi:10.1126/science.abj0299 Medline

34. P. B. Gilbert, D. C. Montefiori, A. B. McDermott, Y. Fong, D. Benkeser, W. Deng, H. Zhou, C. R. Houchens, K. Martins, L. Jayashankar, F. Castellino, B. Flach, B. C. Lin, S. O'Connell, C. McDanal, A. Eaton, M. Sarzotti-Kelsoe, Y. Lu, C. Yu, B. Borate, L. W. P. van der Laan, N. S. Hejazi, C. Huynh, J. Miller, H. M. El Sahly, L. R. Baden, M. Baron, L. De La Cruz, C. Gay, S. Kalams, C. F. Kelley, M. P. Andrasik, J. G. Kublin, L. Corey, K. M. Neuzil, L. N. Carpp, R. Pajon, D. Follmann, R. O. Donis, R. A. Koup, Immune Assays Team, Moderna, Inc. Team, Coronavirus Vaccine Prevention Network (CoVPN)/Coronavirus Efficacy (COVE) Team, United States Government (USG)/CoVPN Biostatistics Team, Immune correlates analysis of the mRNA-1273 COVID-19 vaccine efficacy clinical trial. *Science* **375**, 43–50 (2022). doi:10.1126/science.abm3425 Medline

35. D. Corti, L. A. Purcell, G. Snell, D. Veesler, Tackling COVID-19 with neutralizing monoclonal antibodies. *Cell* **184**, 3086–3108 (2021). doi:10.1016/j.cell.2021.05.005 Medline

36. L. Piccoli, Y. J. Park, M. A. Tortorici, N. Czudnochowski, A. C. Walls, M. Beltramello, C. Silacci-Fregni, D. Pinto, L. E. Rosen, J. E. Bowen, O. J. Acton, S. Jaconi, B. Guarino, A. Minola, F. Zatta, N. Sprugasci, J. Bassi, A. Peter, A. De Marco, J. C. Nix, F. Mele, S. Jovic, B. F. Rodriguez, S. V. Gupta, F. Jin, G. Piumatti, G. Lo Presti, A. F. Pellanda, M. Biggiogero, M. Tarkowski, M. S. Pizzuto, E. Cameroni, C. Havenar-Daughton, M. Smithey, D. Hong, V. Lepori, E. Albanese, A. Ceschi, E. Bernasconi, L. Elzi, P. Ferrari, C. Garzoni, A. Riva, G. Snell, F. Sallusto, K. Fink, H. W. Virgin, A. Lanzavecchia, D. Corti, D. Veesler, Mapping Neutralizing and Immunodominant Sites on the SARS-CoV-

2 Spike Receptor-Binding Domain by Structure-Guided High-Resolution Serology. *Cell* **183**, 1024–1042.e21 (2020). doi:10.1016/j.cell.2020.09.037 Medline

37. L. Stamatatos, J. Czartoski, Y.-H. Wan, L. J. Homad, V. Rubin, H. Glantz, M. Neradilek, E. Seydoux, M. F. Jennewein, A. J. MacCamy, J. Feng, G. Mize, S. C. De Rosa, A. Finzi, M. P. Lemos, K. W. Cohen, Z. Moodie, M. J. McElrath, A. T. McGuire, mRNA vaccination boosts cross-variant neutralizing antibodies elicited by SARS-CoV-2 infection. *Science* **372**, 1413–1418 (2021). doi:10.1126/science.abg9175 Medline

38. A. J. Greaney, A. N. Loes, L. E. Gentles, K. H. D. Crawford, T. N. Starr, K. D. Malone, H. Y. Chu, J. D. Bloom, Antibodies elicited by mRNA-1273 vaccination bind more broadly to the receptor binding domain than do those from SARS-CoV-2 infection. *Sci. Transl. Med.* **13**, eabi9915 (2021). doi:10.1126/scitranslmed.abi9915 Medline

39. J. E. Bowen, A. C. Walls, A. Joshi, K. R. Sprouse, C. Stewart, M. A. Tortorici, N. M. Franko, J. K. Logue, I. G. Mazzitelli, S. W. Tiles, K. Ahmed, A. Shariq, G. Snell, N. T. Iqbal, J. Geffner, A. Bandera, A. Gori, R. Grifantini, H. Y. Chu, W. C. Van Voorhis, D. Corti, D. Veesler, SARS-CoV-2 spike conformation determines plasma neutralizing activity. bioRxiv 2021.12.19.473391 [Preprint] (2021). https://doi.org/10.1101/2021.12.19.473391.

40. A. C. Walls, M. C. Miranda, A. Schäfer, M. N. Pham, A. Greaney, P. S. Arunachalam, M.-J. Navarro, M. A. Tortorici, K. Rogers, M. A. O’Connor, L. Shirreff, D. E. Ferrell, J. Bowen, N. Brunette, E. Kepl, S. K. Zepeda, T. Starr, C.-L. Hsieh, B. Fiala, S. Wrenn, D. Pettie, C. Sydeman, K. R. Sprouse, M. Johnson, A. Blackstone, R. Ravichandran, C. Ogohara, L. Carter, S. W. Tilles, R. Rappuoli, S. R. Leist, D. R. Martinez, M. Clark, R. Tisch, D. T. O’Hagan, R. Van Der Most, W. C. Van Voorhis, D. Corti, J. S. McLellan, H. Kleanthous, T. P. Sheahan, K. D. Smith, D. H. Fuller, F. Villinger, J. Bloom, B. Pulendran, R. S. Baric, N. P. King, D. Veesler, Elicitation of broadly protective sarbecovirus immunity by receptor-binding domain nanoparticle vaccines. *Cell* **184**, 5432–5447.e16 (2021). doi:10.1016/j.cell.2021.09.015 Medline

41. A. C. Walls, B. Fiala, A. Schäfer, S. Wrenn, M. N. Pham, M. Murphy, L. V. Tse, L. Shehata, M. A. O’Connor, C. Chen, M. J. Navarro, M. C. Miranda, D. Pettie, R. Ravichandran, J. C. Kraft, C. Ogohara, A. Palser, S. Chalk, E. C. Lee, K. Guerriero, E. Kepl, C. M. Chow, C. Sydeman, E. A. Hodge, B. Brown, J. T. Fuller, K. H. Dinnon 3rd, L. E. Gralinski, S. R. Leist, K. L. Gully, T. B. Lewis, M. Guttman, H. Y. Chu, K. K. Lee, D. H. Fuller, R. S. Baric, P. Kellam, L. Carter, M. Pepper, T. P. Sheahan, D. Veesler, N. P. King, Elicitation of Potent Neutralizing Antibody Responses by Designed Protein Nanoparticle Vaccines for SARS-CoV-2. *Cell* **183**, 1367–1382.e17 (2020). doi:10.1016/j.cell.2020.10.043 Medline

42. M. Kodaka, Z. Yang, K. Nakagawa, J. Maruyama, X. Xu, A. Sarkar, A. Ichimura, Y. Nasu, T. Ozawa, H. Iwasa, M. Ishigami-Yuasa, S. Ito, H. Kagechika, Y. Hata, A new cell-based assay to evaluate myogenesis in mouse myoblast C2C12 cells. *Exp. Cell Res.* **336**, 171–181 (2015). doi:10.1016/j.yexcr.2015.06.015 Medline

43. F. A. Lempp, L. B. Soriaga, M. Montiel-Ruiz, F. Benigni, J. Noack, Y.-J. Park, S. Bianchi, A. C. Walls, J. E. Bowen, J. Zhou, H. Kaiser, A. Joshi, M. Agostini, M. Meury, E. Dellota Jr., S. Jaconi, E. Cameroni, J. Martinez-Picado, J. Vergara-Alert, N. Izquierdo-

Useros, H. W. Virgin, A. Lanzavecchia, D. Veesler, L. A. Purcell, A. Telenti, D. Corti, Lectins enhance SARS-CoV-2 infection and influence neutralizing antibodies. *Nature* **598**, 342–347 (2021). doi:10.1038/s41586-021-03925-1 Medline

44. Z. Zhang, J. Mateus, C. H. Coelho, J. M. Dan, C. R. Moderbacher, R. I. Gálvez, F. H. Cortes, A. Grifoni, A. Tarke, J. Chang, E. A. Escarrega, C. Kim, B. Goodwin, N. I. Bloom, A. Frazier, D. Weiskopf, A. Sette, S. Crotty, Humoral and cellular immune memory to four COVID-19 vaccines. *Cell* **185**, 2434–2451.e17 (2022). doi:10.1016/j.cell.2022.05.022

45. D. H. Barouch, K. E. Stephenson, J. Sadoff, J. Yu, A. Chang, M. Gebre, K. McMahan, J. Liu, A. Chandrashekar, S. Patel, M. Le Gars, A. M. de Groot, D. Heerwegh, F. Struyf, M. Douoguih, J. van Hoof, H. Schuitemaker, Durable Humoral and Cellular Immune Responses 8 Months after Ad26.COV2.S Vaccination. *N. Engl. J. Med.* **385**, 951–953 (2021). doi:10.1056/NEJMc2108829 Medline

46. T. A. Bates, S. K. McBride, B. Winders, D. Schoen, L. Trautmann, M. E. Curlin, F. G. Tafesse, Antibody Response and Variant Cross-Neutralization After SARS-CoV-2 Breakthrough Infection. *JAMA* **327**, 179–181 (2022). doi:10.1001/jama.2021.22898 Medline

47. A.-r. Y. Collier, C. M. Brown, K. A. McMahan, J. Yu, J. Liu, C. Jacob-Dolan, A. Chandrashekar, D. Tierney, J. L. Ansel, M. Rowe, D. Sellers, K. Ahmad, R. Aguayo, T. Anioke, S. Gardner, M. Siamatu, L. Bermudez-Rivera, M. R. Hacker, L. C. Madoff, D. H. Barouch, Characterization of immune responses in fully vaccinated individuals after breakthrough infection with the SARS-CoV-2 delta variant. *Sci. Transl. Med.* **14**, eabn6150 (2022). doi:10.1126/scitranslmed.abn6150 Medline

48. Y.-J. Park, D. Pinto, A. C. Walls, Z. Liu, A. De Marco, F. Benigni, F. Zatta, C. Silacci-Fregni, J. Bassi, K. R. Sprouse, A. Addetia, J. E. Bowen, C. Stewart, M. Giurdanella, C. Saliba, B. Guarino, M. A. Schmid, N. Franko, J. Logue, H. V. Dang, K. Hauser, J. di Iulio, W. Rivera, G. Schnell, F. A. Lempp, J. Janer, R. Abdelnabi, P. Maes, P. Ferrari, A. Ceschi, O. Giannini, G. D. de Melo, L. Kergoat, H. Bourhy, J. Neyts, L. Soriagha, L. A. Purcell, G. Snell, S. P. J. Whelan, A. Lanzavecchia, H. W. Virgin, L. Piccoli, H. Chu, M. S. Pizzuto, D. Corti, D. Veesler, Imprinted antibody responses against SARS-CoV-2 Omicron sublineages. bioRxiv 2022.05.08.491108 [Preprint] (2022). https://doi.org/10.1101/2022.05.08.491108.

49. W. F. Garcia-Beltran, K. J. St. Denis, A. Hoelzemer, E. C. Lam, A. D. Nitido, M. L. Sheehan, C. Berrios, O. Ofoman, C. C. Chang, B. M. Hauser, J. Feldman, A. L. Roederer, D. J. Gregory, M. C. Poznansky, A. G. Schmidt, A. J. Iafrate, V. Naranbhai, A. B. Balazs, mRNA-based COVID-19 vaccine boosters induce neutralizing immunity against SARS-CoV-2 Omicron variant. *Cell* **185**, 457–466.e4 (2022). doi:10.1016/j.cell.2021.12.033 Medline

50. K. H. D. Crawford, R. Eguia, A. S. Dingens, A. N. Loes, K. D. Malone, C. R. Wolf, H. Y. Chu, M. A. Tortorici, D. Veesler, M. Murphy, D. Pettie, N. P. King, A. B. Balazs, J. D. Bloom, Protocol and Reagents for Pseudotyping Lentiviral Particles with SARS-CoV-2 Spike Protein for Neutralization Assays. *Viruses* **12**, 513 (2020). doi:10.3390/v12050513 Medline

51. M. Hoffmann, H. Kleine-Weber, S. Pöhlmann, A Multibasic Cleavage Site in the Spike Protein of SARS-CoV-2 Is Essential for Infection of Human Lung Cells. *Mol. Cell* **78**, 779–784.e5 (2020). doi:10.1016/j.molcel.2020.04.022 Medline

52. K. Wu, A. P. Werner, M. Koch, A. Choi, E. Narayanan, G. B. E. Stewart-Jones, T. Colpitts, H. Bennett, S. Boyoglu-Barnum, W. Shi, J. I. Moliva, N. J. Sullivan, B. S. Graham, A. Carfi, K. S. Corbett, R. A. Seder, D. K. Edwards, Serum Neutralizing Activity Elicited by mRNA-1273 Vaccine. *N. Engl. J. Med.* **384**, 1468–1470 (2021). doi:10.1056/NEJMc2102179 Medline

53. K. S. Corbett, D. K. Edwards, S. R. Leist, O. M. Abiona, S. Boyoglu-Barnum, R. A. Gillespie, S. Himansu, A. Schäfer, C. T. Ziwawo, A. T. DiPiazza, K. H. Dinnon, S. M. Elbashir, C. A. Shaw, A. Woods, E. J. Fritch, D. R. Martinez, K. W. Bock, M. Minai, B. M. Nagata, G. B. Hutchinson, K. Wu, C. Henry, K. Bahl, D. Garcia-Dominguez, L. Ma, I. Renzi, W.-P. Kong, S. D. Schmidt, L. Wang, Y. Zhang, E. Phung, L. A. Chang, R. J. Loomis, N. E. Altaras, E. Narayanan, M. Metkar, V. Presnyak, C. Liu, M. K. Louder, W. Shi, K. Leung, E. S. Yang, A. West, K. L. Gully, L. J. Stevens, N. Wang, D. Wrapp, N. A. Doria-Rose, G. Stewart-Jones, H. Bennett, G. S. Alvarado, M. C. Nason, T. J. Ruckwardt, J. S. McLellan, M. R. Denison, J. D. Chappell, I. N. Moore, K. M. Morabito, J. R. Mascola, R. S. Baric, A. Carfi, B. S. Graham, SARS-CoV-2 mRNA vaccine design enabled by prototype pathogen preparedness. *Nature* **586**, 567–571 (2020). doi:10.1038/s41586-020-2622-0 Medline

54. E. E. Walsh, R. W. Frenck Jr., A. R. Falsey, N. Kitchin, J. Absalon, A. Gurtman, S. Lockhart, K. Neuzil, M. J. Mulligan, R. Bailey, K. A. Swanson, P. Li, K. Koury, W. Kalina, D. Cooper, C. Fontes-Garfias, P.-Y. Shi, Ö. Türeci, K. R. Tompkins, K. E. Lyke, V. Raabe, P. R. Dormitzer, K. U. Jansen, U. Şahin, W. C. Gruber, Safety and Immunogenicity of Two RNA-Based Covid-19 Vaccine Candidates. *N. Engl. J. Med.* **383**, 2439–2450 (2020). doi:10.1056/NEJMoa2027906 Medline

55. D. Planas, N. Saunders, P. Maes, F. Guivel-Benhassine, C. Planchais, J. Buchrieser, W.-H. Bolland, F. Porrot, I. Staropoli, F. Lemoine, H. Péré, D. Veyer, J. Puech, J. Rodary, G. Baela, S. Dellicour, J. Raymenants, S. Gorissen, C. Geenen, B. Vanmechelen, T. Wawina-Bokalanga, J. Martí-Carreras, L. Cuypers, A. Sève, L. Hocqueloux, T. Prazuck, F. A. Rey, E. Simon-Lorriere, T. Bruel, H. Mouquet, E. André, O. Schwartz, Considerable escape of SARS-CoV-2 Omicron to antibody neutralization. *Nature* **602**, 671–675 (2022). doi:10.1038/d41586-021-03827-2 Medline

56. E. Pérez-Then, C. Lucas, V. S. Monteiro, M. Miric, V. Brache, L. Cochon, C. B. F. Vogels, A. A. Malik, E. De la Cruz, A. Jorge, M. De Los Santos, P. Leon, M. I. Breban, K. Billig, I. Yildirim, C. Pearson, R. Downing, E. Gagnon, A. Muyombwe, J. Razeq, M. Campbell, A. I. Ko, S. B. Omer, N. D. Grubaugh, S. H. Vermund, A. Iwasaki, Neutralizing antibodies against the SARS-CoV-2 Delta and Omicron variants following heterologous CoronaVac plus BNT162b2 booster vaccination. *Nat. Med.* **28**, 481–485 (2022). doi:10.1038/s41591-022-01705-6 Medline

57. R. R. Goel, M. M. Painter, K. A. Lundgreen, S. A. Apostolidis, A. E. Baxter, J. R. Giles, D. Mathew, A. Pattekar, A. Reynaldi, D. S. Khoury, S. Gouma, P. Hicks, S. Dysinger, A. Hicks, H. Sharma, S. Herring, S. Korte, K. C. Wumesh, D. A. Oldridge, R. I. Erickson, M. E. Weirick, C. M. McAllister, M. Awofolaju, N. Tanenbaum, J. Dougherty, S. Long,

K. D'Andrea, J. T. Hamilton, M. McLaughlin, J. C. Williams, S. Adamski, O. Kuthuru, E. M. Drapeau, M. P. Davenport, S. E. Hensley, P. Bates, A. R. Greenplate, E. J. Wherry, Efficient recall of Omicron-reactive B cell memory after a third dose of SARS-CoV-2 mRNA vaccine. *Cell* **185**, 1875–1887.e8 (2022). doi:10.1016/j.cell.2022.04.009 Medline

58. F. Muecksch, Z. Wang, A. Cho, C. Gaebler, T. B. Tanfous, J. DaSilva, E. Bednarski, V. Ramos, S. Zong, B. Johnson, R. Raspe, D. Schaefer-Babajew, I. Shimeliovich, M. Daga, K.-H. Yao, F. Schmidt, K. G. Millard, M. Turroja, M. Jankovic, T. Y. Oliveria, A. Gazumyan, M. Caskey, T. Hatziioannou, P. D. Bieniasz, M. C. Nussenzweig, Increased Potency and Breadth of SARS-CoV-2 Neutralizing Antibodies After a Third mRNA Vaccine Dose. bioRxiv 2022.02.14.480394 [Preprint] (2022). https://doi.org/10.1101/2022.02.14.480394.

59. B. Grunau, D. M. Goldfarb, M. Asamoah-Boaheng, L. Golding, T. L. Kirkham, P. A. Demers, P. M. Lavoie, Immunogenicity of Extended mRNA SARS-CoV-2 Vaccine Dosing Intervals. *JAMA* **327**, 279–281 (2022). doi:10.1001/jama.2021.21921 Medline

60. M. Voysey, S. A. Costa Clemens, S. A. Madhi, L. Y. Weckx, P. M. Folegatti, P. K. Aley, B. Angus, V. L. Baillie, S. L. Barnabas, Q. E. Bhorat, S. Bibi, C. Briner, P. Cicconi, E. A. Clutterbuck, A. M. Collins, C. L. Cutland, T. C. Darton, K. Dheda, C. Dold, C. J. A. Duncan, K. R. W. Emary, K. J. Ewer, A. Flaxman, L. Fairlie, S. N. Faust, S. Feng, D. M. Ferreira, A. Finn, E. Galiza, A. L. Goodman, C. M. Green, C. A. Green, M. Greenland, C. Hill, H. C. Hill, I. Hirsch, A. Izu, D. Jenkin, C. C. D. Joe, S. Kerridge, A. Koen, G. Kwatra, R. Lazarus, V. Libri, P. J. Lillie, N. G. Marchevsky, R. P. Marshall, A. V. A. Mendes, E. P. Milan, A. M. Minassian, A. McGregor, Y. F. Mujadidi, A. Nana, S. D. Padayachee, D. J. Phillips, A. Pittella, E. Plested, K. M. Pollock, M. N. Ramasamy, A. J. Ritchie, H. Robinson, A. V. Schwarzbold, A. Smith, R. Song, M. D. Snape, E. Sprinz, R. K. Sutherland, E. C. Thomson, M. E. Török, M. Toshner, D. P. J. Turner, J. Vekemans, T. L. Villafana, T. White, C. J. Williams, A. D. Douglas, A. V. S. Hill, T. Lambe, S. C. Gilbert, A. J. Pollard, Oxford COVID Vaccine Trial Group, Single-dose administration and the influence of the timing of the booster dose on immunogenicity and efficacy of ChAdOx1 nCoV-19 (AZD1222) vaccine: A pooled analysis of four randomised trials. *Lancet* **397**, 881–891 (2021). doi:10.1016/S0140-6736(21)00432-3 Medline

61. H. Parry, R. Bruton, C. Stephens, C. Bentley, K. Brown, G. Amirthalingam, B. Hallis, A. Otter, J. Zuo, P. Moss, Extended interval BNT162b2 vaccination enhances peak antibody generation. *NPJ Vaccines* **7**, 14 (2022). doi:10.1038/s41541-022-00432-w Medline

62. R. P. Payne, S. Longet, J. A. Austin, D. T. Skelly, W. Dejnirattisai, S. Adele, N. Meardon, S. Faustini, S. Al-Taei, S. C. Moore, T. Tipton, L. M. Hering, A. Angyal, R. Brown, A. R. Nicols, N. Gillson, S. L. Dobson, A. Amini, P. Supasa, A. Cross, A. Bridges-Webb, L. S. Reyes, A. Linder, G. Sandhar, J. A. Kilby, J. K. Tyerman, T. Altmann, H. Hornsby, R. Whitham, E. Phillips, T. Malone, A. Hargreaves, A. Shields, A. Saei, S. Foulkes, L. Stafford, S. Johnson, D. G. Wootton, C. P. Conlon, K. Jeffery, P. C. Matthews, J. Frater, A. S. Deeks, A. J. Pollard, A. Brown, S. L. Rowland-Jones, J. Mongkolsapaya, E. Barnes, S. Hopkins, V. Hall, C. Dold, C. J. A. Duncan, A. Richter, M. Carroll, G. Screaton, T. I. de Silva, L. Turtle, P. Klenerman, S. Dunachie, PITCH Consortium, Immunogenicity of standard and extended dosing intervals of BNT162b2 mRNA vaccine. *Cell* **184**, 5699–5714.e11 (2021). doi:10.1016/j.cell.2021.10.011 Medline

63. J. Liu, A. Chandrashekar, D. Sellers, J. Barrett, C. Jacob-Dolan, M. Lifton, K. McMahan, M. Sciacca, H. VanWyk, C. Wu, J. Yu, A. Y. Collier, D. H. Barouch, Vaccines elicit highly conserved cellular immunity to SARS-CoV-2 Omicron. *Nature* **603**, 493–496 (2022). doi:10.1038/s41586-022-04465-y Medline

64. A. Tarke, C. H. Coelho, Z. Zhang, J. M. Dan, E. D. Yu, N. Methot, N. I. Bloom, B. Goodwin, E. Phillips, S. Mallal, J. Sidney, G. Filaci, D. Weiskopf, R. da Silva Antunes, S. Crotty, A. Grifoni, A. Sette, SARS-CoV-2 vaccination induces immunological T cell memory able to cross-recognize variants from Alpha to Omicron. *Cell* **185**, 847–859.e11 (2022). doi:10.1016/j.cell.2022.01.015 Medline

65. N. Andrews, J. Stowe, F. Kirsebom, S. Toffa, T. Rickeard, E. Gallagher, C. Gower, M. Kall, N. Groves, A.-M. O'Connell, D. Simons, P. B. Blomquist, A. Zaidi, S. Nash, N. Iwani Binti Abdul Aziz, S. Thelwall, G. Dabrera, R. Myers, G. Amirthalingam, S. Gharbia, J. C. Barrett, R. Elson, S. N. Ladhani, N. Ferguson, M. Zambon, C. N. J. Campbell, K. Brown, S. Hopkins, M. Chand, M. Ramsay, J. Lopez Bernal, Covid-19 Vaccine Effectiveness against the Omicron (B.1.1.529) Variant. *N. Engl. J. Med.* **386**, 1532–1546 (2022). doi:10.1056/NEJMoa2119451 Medline

66. E. K. Accorsi, A. Britton, K. E. Fleming-Dutra, Z. R. Smith, N. Shang, G. Derado, J. Miller, S. J. Schrag, J. R. Verani, Association Between 3 Doses of mRNA COVID-19 Vaccine and Symptomatic Infection Caused by the SARS-CoV-2 Omicron and Delta Variants. *JAMA* **327**, 639–651 (2022). doi:10.1001/jama.2022.0470 Medline

67. B. Ying, S. M. Scheaffer, B. Whitener, C.-Y. Liang, O. Dmytrenko, S. Mackin, K. Wu, D. Lee, L. E. Avena, Z. Chong, J. B. Case, L. Ma, T. Kim, C. Sein, A. Woods, D. M. Berrueta, A. Carfi, S. M. Elbashir, D. K. Edwards, L. B. Thackray, M. S. Diamond, Boosting with Omicron-matched or historical mRNA vaccines increases neutralizing antibody responses and protection against B.1.1.529 infection in mice. bioRxiv 2022.02.07.479419 [Preprint] (2022). https://doi.org/10.1101/2022.02.07.479419.

68. I.-J. Lee, C.-P. Sun, P.-Y. Wu, Y.-H. Lan, I.-H. Wang, W.-C. Liu, S.-C. Tseng, S.-I. Tsung, Y.-C. Chou, M. Kumari, Y.-W. Chang, H.-F. Chen, Y.-S. Lin, T.-Y. Chen, C.-W. Chiu, C.-H. Hsieh, C.-Y. Chuang, C.-C. Lin, C.-M. Cheng, H.-T. Lin, W.-Y. Chen, P.-C. Chiang, C.-C. Lee, J. C. Liao, H.-C. Wu, M.-H. Tao, Omicron-specific mRNA vaccine induced potent neutralizing antibody against Omicron but not other SARS-CoV-2 variants. bioRxiv 2022.01.31.478406 [Preprint] (2022). https://doi.org/10.1101/2022.01.31.478406.

69. D. W. Hawman, K. Meade-White, C. Clancy, J. Archer, T. Hinkley, S. S. Leventhal, D. Rao, A. Stamper, M. Lewis, R. Rosenke, K. Krieger, S. Randall, A. P. Khandhar, L. Hao, T.-Y. Hsiang, A. L. Greninger, M. Gale Jr., P. Berglund, D. H. Fuller, K. Rosenke, H. Feldmann, J. H. Erasmus, Replicating RNA platform enables rapid response to the SARS-CoV-2 Omicron variant and elicits enhanced protection in naïve hamsters compared to ancestral vaccine. bioRxiv 2022.01.31.478520 [Preprint] (2022). https://doi.org/10.1101/2022.01.31.478520.

70. P. S. Arunachalam, Y. Feng, U. Ashraf, M. Hu, V. V. Edara, V. I. Zarnitsyna, P. P. Aye, N. Golden, K. W. M. Green, B. M. Threeton, N. J. Maness, B. J. Beddingfield, R. P. Bohm, J. Dufour, K. Russell-Lodrigue, M. C. Miranda, A. C. Walls, K. Rogers, L. Shirreff, D. E.

Ferrell, N. R. Deb Adhikary, J. Fontenot, A. Grifoni, A. Sette, D. T. O'Hagan, R. Van Der Most, R. Rappuoli, F. Villinger, H. Kleanthous, J. Rappaport, M. S. Suthar, D. Veesler, T. T. Wang, N. P. King, B. Pulendran, Durable protection against SARS-CoV-2 Omicron induced by an adjuvanted subunit vaccine. bioRxiv 2022.03.18.484950 [Preprint] (2022). https://doi.org/10.1101/2022.03.18.484950.

71. M. Gagne, J. I. Moliva, K. E. Foulds, S. F. Andrew, B. J. Flynn, A. P. Werner, D. A. Wagner, I.-T. Teng, B. C. Lin, C. Moore, N. Jean-Baptiste, R. Carroll, S. L. Foster, M. Patel, M. Ellis, V.-V. Edara, N. V. Maldonado, M. Minai, L. McCormick, C. C. Honeycutt, B. M. Nagata, K. W. Bock, C. N. M. Dulan, J. Cordon, D. R. Flebbe, J. M. Todd, E. McCarthy, L. Pessaint, A. Van Ry, B. Narvaez, D. Valentin, A. Cook, A. Dodson, K. Steingrebe, S. T. Nurmukhambetova, S. Godbole, A. R. Henry, F. Laboune, J. Roberts-Torres, C. G. Lorang, S. Amin, J. Trost, M. Naisan, M. Basappa, J. Willis, L. Wang, W. Shi, N. A. Doria-Rose, Y. Zhang, E. S. Yang, K. Leung, S. O'Dell, S. D. Schmidt, A. S. Olia, C. Liu, D. R. Harris, G.-Y. Chuang, G. Stewart-Jones, I. Renzi, Y.-T. Lai, A. Malinowski, K. Wu, J. R. Mascola, A. Carfi, P. D. Kwong, D. K. Edwards, M. G. Lewis, H. Andersen, K. S. Corbett, M. C. Nason, A. B. McDermott, M. S. Suthar, I. N. Moore, M. Roederer, N. J. Sullivan, D. C. Douek, R. A. Seder, mRNA-1273 or mRNA-Omicron boost in vaccinated macaques elicits similar B cell expansion, neutralizing responses, and protection from Omicron. *Cell* **185**, 1556–1571.e18 (2022). doi:10.1016/j.cell.2022.03.038 Medline

72. S. I. Richardson, V. S. Madzorera, H. Spencer, N. P. Manamela, M. A. van der Mescht, B. E. Lambson, B. Oosthuysen, F. Ayres, Z. Makhado, T. Moyo-Gwete, N. Mzindle, T. Motlou, A. Strydom, A. Mendes, H. Tegally, Z. de Beer, T. Roma de Villiers, A. Bodenstein, G. van den Berg, M. Venter, T. de Oliviera, V. Ueckermann, T. M. Rossouw, M. T. Boswell, P. L. Moore, SARS-CoV-2 Omicron triggers cross-reactive neutralization and Fc effector functions in previously vaccinated, but not unvaccinated, individuals. *Cell Host Microbe* **30**, 880–886.e4 (2022). doi:10.1016/j.chom.2022.03.029 Medline

73. A. Rössler, L. Knabl, D. von Laer, J. Kimpel, Neutralization profile after recovery from SARS-CoV-2 omicron infection. *N. Engl. J. Med.* **386**, 1764–1766 (2022). doi:10.1056/NEJMc2201607 Medline

74. K. Stiasny, I. Medits, D. Springer, M. Graninger, J. Camp, E. Höltl, S. Aberle, M. Traugott, W. Hoepler, J. Deutsch, O. Lammel, C. Borsodi, E. Puchhammer-Stöckl, A. Zoufaly, L. Weseslindtner, J. Aberle, Human primary Omicron BA.1 and BA.2 infections result in sub-lineage-specific neutralization. Research Square [Preprint] (2022). https://doi.org/10.21203/rs.3.rs-1536794/v1.

75. T. Mao, B. Israelow, A. Suberi, L. Zhou, M. Reschke, M. A. Peña-Hernández, H. Dong, R. J. Homer, W. M. Saltzman, A. Iwasaki, Unadjuvanted intranasal spike vaccine booster elicits robust protective mucosal immunity against sarbecoviruses. bioRxiv 2022.01.24.477597 [Preprint] (2022). https://doi.org/10.1101/2022.01.24.477597.

76. J. E. Oh, E. Song, M. Moriyama, P. Wong, S. Zhang, R. Jiang, S. Strohmeier, S. H. Kleinstein, F. Krammer, A. Iwasaki, Intranasal priming induces local lung-resident B cell populations that secrete protective mucosal antiviral IgA. *Sci. Immunol.* **6**, eabj5129 (2021). doi:10.1126/sciimmunol.abj5129 Medline

77. A. O. Hassan, F. Feldmann, H. Zhao, D. T. Curiel, A. Okumura, T.-L. Tang-Huau, J. B. Case, K. Meade-White, J. Callison, R. E. Chen, J. Lovaglio, P. W. Hanley, D. P. Scott, D. H. Fremont, H. Feldmann, M. S. Diamond, A single intranasal dose of chimpanzee adenovirus-vectored vaccine protects against SARS-CoV-2 infection in rhesus macaques. *Cell Rep. Med.* **2**, 100230 (2021). doi:10.1016/j.xcrm.2021.100230 Medline

78. S. N. Langel, S. Johnson, C. I. Martinez, S. N. Tedjakusuma, N. Peinovich, E. G. Dora, P. J. Kuehl, H. Irshad, E. G. Barrett, A. Werts, S. N. Tucker, Adenovirus type 5 SARS-CoV-2 vaccines delivered orally or intranasally reduced disease severity and transmission in a hamster model. *Sci. Transl. Med.* 10.1126/scitranslmed.abn6868 (2022). doi:10.1126/scitranslmed.abn6868 Medline

79. A. A. Cohen, P. N. P. Gnanapragasam, Y. E. Lee, P. R. Hoffman, S. Ou, L. M. Kakutani, J. R. Keeffe, H.-J. Wu, M. Howarth, A. P. West, C. O. Barnes, M. C. Nussenzweig, P. J. Bjorkman, Mosaic nanoparticles elicit cross-reactive immune responses to zoonotic coronaviruses in mice. *Science* **371**, 735–741 (2021). doi:10.1126/science.abf6840 Medline

80. D. R. Martinez, A. Schäfer, S. R. Leist, G. De la Cruz, A. West, E. N. Atochina-Vasserman, L. C. Lindesmith, N. Pardi, R. Parks, M. Barr, D. Li, B. Yount, K. O. Saunders, D. Weissman, B. F. Haynes, S. A. Montgomery, R. S. Baric, Chimeric spike mRNA vaccines protect against Sarbecovirus challenge in mice. *Science* **373**, 991–998 (2021). doi:10.1126/science.abi4506 Medline

81. J. B. Case, P. W. Rothlauf, R. E. Chen, Z. Liu, H. Zhao, A. S. Kim, L. M. Bloyet, Q. Zeng, S. Tahan, L. Droit, M. X. G. Ilagan, M. A. Tartell, G. Amarasinghe, J. P. Henderson, S. Miersch, M. Ustav, S. Sidhu, H. W. Virgin, D. Wang, S. Ding, D. Corti, E. S. Theel, D. H. Fremont, M. S. Diamond, S. P. J. Whelan, Neutralizing Antibody and Soluble ACE2 Inhibition of a Replication-Competent VSV-SARS-CoV-2 and a Clinical Isolate of SARS-CoV-2. *Cell Host Microbe* **28**, 475–485.e5 (2020). doi:10.1016/j.chom.2020.06.021 Medline

82. X. Ou, Y. Liu, X. Lei, P. Li, D. Mi, L. Ren, L. Guo, R. Guo, T. Chen, J. Hu, Z. Xiang, Z. Mu, X. Chen, J. Chen, K. Hu, Q. Jin, J. Wang, Z. Qian, Characterization of spike glycoprotein of SARS-CoV-2 on virus entry and its immune cross-reactivity with SARS-CoV. *Nat. Commun.* **11**, 1620 (2020). doi:10.1038/s41467-020-15562-9 Medline

83. C. L. Hsieh, J. A. Goldsmith, J. M. Schaub, A. M. DiVenere, H. C. Kuo, K. Javanmardi, K. C. Le, D. Wrapp, A. G. Lee, Y. Liu, C. W. Chou, P. O. Byrne, C. K. Hjorth, N. V. Johnson, J. Ludes-Meyers, A. W. Nguyen, J. Park, N. Wang, D. Amengor, J. J. Lavinder, G. C. Ippolito, J. A. Maynard, I. J. Finkelstein, J. S. McLellan, Structure-based design of prefusion-stabilized SARS-CoV-2 spikes. *Science* **369**, 1501–1505 (2020). doi:10.1126/science.abd0826 Medline
