# Supplementary material for: Omicron spike function and neutralizing activity elicited by a comprehensive panel of vaccines
Source: Science. 2022 Jul 19;377(6608):890–4. doi: 10.1126/science.abq0203 (PMC9348749; doi:10.1126/science.abq0203)
Supplement: Supplementary file 3 — Movies S1 to S6 [file science.abq0203_movies_s1_to_s6.zip › science.abq0203_movie_captions.pdf]

Supplementary Material

Movies S1-S6. Representative movies of real-time cell-cell fusion captured every 30 minutes over a 24-hour period with effector cells expressing SARS-CoV-2 S with the G614 (S1), Delta (S2), BA.1 (S3), BA.2 (S4), BA.2.12.1 (S5), or BA.4/5 (S6) mutations.
